# Multimodal correlative imaging and modelling of phosphorus uptake from soil by hyphae of mycorrhizal fungi

**DOI:** 10.1111/nph.17980

**Published:** 2022-02-15

**Authors:** Sam Keyes, Arjen van Veelen, Dan McKay Fletcher, Callum Scotson, Nico Koebernick, Chiara Petroselli, Katherine Williams, Siul Ruiz, Laura Cooper, Robbie Mayon, Simon Duncan, Marc Dumont, Iver Jakobsen, Giles Oldroyd, Andrzej Tkacz, Philip Poole, Fred Mosselmans, Camelia Borca, Thomas Huthwelker, David L. Jones, Tiina Roose

**Affiliations:** ^1^ Bioengineering Sciences Research Group Department of Mechanical Engineering School of Engineering Faculty of Engineering and Physical Sciences University of Southampton Southampton SO17 1BJ UK; ^2^ Material Science and Technology Division Los Alamos National Laboratory Los Alamos NM 87545 USA; ^3^ Stanford Synchrotron Radiation Lightsource SLAC National Accelerator Laboratory Menlo Park CA 94025 USA; ^4^ School of Biological Sciences University of Southampton Southampton SO17 1BJ UK; ^5^ Department of Plant and Environmental Sciences University of Copenhagen Thorvaldsensvej 40 Frederiksberg DK‐1871 Denmark; ^6^ Crop Science Centre University of Cambridge 93 Lawrence Weaver Road Cambridge CB3 0LE UK; ^7^ Department of Plant Sciences University of Oxford South Parks Road Oxford OX1 3RB UK; ^8^ Diamond Light Source Diamond House, Harwell Science & Innovation Campus Didcot OX11 0DE UK; ^9^ Swiss Light Source PSI Forschungsstrasse 111 Villigen 5232 Switzerland; ^10^ School of Natural Sciences Bangor University Bangor LL57 2DG UK; ^11^ 5673 SoilsWest, Food Futures Institute Murdoch University 90 South Street Murdoch WA 6150 Australia

**Keywords:** mycorrhizas, plant phosphorus uptake, rhizosphere modelling, synchrotron, X‐ray computed tomography, X‐ray fluorescence

## Abstract

- Phosphorus (P) is essential for plant growth. Arbuscular mycorrhizal fungi (AMF) aid its uptake by acquiring P from sources distant from roots in return for carbon. Little is known about how AMF colonise soil pore‐space, and models of AMF‐enhanced P‐uptake are poorly validated.
- We used synchrotron X‐ray computed tomography to visualize mycorrhizas in soil and synchrotron X‐ray fluorescence/X‐ray absorption near edge structure (XRF/XANES) elemental mapping for P, sulphur (S) and aluminium (Al) in combination with modelling.
- We found that AMF inoculation had a suppressive effect on colonisation by other soil fungi and identified differences in structure and growth rate between hyphae of AMF and nonmycorrhizal fungi. Our results showed that AMF co‐locate with areas of high P and low Al, and preferentially associate with organic‐type P species over Al‐rich inorganic P.
- We discovered that AMF avoid Al‐rich areas as a source of P. Sulphur‐rich regions were found to be correlated with higher hyphal density and an increased organic‐associated P‐pool, whilst oxidized S‐species were found close to AMF hyphae. Increased S oxidation close to AMF suggested the observed changes were microbiome‐related. Our experimentally‐validated model led to an estimate of P‐uptake by AMF hyphae that is an order of magnitude lower than rates previously estimated – a result with significant implications for the modelling of plant–soil–AMF interactions.

## Introduction

Mineral phosphorus (P) resources are sparse and unevenly distributed across the world (Gross, 2017). Arbuscular mycorrhizal fungi (AMF) play an important role in mediating plant uptake of P (Smith *et al*., 2003), which is often considered to be a growth‐limiting soil resource (Vaccari, 2009). Mycorrhizal plants supply carbon (C) to AMF mycelia to drive hyphal growth, while in return the hyphae provide P and other nutrients back to the plant. Recent studies have found that in some cases a reduced plant C allocation to AMF does not alter AMF P supply to the plant (Charters *et al*., 2020), which shows promise for crop production when P supply is limiting to growth. Much is known about plant–AMF symbioses on the soil bulk‐scale, but little is known about the spatial distribution of AMF hyphae in soil due to difficulties in visualizing soil pore‐space *in situ*. However, it is important to know how AMF hyphae interact with soil as P is strongly bound to soil surfaces, and pore‐scale processes thus govern AMF‐mediated P uptake. While we can modify certain pore‐scale processes using chemical and microbial soil treatments, it is important to establish the mechanistic basis of symbiont behaviour within the soil matrix.

Sulphur (S), like P, is an essential macronutrient required by plants, and it can also promote hyphal growth (Hepper, 1984). It has the potential to be a surrogate marker for the soil microbial community. Sulphur exists in soils in a wide variety of environmentally‐dependent oxidation states, ranging from −2 to +6, with organo‐S comprising > 90% of the total S pool in most soils (Gene *et al*., 2002; Prietzel *et al*., 2007, 2011). Arbuscular mycorrhizal fungi may play a role in plant S metabolism through uptake and up‐regulation of plant sulphate carriers, and through their interaction with organo‐S mobilizing microbes (Gahan & Schmalenberger, 2014; Berruti *et al*., 2015). Arbuscular mycorrhizal fungi mycelia are surrounded by complex bacterial and fungal communities that both interact with and sustain their metabolic function. Therefore, similar to the area affected by roots (i.e. the rhizosphere), a ‘hyphosphere’ of AMF can be a zone of increased bacterial abundance, and a site of localised biochemical activity (Rozmos *et al*., 2021).

Several groups have modelled the growth of hyphal networks, as reviewed by Boswell & Davidson (2012). These models have addressed cell physics aspects (Bartnicki‐Garcia *et al*., 2000), physiological population growth (Edelstein, 1982), and biochemical processes controlling hyphal growth (Tlalka *et al*., 2003). However, a persistent limitation in such models is a lack of validation due to experimental constraints. As soil is opaque to visible light, direct observation of hyphal morphology without disturbing the soil matrix is challenging. Boswell & Davidson (2012) reviewed models of mycelial development, while also identifying the need for model validation with experimental data. Simard *et al*. (2012) reviewed the ecology and modelling of mycorrhizal fungi and highlighted the fact that a special challenge is the lack of techniques for observing hyphae in soil at a suitable spatial resolution. Some of the first models to specifically include AMF growth in soil and uptake of P were developed by our group (Schnepf & Roose, 2006; Schnepf *et al*., 2008a). However, these models were only validated against data on the bulk soil and plant scales, and the soil P status was not monitored spatiotemporally. This lack of knowledge regarding AMF mycelial architecture on the soil pore‐scale has impeded further investigation of the significance of soil–AMF–plant interactions for P uptake. In this paper we take the first necessary step towards building a fully validated plant–AMF P‐uptake model. This is achieved by imaging AMF structures in the soil pore‐space *in situ* using synchrotron X‐ray computed tomography (SXRCT) in combination with traditional hyphal length measurements as set out by Jakobsen *et al*. (1992b), and correlating these data with spatial profiles of P, S and Al on the same soil samples using X‐ray fluorescence/X‐ray absorption near edge structure (XRF/XANES) imaging. Aluminium profiles are used to distinguish soil‐mineral‐associated P from the organic pool. The structural and chemical imaging results are integrated into a plant–AMF mathematical model of P uptake (Schnepf & Roose, 2006; Schnepf *et al*., 2008a) to further estimate hyphal uptake rates and predict how the plant–AMF symbiosis benefits plant P acquisition. This enables us to image and quantify AMF in soil in three dimensions for the first time, apply finer‐scale limits on P uptake rates by AMF hyphae, and provide the first calibrated mathematical model for AMF hyphae P uptake in soil. The comparison of hyphal length density measurements via SXRCT and a destructive approach also allows us to quantify the differences between two mycelial detection techniques.

## Materials and Methods

### Plant and fungal growth assay

The growth medium was a sand‐textured Eutric Cambisol soil collected from a surface plot at Abergwyngregyn, North Wales, UK (lat. 53°14′N, long. 4°01′W), for which the soil organic matter content was 7%. This soil corresponds to ‘soil B’ in Lucas & Jones (2006). A split‐compartment system was designed to produce soil samples of small cross‐section (∅ < 5 mm) for hyphal compartments, allowing SXRCT imaging at a sufficient spatial resolution to observe hyphal structures (see Supporting Information Methods S1: Figs S1.1, S1.2 therein). The system enabled the growth and maintenance of mycorrhizal wheat plants under controlled conditions for up to 4 wk. A bespoke growth box was designed to maintain both hyphal and root compartments in dark conditions under stable and externally applied water potentials whilst allowing gas exchange and aerial plant growth (see Methods S1 for full design details). A mesh barrier maintained the separation between the root and the hyphal compartments while permitting the transfer of hyphae (Faber *et al*., 1991; Carminati *et al*., 2009) and water between the two. In *P+* treatments, a small solid pellet of triple super phosphate (mean mass 0.0757 ± 0.007 g) was added to each hyphal compartment. In *P−* treatments, no supplemental P was added.

#### Plant‐inoculation treatments

A wheat cultivar *Triticum aestivum* L. cv Apache (WBCDB0003‐PG‐1) provided by the Germplasm Resources Unit of the John Innes Centre (UK) was selected due to its indicated high affinity for colonization by AMF (Leake, 2016). Seeds were surface sterilised and germinated in the dark for 96 h at 23°C. Seedlings were selected for uniform radicle length and transplanted to each of 12 root compartments (2 P treatments × 2 time points × 3 replicates).

Root compartments were inoculated with *Rhizophagus irregularis* (BEG72; PlantWorks Ltd, Henfield, UK); we also tried inoculation with slow growing fungus *Gigaspora rosea*, but as this fungus did not grow, we did not use it for the remainder of the study (Methods S1). The inoculum contained hyphae, spores and colonized root fragments at 1.6 × 10^6^ propagules l^−1^ of an inert zeolite carrier substrate. A uniform inoculating paste was achieved by concentrating the substrate according to manufacturer recommendations (Methods S1: Section 1.3 therein). During the filling of each root compartment, 1.5 g of inoculating paste and 0.5 g of raw inoculum (see also Methods S1: Section 1.3 therein) was added at 25, 65 and 105 mm (d_1_, d_2_ and d_3_, respectively) from the top of each root compartment (Methods S1: Fig. S1.1 therein); this was advised by the manufacturer PlantWorks Ltd to maximise the symbiosis.

The growth boxes were maintained in a climate‐controlled growth chamber (Conviron A1000, Conviron UK, Isleham, UK) with a 14 h : 10 h, 23°C : 18°C, light : dark photoperiod (full light: 700 µmol m^−2^ s^−1^) at 75% humidity. For each treatment, three plant replicates (R_1_, R_2_, R_3_) were grown to 2 and 4 wk after transplantation (i.e. a total of six plants per P condition).

#### Control treatments

The soil was neither autoclaved nor gamma‐irradiated, since this would dramatically alter the natural microbiome and biogeochemical cycling processes occurring within the soil. Hence, the soil was assumed to contain native fungal spores. To prevent AMF propagules from developing hyphae in the controls, these contained no plants. Therefore, the ‘plant’ compartments for the control replicates were filled as outlined in the previous subsection and were lightly sealed with rubber bungs, with the soil water potential of the controls being kept similar to that of the plant compartments.

### Structural imaging

Following a growth period of 14 or 28 d, depending on the treatment, the hyphal compartments were imaged using two separate SXRCT beamlines: The I13 beamline at the Diamond Light Source, UK, and the TOMCAT beamline at the Swiss Light Source (SLS), Switzerland, using a setup described in previous studies (Keyes *et al*., 2013; Koebernick *et al*., 2017, 2019). For each hyphal compartment, three vertical positions were imaged: an *intermediate* position (h_2_) was set as the approximate P pellet position in the *P+* samples, and a *near‐root* (h_1_) and *far* (h_3_) position were set 2000 µm below and above this position respectively. The effective voxel size was *c*. 1.6 μm. For data collected at I13, only absorption reconstruction was available. For data collected at SLS, both phase/Paganin (Paganin *et al*., 2002) and absorption reconstruction were available. See Methods S1 for full details.

### Synchrotron X‐ray computed tomography image analysis

Unless specified otherwise, image analysis was carried out using custom scripts written in ImageJ/Fiji (Schindelin *et al*., 2012). The soil phase was first segmented into three phases: air‐filled pore‐spaces (‘pore’), primary mineral grains (‘primary’), and mixed phase (‘mixed’) (Methods S1: Figs S1.13–S1.16 therein) using a WEKA machine‐learning approach (Daly *et al*., 2015; Keyes *et al*., 2017; Koebernick *et al*., 2017). Hyphal classification was carried out using the *absorption* volumes due to the greater hypha‐to‐pore contrast (Methods S1: Fig. S1.12 therein), after the soil segmentation result from the *phase* reconstructed volumes had first been used to mask out mineral regions. Hyphal classification was carried out using a custom segmentation approach with morphology first filtered using a two‐pass morphological filter to remove small noise artifacts while conserving long filamentous structures of largely consistent diameter (i.e. hyphae (Methods S1)).

The segmented and filtered hyphal structures were skeletonised, and the following metrics were quantified using the BoneJ toolbox (Doube *et al*., 2010): total hyphal length, number of discrete hyphal clusters, branch count per cluster, mean branch length per cluster, tortuosity of branches, and angle of orientation to the hyphal compartment midline (i.e. the vector normal to the barrier mesh). These metrics were computed in Matlab 16b (Natick, MA, USA), along with the SD and SE in the mean across replicates for each measure (Methods S1).

### Traditional measurements

#### Traditional hyphal counting

The fraction of hyphae captured by the SXRCT approach was compared against that derived from a destructive sampling method (Jakobsen *et al*., 1992a,b). Replicate hyphal compartments were cut into three equal sections at the same locations used for SXRCT imaging, and each soil sample was divided into two subsamples for counting (Methods S1). Hyphae were counted using digitised images collected via light microscopy with an BX41 microscope (Olympus UK & Ireland, Southend‐on‐Sea, UK) with a ×20 objective magnification and bright‐field illumination. A total of 20 images were acquired from randomly selected locations across each sample. This produced a total of 40 images for each soil sample. These images were examined to assess the phenotypic diversity within the entire sample set (Methods S1: Fig. S1.29 therein). Based on these classifications, a set of candidate AMF phenotypes was defined with reference to the literature (Abbott & Robson, 1985; Friese & Allen, 1991; Giovannetti *et al*., 1993, 2001, 2004), to which all hyphal structures were subsequently assigned manually during the counting stage. See Methods S1 for full detailed methods and results.

#### Polymerase chain reaction

Polymerase chain reaction (PCR) analysis of soil double‐stranded DNA purified from soil was carried out to validate the colonisation of hyphal compartments by AMF. Four sets of primers were used: (a) AMF primers (Kruger *et al*., 2009) designed to amplify Glomeromycota fungi, (b) a bespoke set of AMF internal transcribed spacer (ITS) primers (referred as Tkacz primers), (c) a broad range primer (Buee *et al*., 2009a,b) designed to amplify Ascomycota and Basidiomycota rather than Glomeromycota, and (d) bacterial primers (515F and 806R) targeting the prokaryotic 16S rRNA V4 region (Caporaso *et al*., 2011). See Methods S1 for further details of the primers used. We first confirmed that primers (a) and (b) target AMF, including *R*. *irregularis*, while primers (c) and (d) target other fungal and nonfungal species, but not AMF (Methods S1: Section 7.3). For all primer sets, standard polymerase chain reaction (PCR) conditions were used: 98°C for 3 min, 35 cycles of 98°C for 15 s, 55°C for 30 s and 72°C for 100 s, followed by a final elongation step at 72°C for 7 min using Phusion high‐fidelity polymerase (M0530L; New England Biolabs, Hitchin, UK) and a PCR master mix with GC buffer (M0532L; New England Biolabs). Following PCR, electrophoresis gels were used to determine whether amplification had been successful.

#### Microbial diversity analysis

The 18S rRNA genes were amplified from soil DNA extracts from hyphal compartments using the PCR primers F‐574 and R‐962 (Hadziavdic *et al*., 2014), and sequencing was performed on an Illumina MiSeq platform (Environmental Sequencing Facility, University of Southampton). Sequences were processed and analysed using the Dada2 pipeline (Callahan *et al*., 2016). Sequences shorter than 320 bp were discarded. Amplicon sequence variants (ASVs) were classified using the Wang Bayesian classifier in Dada2 and the Silva taxonomy (Pruesse *et al*., 2012). The taxonomy of selected ASVs that could not be identified using the classifier were individually analysed by Blast (Altschul *et al*., 1990) against the nonredundant (nr) NCBI database; the taxonomy of the closest Blast match was used as an indication of the identity of the ASV. The ASV counts were subjected to a Hellinger transformation with the *decostand* function in the vegan (Oksanen *et al*., 2013) software package. Principal component analysis (PCA) was performed using the R function *prcomp*. To focus on the ASVs that best explained the difference between these samples, the 50 ASVs with the largest absolute loadings in the first and second components were selected. A heatmap representation of the relative abundance of these ASVs was constructed using pheatmap (Kolde, 2019). Sequence data were deposited in the NCBI Sequence Read Archive under accession no. PRJNA498673.

#### Root colonization analysis

To independently confirm AMF colonisation, cleared and stained roots were imaged via microscopy for signs of AMF colonisation based on the visible presence of intraradical hyphae, vesicles, arbuscules and external hyphae.

#### Plant biomass

Fresh roots and shoots from the plant compartments were weighed and dried at 70°C for 48 h. Dry samples were digested using a H_2_SO_4_/H_2_O_2_ digestion method (Novozamsky *et al*., 1983) and prepared for high resolution inductively coupled plasma mass spectrometry (HR‐ICP‐MS) analysis to determine the total P content.

#### Chemical mapping of phosphorus and sulphur

The SXRCT‐imaged hyphal compartment replicates were freeze‐dried (to preserve organic residues, e.g. hyphae and bacteria) and fixed in epoxy resin (Epotek‐301 (Epoxy Technology Europe Ltd, Marlborough, UK) diluted with ethanol; Methods S1: Fig. S1.32 therein). The samples were prepared using standard geological thin‐sectioning procedures (Camuti & McGuire, 1999; Lanzirotti *et al*., 2010).

At the DLS I18 beamline hyphal compartment, longitudinal thin‐sections of hyphal compartments were mounted on a three‐axis‐stage. The beamline comprises Kirkpatrick–Baez mirrors producing a spot size of 20 μm, utilising a silicon (Si(111)) monochromator to scan the incident beam energy. Chemical maps were acquired under a helium atmosphere at 3 and 2.7 keV photon energies (to mitigate artifacts from chlorine present in the epoxy resin). For each pixel, a full energy‐dispersive spectrum was recorded using a four‐element Vortex silicon drift detector (Hitachi) positioned normal to the incident beam and 45° relative to the sample. The flux was estimated to be between 10^10^ and 10^11^ photons s^−1^. The same setup was used at SLS, except that elemental maps were obtained under vacuum (10^–6^ mbar) and X‐rays were detected using a single element Ketek silicon drift detector. All maps were fitted using the pymca package in batch fitting mode (Sole *et al*., 2007). Concentrations (in µg g^−1^) of Al, Si, P and S were obtained by fitting maps obtained under identical experimental parameters using a spessartine garnet and Durango apatite mineral standards with known elemental concentrations (Methods S1: Tables S1.2, S1.3 therein).

**Fig. 1:**
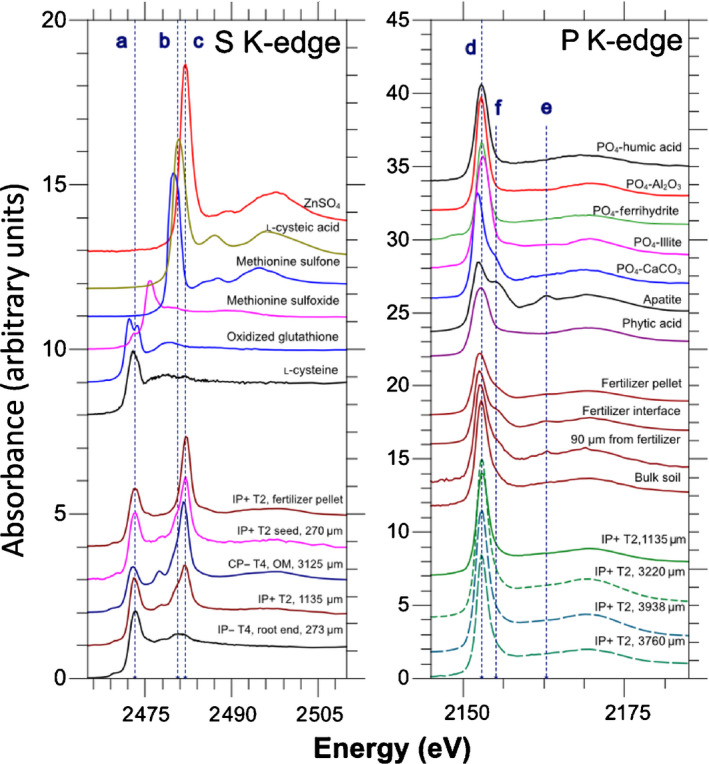
Sulphur (S) and phosphorus (P) K‐edge (X‐ray absorption near edge structure) XANES spectra. The top set of spectra are from standards used for linear combination fits; the bottom set are point measurements as a function of distance from the segmented hyphae. The selection of collected spectra from the samples show spectral differences. For S, the ratios between different peaks are particularly important. The vertical dashed lines represent the energies of l‐cysteine (labelled ‘a’), sulfonate (‘b’), and sulfate (‘c’). Some spectra show contrasts, for example IP– T4 (inoculated, no supplemental P, *t* = 4 wk) at the end of the compartment closest to roots (predominantly cysteine) and IP+ T2 (inoculated, added phosphate, *t* = 2 wk) at the fertilizer pellet location (predominantly sulfate). For P, the spectral differences are much more subtle. However, the differences are measurable. In particular, calcium orthophosphate (CaPO_4_)‐like species have a shifted white line (the sharp feature in the K‐edge spectrum) towards lower energy (labelled ‘d’), a shoulder (‘e’) and oscillation (‘f’) at higher energies. Interestingly, the shoulder for CaPO_4_‐type species disappears with increasing distance from the fertilizer pellet, suggesting dissolution and re‐adsorption to different mineral surfaces. Spectral changes with increasing distance from fungal hyphae do not show significant differences. CP+, control added P; CP–, control no added P; IP+, inoculated added P; IP–, inoculated no added P.

X‐ray absorption near edge structure spectra were collected in  fluorescence mode to constrain changes in P (K‐edge) and S (K‐edge) speciation. The K‐edge positions of P and S were calibrated against the first derivative of plots obtained using apatite (Ca_5_(PO_4_)_3_(OH)) and sulphate (ZnSO_4_) standards. Spectra from a series of P and S standards (Fig. 1) were collected to perform a linear combination fit.

Phosphorus concentration with distance from the root compartment was estimated from the XRF P intensity maps. Average P concentrations and SD were calculated for the mixed‐phase pixels. Since both P and S are in the tender X‐ray region, and the attenuation length of P in quartz is *c*. 2 µm, these maps are assumed to be 2D representations of concentration. Thus, the P, S and Al XRF maps were aligned via visual inspection to the corresponding SXRCT data of the same physical samples to correlate the chemical and structural information. The correlated data were used to determine if the presence of hyphae was correlated with local variation in elemental concentrations of P, S and Al. A 3D Euclidean distance transform of the SXRCT‐derived segmented hyphal geometries for each hyphal compartment was used to determine distance from hyphae to each position on the XRF maps. Pixels on the XRF maps were separated into two distance classes: pixels within 50 µm of the nearest hyphal surface (‘close to hyphae’) and further than 200 µm (‘far from hyphae’). Pixels on the XRF maps were labelled as mixed phase if they were determined to be neither air (low total XRF signal) nor primary mineral (high Si signal). Excluding pixels that indicated high Si also limited the overflow of Si signal into the neighbouring P or Al signal. Mean XRF counts of P, S and Al in the mixed phase were measured for each distance class of pixels over all treatments.

### Mathematical modelling

The AMF hyphae length density data at T_2_ and T_4_ (2 and 4 wk of plant growth) were fitted with a model developed by Schnepf *et al*. (2008a) which included the simplest linear net hyphal branching term (see Methods S2 for a full mathematical modelling description). The model fitted was ∂*_t_* *n* + *v*∂*_x_n* = *bn*, ∂*_t_ρ* = *n*|*v*| − *dρ*, where *n* is the hyphal tip density, *v* is the hyphal tip growth rate, *b* is the net hyphal branching rate, *ρ* is the hyphal length density, *d* is the net hyphal length destruction rate, *t* is the time and *x* is the distance from the root compartment along the hyphal compartment midline. The model was solved with a zero initial condition (i.e. *n* = 0 and *ρ* = 0 at *t* = 0), assuming constant *k* tip production at the root surface (i.e. *v* = *k* on *x* = 0 for *t* > 0). We estimated values using the Matlab R2017a *fmincon* routine for *k*, *d*, *b* and *v* by fitting the model to the length–density data from both SXRCT and destructive (Jakobsen *et al*., 1992b) measurements. This gave us the lower and upper bound for the parameter values, since SXRCT only detects hyphae in the pore spaces and the method described by Jakobsen *et al*. (1992b) detects all hyphae.

X‐ray fluorescence data for P was fitted with the model of Schnepf & Roose (2006), which links the fitted hyphal length density results for *ρ* to the soil P profiles. The model consists of a soil P movement equation that accounts for first‐order binding of P to the soil mineral surfaces and P diffusion in the soil pore‐space, that is, ∂*_t_c*_TOT_ = *D*_eff_∂*_xx_c*_TOT_ − 2*πr_m_λ_h_ρc*_TOT_, where *c*_TOT_ = *c_s_* + *θc_l_* = (*b* + *θ*)*c_l_* is the total amount of P in the soil, *c_l_* is the concentration of P in the soil fluid/mixed phase, *θ* is the volume fraction of the mixed phase, *b* is the first‐order equilibrium binding buffer power of P in soil, *λ_h_* characterizes the rate of P uptake per unit of hyphal surface area per unit volume of total soil P, and *r_m_* is the hyphal radius. The model was solved, setting the boundary condition at the root/AMF hyphae compartment boundary as *D*_eff_∂*_x_c*_TOT_ = *F*_max_ *c*_TOT_ on *x* = 0 and *c_l_* → *c*_∞_ as *x* → ∞, where *c*_∞_ is the P farfield concentration assumed to be the initial condition of P at *t* = 0. We used the value for *D*_eff_ = (*Dθf*/(*θ* + *b*)) = 1.05 × 10^−8^ cm^2^ s^−1^, as estimated by McKay Fletcher *et al*. (2017) and used the fitted results of our hyphal length density measurements *ρ* from the growth model above. We estimated the parameter *λ_h_* against the XRF data by minimizing the sum of squares between the data points and the model using *fmincon* in Matlab R2017a.

### Statistics

Throughout this study, we used the Matlab Statistics Toolbox to analyse data. We used a *t*‐test at a significance of *P* < 0.05, using *ttest2*, for means, and the Kolmogorov–Smirnov (KS) test at *P* < 0.05, using *kstest2*, for the distributions.

## Results and Discussion

### Imaging of hyphal networks

**Fig. 2:**
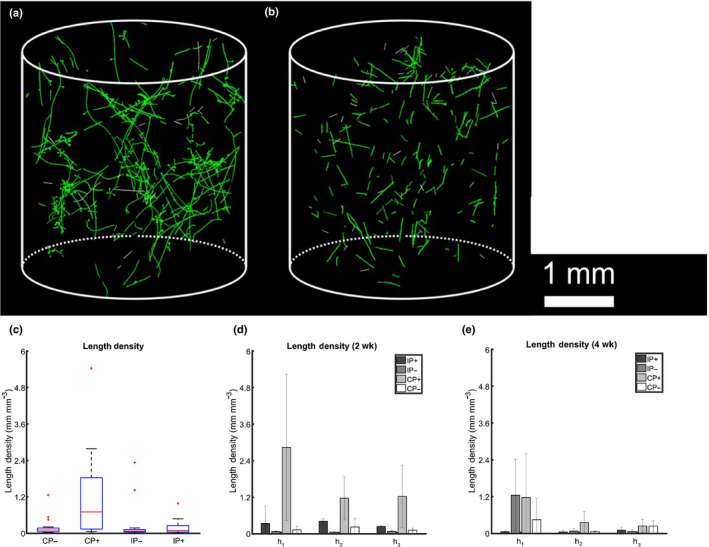
Synchrotron X‐ray computed tomography imaging and image processing protocols enabled extraction of air‐based hyphal morphology. The images in (a) and (b) show sample images of 3D hyphal morphology for exemplary samples from control and inoculated treatments; (c) shows the mean (red line) hyphal length density, the blue box describes the interquartile range (i.e. 25–75% of data falls within this interval), red dots indicate outliers, and black whiskers show ±2.7*σ*, where *σ* is the SD; (d) shows the average hyphal length density for all different treatments at *t* = 2 wk, along with the SD; (e) shows the average hyphal length density for all different treatments at *t* = 4 wk along with the SD. In (d) and (e) h_1_ = 13.5 mm, h_2_ = 28.5 mm, and h_3_ = 43.5 mm from the interface of the hyphal compartment to the adjoining root compartment along the hyphal compartment midline. CP+, control added P; CP–, control no added P; IP+, inoculated added P; IP–, inoculated no added P.

Observations from SXRCT data revealed significant differences in hyphal morphology between treatments (Fig. 2a,b). Hyphae in the control samples are more heterogeneous, have many more branches and have spherical features at some termini, which are assumed to be spores or spore‐like structures. *Rhizophagus irregularis* inoculated samples (Fig. 2b) are characterized by more linear/straight hyphae, less branching and fewer spherical/spore‐like structures.

Synchrotron X‐ray computed tomography imaging is noninvasive and provides richer information than destructive bulk length‐density measurements, enabling estimation of the number of branches per hyphal cluster, branch length, angle and tortuosity. However, as hyphae were only detectable in the pore space due to the contrast limitations of SXRCT, these measurements might not be fully representative of the entire soil hyphal population. Control *P+* measurements showed significantly higher (*t*‐test) and differently distributed (KS‐test) branch numbers per cluster (when defining a single cluster to be all hyphae that are continuously connected on the images) than control *P−* and both *P+* and *P−* inoculated treatments (Fig. 2), suggesting that in the soil used, nonAMF hyphae are more branched in the soil pore‐space than AMF hyphae. The differences in branch number and distribution between inoculated *P+* and *P−* treatments were not found to be statistically significant (*t*‐test and KS‐test) potentially indicating, consistent with previous work (Drew *et al*., 2003), that AMF hyphal morphology is not significantly dependent on P availability. Whilst we cannot fully quantify the nonAMF contribution to the length density, it is logical to conclude that any correction of the length density downwards would correct the hyphal uptake rate upwards. Further integrated studies which go hand in hand with more detailed genomic sequencing would be able to shed light on this.

Synchrotron X‐ray computed tomography data enabled quantification of the alignment of hyphae with respect to the major axis of each hyphal compartment. On average, this hyphal alignment angle varied between 45° and 85°. The only significant difference was that the control *P+* treatment had a lower mean angle than the CP− and inoculated IP+ and IP− treatments (Methods S1: Fig. S1.28 therein, *t*‐test). Further analysis indicated that at the fertilizer pellet location (h_2_), statistically significant differences were recorded in the alignment of hyphae between CP+ (49°) and both *P−* treatments (CP– is 65° and IP– is 67°), and when pooling the data at the same location, hyphae in *P+* treatments were found to be statistically more aligned to the compartment axis (51°) than in *P−* treatments (66°). One way of explaining these differences is in the context of the cost–benefit of the symbiosis: when the supply of P is comparatively more scarce, the hyphae ‘search’ for P by deviating more from the primary growth direction, as defined by the geometry of the growth assay. The SXRCT data also allowed analysis of the (normalised) tortuosity of hyphal branches. Means of tortuosity in the control *P+* and *P−* treatments were not significantly different (*t*‐test), but the distributions were significantly different (KS‐test). This could be explained by the larger variation seen in control *P+* (Fig. 2c). The opposite was found for inoculated treatments, where *P+* displayed a significantly different/wider (normalised) hyphal tortuosity distribution (KS‐test) than *P−*. However, when comparing the inoculated and control *P+* treatments, the inoculated *P+* treatment had a significantly different/wider tortuosity distribution than the control *P+* treatment, supporting the hypothesis that AMF fungi might be searching out P sources more aggressively than the nonmycorrhizal strains present in the control samples.

In the control samples with added P (Fig. 2c, CP+), the overall mean hyphal length density was higher (*t*‐test) and the distribution was different (KS‐test) compared to inoculated samples with plants with and without P addition (*P+/−*) and control samples with no P (*P−*). Since the soil was nonsterile, this suggests that AMF inoculation had a suppressive/allelopathic effect on the development of other soil fungi. Adding localised P also resulted in higher overall hyphal length in both control (CP+) and inoculated (IP+) samples. Differences in the observed hyphal length densities in time as a function of distance (h_1_, h_2_, h_3_) from the mesh interface with the plant compartment can be seen in Fig. 2(d,e). Hyphal length density in this context means the total length of observed hyphae – of any length – per unit volume of soil. Based on these measurements we found that the initial growth of native soil fungi in the *P+* control treatment (Fig. 2d, CP+) was reduced such that the hyphal length density in control (CP+) and inoculated treatments (IP−) was roughly the same by week 4. The inoculated *P+* treatment (IP+) had higher hyphal length density at all three positions at the 2‐wk stage, but by week 4, the inoculated *P−* treatment (IP−) had a higher hyphal length density than IP+ closest to the root compartment (h_1_) (see Methods S1: Section 5.4; Fig. S1.28 therein for supplementary analysis of hyphal branch distance, branch alignment and tortuosity as detected by SXRCT).

The plot shown in Fig. 2c indicates that the *P+* treatments in both control and inoculated treatments led to higher hyphal length density and a different distribution compared to *P−* treatments (*t*‐test and KS‐test) when summing over all positions in the hyphal compartments; this is consistent with the findings of previous studies (Abbott *et al*., 1984; Olsson *et al*., 1997). Mean hyphal length density (*t*‐test) and distribution (KS‐test) for control *P+* was significantly higher/different compared to the inoculated *P+* treatment (Fig. 2c). Similarly, the hyphal length density closest to the root compartment (h_1_, Methods S1: Fig. S1.1 therein) for control samples had a significantly higher mean and a different distribution than inoculated samples at h_2_ and h_3_, but the differences were not found when comparing at the closest (h_1_) distance from the root compartment (Methods S1: Fig. S1.1 therein, *t*‐test and KS‐test). However, the inoculated samples indicated AMF colonisation, whereas hyphae in the control samples were likely from saprotrophic fungal strains. Similar analysis of the results for the Jakobsen measurements revealed an equivalent suppressive/allelopathic effect of inoculum/plant presence (Methods S1: Fig. S1.30 therein). Whilst there are many studies that describe plant root architectural responses to P conditions/heterogeneities in soil, there are few studies that show such results for AMF or other soil fungi (Olsson *et al*., 1997; Cavagnaro *et al*., 2005).

Destructively‐derived hyphal length densities were significantly higher than those determined via SXRCT (Methods S1: Fig. S1.30 therein). This difference can be explained by the majority of hyphae residing in the soil mixed‐phase and/or at the soil‐particle/air interfaces, where they cannot be detected by SXRCT. We conclude that 5% of control and 2% of inoculated sample hyphae were present in the soil pore‐space and 95% of control and 98% of inoculated hyphae were in the inter‐soil aggregate/mixed space. This is to be expected as the soil mixed‐phase has higher P and S bioavailable concentrations and greater water availability. An alternative explanation is that the addition of P as a fertilizer, rather than a substrate with higher naturally‐occurring P, might affect the extent to which hyphae penetrate into the soil mixed phase.

### Polymerase chain reaction, sequencing and other traditional validation measurements

Polymerase chain reaction analysis indicated that one out of three inoculated samples (*P+/−*) tested at the 2‐wk stage were positive with the AMF‐specific Kruger and Tkacz primers. At the 4‐wk stage, all inoculated samples were positive to Kruger primers and 2 out of 3 inoculated samples were positive to Tkacz primers. None of the inoculated samples were positive to broad primers (Buee *et al*., 2009a,b). None of the control samples at weeks 2 or 4 were positive to Kruger, Tkacz, or broad primers, indicating a lack of AMF colonisation as expected.

Amplicon sequencing of eukaryote‐specific 18S rRNA genes verified the presence of AMF in the inoculated samples. One sample (IP+, 4 wk) failed to sequence properly and was excluded. Two ASVs affiliated to AMF were detected in four of the inoculated samples with combined relative abundances of 0.34–1.34%, whereas only one was detected in an uninoculated control sample at a relative abundance of 0.02%; see Methods S1. The reverse PCR primer for amplification had a single mismatch to the gene in *Rhizophagus* species in the penultimate (3′) position, which may explain the low detection rate.

**Fig. 3:**
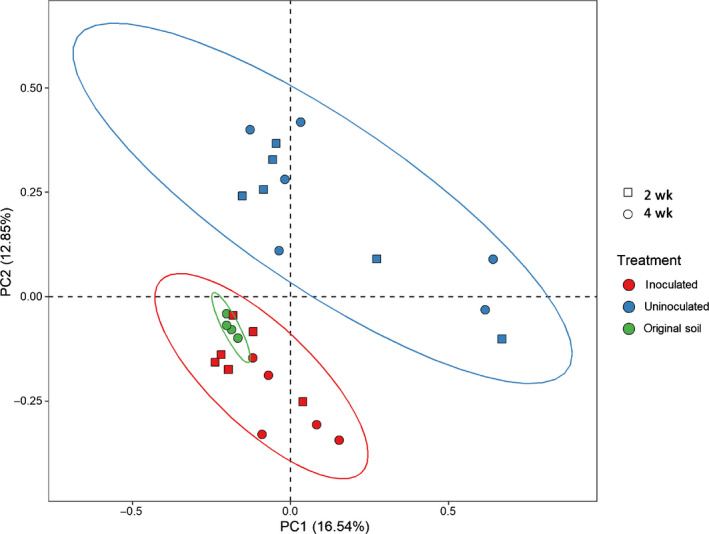
Principal component analysis (PCA) of microbial sequencing results.

**Fig. 4:**
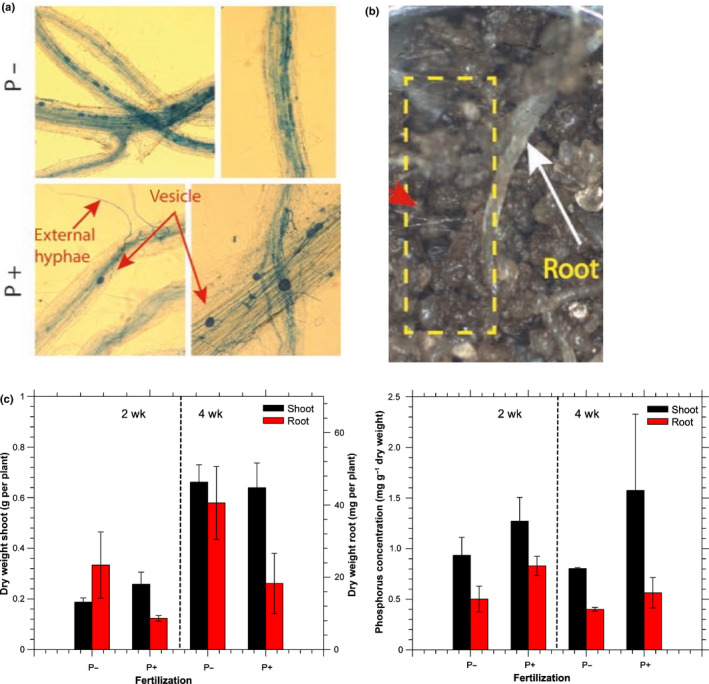
(a) Trypan‐stained mycorrhizal structures (*Rhizosphagus irregulari*s) in wheat roots from root compartments. Staining confirms that plants from all inoculated samples were successfully colonised by mycorrhizal fungi. Red arrows point to mycorrhizas. (b) Microscope image of mycorrhizal structures from a root compartment showing hyphae emerging from a root into the soil at the interface with a hyphal compartment. The red arrow points to mycorrhizas. (c) Root and shoot dry weight and phosphorus (P) content measurements. The significant differences (*t*‐test, *P* < 0.05) are detected for: P+ (added phosphate) vs P− (no supplemental P) root and shoot weights at *t* = 2 wk, shoot and root P content at *t* = 2 wk and *t* = 4 wk for P+ vs P−, and *t* = 4 wk P+ vs P−, and dry root weight.

The total combined relative abundance of sequences assigned to fungi detected in the samples (i.e. Ascomycota, Basidiomycota, and Mucoromycota) was higher in the uninoculated (32.3%) than inoculated treatments (24.7%). Although the sequencing only shows relative and not absolute abundances, when integrated with SXRCT and destructive imaging results, this suggests the growth of native fungi was suppressed by AMF inoculation. The profiles between the inoculated and uninoculated treatments were distinct; see PCA analysis in Fig. 3. These differences were spread across diverse phylogenetic groups of eukaryotes, including nematodes and protozoa. The relative abundance of 18S rRNA genes in the original soil clustered together with the profiles from the hyphal compartments of the inoculated microcosms, implying that the eukaryotic community in the uninoculated hyphal compartments underwent a greater degree of change. On the basis of the genomic analyses, we conclude that the imaged inoculated samples contained AMF hyphae and control samples did not. Moreover, root staining showed clear mycorrhizal colonization (Fig. 4a) in all inoculated treatments.

### Elemental mapping

Synchrotron X‐ray computed tomography images were correlated with elemental maps of P, S and Al for the same samples using XRF allowing hyphae and soil morphology to be correlated with soil chemistry at the pore scale. Previous work has suggested hyphal length density and soil P content should, theoretically, show strong negative correlation (Joner *et al*., 1995; Schnepf *et al*., 2005, 2008a,b, 2011; Schnepf & Roose, 2006; Thonar *et al*., 2011). This study did not find this strong correlation. However, previous studies carried out no chemical mapping or hyphal P‐uptake calibration; hence, the hyphal P uptake parameters were highly uncertain. In this study, simultaneous measurement of hyphal length density and P gradients allowed more accurate estimation of hyphal P uptake rates. Further analysis indicated that organic S is the main S fraction in soil, making up 95–98% of total soil S, due to it being a major component of soil microorganisms, plants and animals (Scherer, 2009). Since the majority of observed soil S can be assumed to be associated with soil microorganisms due to exclusion of animals and roots from hyphal compartments, we treated S as a ‘surrogate marker’ for microbe‐derived soil organic matter. The sequencing analysis enables attribution of this S signal to a wide diversity of prokaryotes (> 2000 ASVs) and microeukaryotes (> 3000 ASVs).

**Fig. 5:**
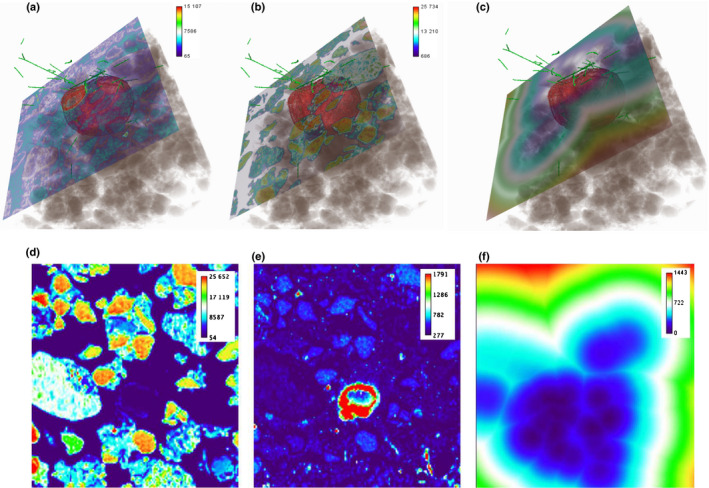
Phosphorus (P), sulphur (S) and distance from hyphae in a 2‐wk time point (T2) inoculation and added phosphate (IP+) treatment shown in their 3D environment. The green tubes are the hyphae segmented from the synchrotron X‐ray computed tomography (SXRCT) data and the brown (red‐greyish opaque) shows organic structure segmented from the SXRCT data. (a) The P heat map correlated to SXRCT. (b) Sulphur heat map correlated to SXRCT. (c) The distance transform of the segmented hyphae correlated to the P and S XRF maps and SXRCT data. The images in (d–f) show the P, S and distance transform from hyphae maps used in the correlation with SXRCT data. (d) The P heat map shows P concentration. (e) The S heat map shows S concentration. (f) The distance from hyphae heat map shows the distance (µm) from the nearest segmented hyphae in 3D. The images show a clear preferential relationship between organic material in the soil and fungal hyphae. The hyphae are pointing towards this structure, which is high in S and relatively low in P. Concentration in (a, b, d, e) is expressed in ppm (mg kg^−1^).

The effect of organic matter on hyphal densities was also observed, and demonstrated how P and S counts were correlated against the hyphal distance maps (Fig. 5a–c). These images show a clear positive correlation between soil organic material and fungal hyphae, in that hyphae are clearly oriented towards a structure which is high in S and relatively low in P. Fig. 5(e) shows relatively uniform S counts across the mixed phase, with the exception of an S‐rich fragment of organic matter which is apparently low in P. An SXRCT‐derived map of 3D distance from hyphal surfaces is shown in Fig. 5(f), indicating high hyphal presence in the S‐rich regions. Hyphal correlation with organic material (Hodge, 2014) was also observed in other samples (Methods S1: Fig. S1.26 therein). It must be noted that since SXRCT does not exclusively detect AMF hyphae, there is a possibility that some of the structures observed may be saprophytic in origin.

**Fig. 6:**
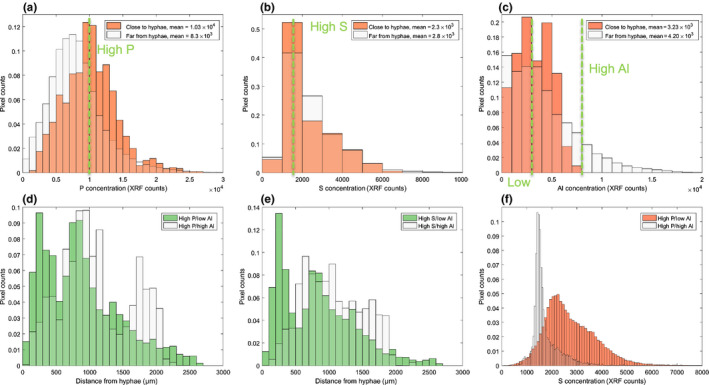
Histograms of X‐ray fluorescence (XRF) phosphorus (P), sulphur (S) and aluminium (Al) concentrations in the clay–water mixture phase over all inoculated samples and planting times, separated by distance from hyphae. (a–c) Phosphorus, S and Al distributions, respectively, of XRF pixels within 50 µm of a hypha (‘Close to hyphae’) and further than 200 µm from a hypha (‘Far from hyphae’). The distance class means, shown in the legend, are significantly different for only P and Al. (d) Histogram of distances from hyphae of XRF pixels with both high P (> 9.7 g kg^−1^)/low Al (25 g kg^−1^), mean = 884 µm, and high P/high Al (> 90 g kg^−1^), mean = 1100 µm. The means are significantly different (*P* ≪ 0.01). (e) Histogram of distances from hyphae of XRF pixels with both high S (> 780 mg kg^−1^)/low Al, mean = 840 µm, and high S/high Al, mean = 1055 µm. The mean values are significantly different (*P* ≪ 0.01). (f) Histogram of S XRF counts with both high P/low Al, mean 980 mg kg^−1^, and high P/high Al, mean 595 mg kg^−1^. The mean values are significantly different (*P* ≪ 0.01). Distances were calculated using the correlated synchrotron X‐ray computed tomography (SXRCT) images. The bin width was selected as 1000 raw XRF units for (a, b, c) and 100 µm for (d, e). The bin width for (f) was 100 raw XRF units for high P/low Al and 50 raw XRF units for high P/high Al. The number of pixels used to create the histograms in (a, b, c) was 1207 for those within 50 µm and 603 410 for those more distant than 200 µm. Each histogram was normalized by the total counts.

Distributions of P, S and Al XRF counts for pixels in the mixed‐phase are shown in Fig. 6(a–c), both close to hyphae (within 50 µm of SXRCT measured hyphae) and far from hyphae (further than 200 µm from SXRCT measured hyphea) over all inoculated treatments. The mean P XRF concentration close to hyphae (1.03 × 10^4^ XRF‐counts) was significantly higher (*P* < 0.05) than far from hyphae (8.3 × 10^3^ XRF‐counts). Fig. 6(a) shows the shapes of the two distributions are similar, but close to the hyphae, the distribution is shifted to the right. A possible explanation is preferential hyphal growth close to areas of higher P concentration. Similar calculations for S showed no significant difference between the means (*t*‐test) or distributions (KS‐test) of the two S distributions (Fig. 6b). The corresponding aluminium (Al) distributions are shown in Fig. 6(c), with the Al mean concentration far from the hyphae being significantly higher than that close to the hyphae (*t*‐test).

The Al concentration close to the hyphae is generally lower and has a narrow distribution of concentration compared to the Al distribution far away from the hyphae (Fig. 6c). This could suggest that AMF hyphae are less likely to grow in proximity to Al‐rich clay minerals, which is consistent with previous findings (Vosátka *et al*., 1999; Göransson *et al*., 2008; Zhang *et al*., 2015). This leads to the following question: do AMF hyphae seek P from Al‐rich clay minerals if they contain high P? To test this hypothesis, XRF pixels were classified into two elemental classes: ‘high P/low Al’, and ‘high P/high Al’, and mean distance from hyphae for these pixels was calculated. *‘*High P’ pixels were defined as having a higher P concentration than the mean concentration of pixels within 50 µm of hyphae (i.e. 1.03 × 10^4^). ‘Low Al’ pixels were defined as having a lower Al concentration than the mean concentration of pixels within 50 µm of hyphae (i.e. 3.23 × 10^3^). The same computation was performed for Al (i.e. 8 × 10^3^; Fig. 6c). The mean distance from hyphae for the ‘high P/low Al*’* pixels was 884 µm, which was significantly lower (*t*‐test; *P* < 0.05) than the ‘high P/high Al’ mean of 1100 µm. The distribution of distance from hyphae for the two elemental classes is shown in Fig. 6(d); ‘High P/low Al’ has many more pixels that are close to hyphae (within 500 µm). Interestingly, only 0.05% of ‘high P/high Al’ pixels are within 100 µm of hyphae, compared to 1.52% of ‘high P/low Al’ pixels. These results suggest that AMF might avoid Al‐rich clay minerals as a source of P. Such a result, if confirmed to be AMF‐specific, could have significant and detrimental implications for the ecological effectiveness of the AMF–plant symbiosis in clay soils. Previous research has also highlighted the importance of soil texture in the role of AMF in N cycling (Zhai *et al*., 2021) and the establishment of different AMF species (Mathimaran *et al*., 2005; Verbruggen *et al*., 2012).

Results for ‘high S/high Al’ and ‘high S/low Al’ are shown in Fig. 6(e), indicating that hyphae are closer to regions of high‐S and low‐Al. This is consistent with the assumption that a high‐S signal could be associated with microbial presence in the soil and that much of the microbial population chooses other sources to mine, for example Fe‐rich minerals (redox active) and organic material. There were no significant differences between the mean concentrations of P, S or Al for pixels (*t*‐test) close to and far from the hyphae measured in the control samples.

When interpreting these results, it should be recalled that SXRCT only detects hyphae in the soil pore‐space, and as set out above, the destructive Jakobsen measurement suggests the majority of the hyphae are in the clay–water mixture phase (see Methods S1: Fig. S1.30 therein). Hence, mixed‐phase pixels classified as far from hyphae may in fact be near to hyphae that were undetected by SXRCT. Nevertheless, it is likely that regions of mixed‐phase surrounded by hyphae in the pore‐space also contain a large number of hyphae (i.e. hyphal presence in pores is a proxy for hyphal presence in proximate mixed phase regions and soil/air interfaces). Pixel distance from hyphae is disregarded in Fig. 6(f), and the S XRF concentration distribution between the ‘high P/high Al’ pixels and ‘high P/low Al’ pixels is shown, demonstrating that the ‘high P/low Al’ pixels have a significantly higher mean S concentration and a larger tail. Under the assumption that S is a surrogate marker for microbial presence, a similar conclusion as to Fig. 6(e) could be made: the microbial community avoids Al and P rich clay minerals. Local biotoxicity and low bioavailability are both possible explanations for this observed behaviour.

### Speciation of phosphorus and sulphur via X‐ray absorption near edge structure

The collected XANES spectra of standard samples and a selection of spectra of the hyphal compartment samples are presented in Fig. 1: (with various features labelled ‘a’–‘f’). For S, thiol peaks (*c*. 2473 eV) and sulfonate (*c*. 2480.5 eV) and sulphate (*c*. 2482 eV) ratios are clearly observable, which suggest differences in organic (cysteine, glutathione, sulfoxide, and sulfone) and inorganic S (sulfate cysteic, and sulfone) pools. For P, these spectral changes are subtle, with the largest changes observed around the fertilizer pellet in the *P+* samples – which showed signs of dissolution – said changes being indicated by a shift in the white line and disappearance of the shoulder after the white line (features labelled ‘d’–‘f’ in Fig. 1), suggesting adsorption of P to soil mineral surfaces. Spectral changes with increasing distance from fungal hyphae did not show significant differences.

**Fig. 7:**
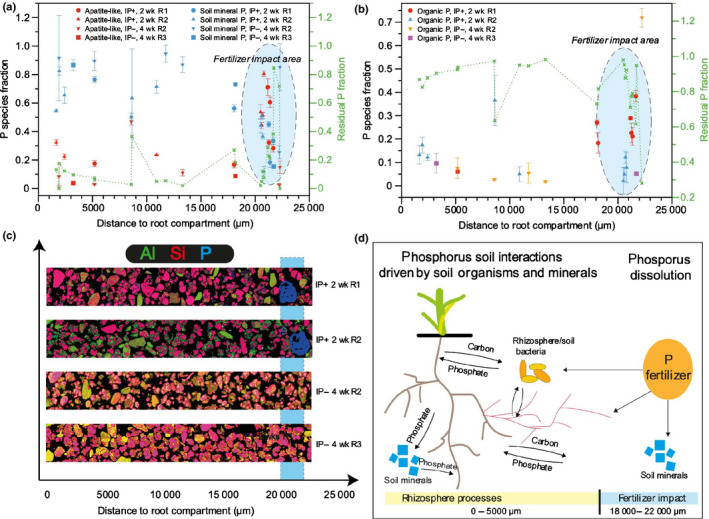
Linear combination fitted X‐ray absorption near edge structure (XANES) results of phosphorus (P) K‐edge with respect to distance from the root compartment, showing (a) apatite‐like and soil mineral P fractions, (b) organic associated phosphate (PO_4_), (c) the collected elemental maps which show how the distances are related, and (d) the postulated P interactions based on the XANES results. The blue highlighted ellipsoids in (a) and (b) and the vertical blue bar in (c) denote the area affected by the triple super phosphate fertilizer. The data show no clear trends for apatite‐like and mineral‐associated P (a). However, the organic‐associated P fraction is increased closer to the roots, as depicted in (d). The units are given in mass fraction form (0–1) with 1 meaning 100%. The residual P fractions of (a) and (b) add up to 1. Error bars represent SD. CP+, control added P; CP–, control no added P; IP+, inoculated added P; IP–, inoculated no added P; R1, replica one; R2, replica two; R3, replica 3.

**Fig. 8:**
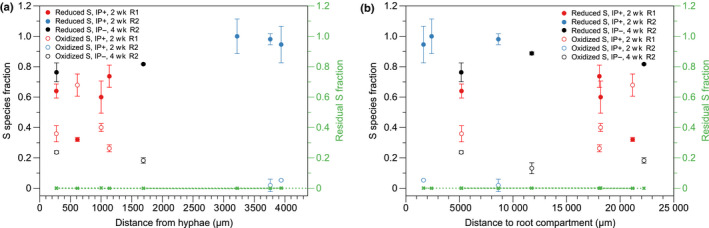
Linear combination fitted X‐ray absorption near edge structure (XANES) results of sulphur (S) K‐edge. (a) Reduced (i.e. amino acid thiols) and oxidized (i.e. sulfonate/sulfate) S species distribution vs distance from segmented hyphae derived from synchrotron X‐ray computed tomography (SXRCT) data. The data show that closer to hyphal surfaces, more amino acid thiols (e.g. cysteine) and fewer oxidized S species (e.g. sulfonate and sulfate) are detected. (b) Reduced and oxidized S species distributions with respect to distance from the interface of the hyphal compartment with the associated root compartment. Here the data show an opposite trend: more oxidized S species closer to the roots and less amino acid thiols. Units are in are mass fraction (0–1) with 1 meaning 100%. Error bars represent SD. IP+, inoculated added P; IP–, inoculated no added P; R1, replica one; R2, replica two.

Linear combination fits of the P and S XANES spectra (Fig. 1) were performed in order to constrain the compositions. The results suggest that there is an increased organic‐associated pool of P and oxidized S species close to the interface with the root compartment (Figs 7c, 8c,d). These results are in accordance with those of a previous study by our group (van Veelen *et al*., 2020). These changes in speciation appear to be the result of an increased microbial community and related biochemical activity in either the rhizosphere or hyphosphere. The increased microbial biomass resulting from C input by roots (i.e. rhizodeposits) or mycorrhizal fungi is known to play an important role in the S cycle (Leustek & Saito, 1999). The rate at which mineralisation of organo‐sulphur in soils takes place is dependent on both the proportions of sulphate‐esters and C‐bonded S, and by the type of crop (Bertin *et al*., 2003). Previous studies by Vong (2003) found that addition of glucose or other C compounds, such as organic acids, to soil encourages rapid bacterial growth which transforms inorganic S into organic S. Similarly, significant organic S increase has been observed when cellulose was added as a C source (Chowdhury *et al*., 2000). An increase in microbial biomass and organic matter will also increase the concentration of organic‐associated P. There is also evidence that soil microbes actively compete with plants for available sulphate in the rhizosphere (Kertesz & Mirleau, 2004).

The presence of AMF caused an opposite trend in S speciation, with increased oxidized sulphur closer to hyphae (Fig. 8a,b). These changes were not observed in the controls (Methods S1: Fig. S1.37 therein), which indicates that the observed changes are microbiome related. The S composition of the organic structure presented in Fig. 8 was determined to comprise amino acid thiols (64.1%), sulfonate (12%) and sulphate (23.9%). This suggests that the hyphae, together with bacteria, preferentially mine organic S (amino acids and sulphate esters). In addition, this suggests that AMF hyphae are actively involved in the conversion and transport of S to the plant roots (Allen & Shachar‐Hill, 2009). This is another indication that S could be used as a surrogate marker for hyphal abundance, providing a proxy for protein, which is abundant within hyphae (Rak *et al*., 2014). Finally, P speciation in both controls (Methods S1: Fig. S1.36 therein) and inoculated treatments (Methods S1: Fig. S1.34 therein) did not reveal clear trends with increasing distance from hyphae. Such a lack of clear changes in P speciation around hyphae might have been caused by their preferential mining of P from organic material which has a low overall P concentration. In addition, the competition and mutualism between microbes and AMF also influence the speciation of P in the soil microbiome. Finally, the different dynamics (i.e. competition between organisms, and mineral mining), as shown in Fig. 6, will likely affect P speciation.

The addition of a fertilizer pellet had an overall effect on the speciation of both P and S in the surrounding soil via dissolution and diffusion (Fig. 7). However, these effects were limited to the area directly adjacent to the pellet due to the absence of a significant mass flow of water and low P diffusion in soil. Consequently, it did not influence P speciation closer to the root compartment.

### Modelling results

The results of the hyphal growth model fitted to the traditional destructive and SRCT hyphal measurements (Methods S2) are shown in Table 1, together with a previous estimation of the same parameters using a different dataset by Schnepf *et al*. (2008a). We observed that the hyphal elongation rate *v* ≈ 10^−5^ cm s^−1^ estimated using the Jakobsen and SXRCT data are similar, as the pattern of soil colonisation is similar, but both are one order of magnitude faster than was estimated by Schnepf *et al*. (2008a). The root hyphal infection rate estimates based on our two methods of hyphal observation for *k* ≈ 10^−4^ cm^2^ s^−1^ are an order of magnitude lower than estimated by Schnepf *et al*. (2008a). These differences between our hyphal parameter estimates and those estimated by Schnepf *et al*. (2008a) might be due to the fact that the previous estimates were based on experimental measurements (Jakobsen *et al*., 1992a,b) gathered using a much larger‐scale assay, and whilst the fungal inoculum was the same, the plant used in past studies (Jakobsen *et al*., 1992a,b) was not a wheat plant, but clover (*Trifolium subterraneum*). Furthermore, a different soil was used between the two experiments, and this would be expected to lead to different AMF colonisation dynamics.

**Table 1:** Estimated best‐fit model parameters for hyphal length density measured using traditional Jakobsen destructive soil sample testing (first column) and synchrotron X‐ray computed tomography (SXRCT) data (middle column), in comparison to the values estimated in Schnepf *et al*. (2008) (last column).

	Model fitting parameters for hyphal length densities obtained by Jakobsen destructive testing method	Model fitting parameters for hyphal length densities obtained by SRXCT analysis method	Values from Schnepf *et al*. (2008a)
*k* (cm^−2^ s^−1^)	4.66 × 10^–4^ ± 1.758 × 10^–4^	1.02 × 10^–4^ ± 1.767 × 10^–4^	2.89 × 10^–3^
*b* (s^−1^)	2.07 × 10^–6^ ± 1.386 × 10^–6^	5.45 × 10^–6^ ± 1.273 × 10^–5^	5.79 × 10^–9^
*v* (cm s^−1^)	1.64 × 10^–5^ ± 9.047 × 10^–6^	2.06 × 10^–5^ ± 2.566 × 10^–5^	2.89 × 10^–6^
*R* ^2^	9.89 × 10^–1^ ± 7.680 × 10^–3^	2.34 × 10^–1^ ± 2.53 × 10^–1^	–

Numbers show mean ± SD. *k*, root hyphae infection rate; *b*, net hyphal branching rate; *v*, hyphae elongation rate; *R* ^2^, goodness of fit measure.

**Fig. 9:**
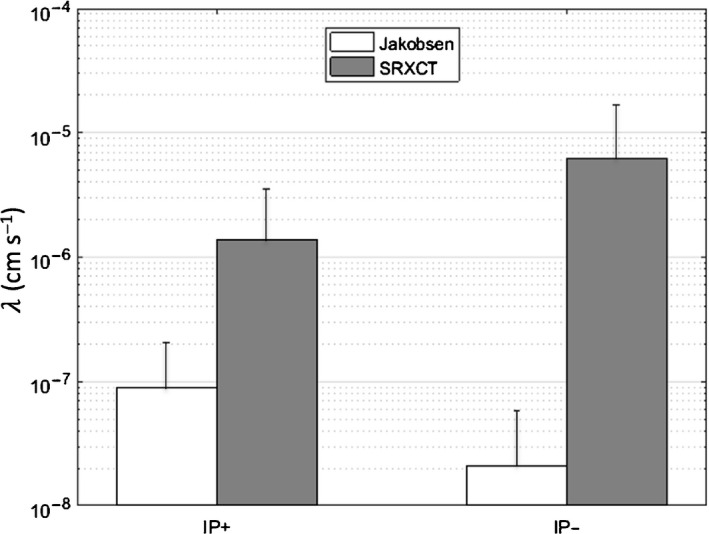
Model‐fitted hyphal phosphorus (P) uptake rate *λ* for the inoculated samples with (IP+) and without (IP−) fertiliser treatments using both the synchrotron X‐ray computed tomography (SRXCT) and destructively measured hyphal parameters. Error bars represent the SD.

When the SXRCT‐based hyphal growth model was used to estimate the hyphal P uptake rate *λ*, the values ranged between 10^–11^ and 1.86 × 10^–5^ cm s^−1^, with a mean of 3.78 × 10^–6^ cm s^−1^ (Fig. 9), which is in agreement with the value 3.26 × 10^–6^ cm s^−1^ estimated by Schnepf & Roose (2006). Estimates of *λ* based on data from destructive hyphal analysis ranged from 4.5 × 10^–14^ to 2.19 × 10^–7^ cm s^−1^, with a mean of 1.05 × 10^–7^ cm s^−1^. These are much lower than both the SXRCT estimate and that of Schnepf & Roose (2006). The destructive length density measure is probably an overestimation of the true AMF hyphal length density, as it contains nonAMF fungi, resulting in an underestimation of the true hyphal P uptake rate.

### Conclusions

In this study we showed for the first time that AMF networks can be visualised in 3D within soil using SXRCT, albeit as yet only in the soil pore‐spaces. We found that fungal structures and morphology respond to local P concentrations (Cavagnaro *et al*., 2005) (i.e. *P+* treatments had many more hyphal structures than *P−* treatments). Whilst it has previously been shown that root architectures respond to soil conditions in this manner (Shen *et al*., 2011), this is possibly one of the first studies to visualise *in situ* that AMF hyphae respond similarly to differences in soil P conditions.

The modelling results unequivocally show that hyphal P uptake rates are an order of magnitude lower than previously thought (Schnepf & Roose, 2006; Schnepf *et al*., 2008a,b, 2011) and an increase in hyphal length density does not lead to a significant decrease in soil P content. Correlation of SXRCT and XRF/XANES data suggested colocation of hyphal length density and P and S species in the clay/water soil phase, and suggested S is a candidate proxy marker for hyphae in this soil phase. Our results also hint that AMF hyphae might preferentially mine P from organic structures such as organic‐matter fragments, and confirm the hypothesis of microbial–fungal mutualism. As such, this would suggest that AMF fungi (or organisms that they support in the hyphosphere) acquire nutrients from more reactive labile and/or organic sources over clay minerals, even when soil is high in P, supporting our observation of preferential P acquisition.

Finally, whilst many challenges remain, our study provides strong motivation for interdisciplinary groups to apply fully‐integrated experimental and modelling approaches to the characterisation of plant–soil–fungi systems. Our study represents a necessary step forward in the integration of different techniques to unravel the processes underpinning plant–AMF interaction across different spatiotemporal scales (Ferlian *et al*., 2018), demonstrating that closer integration of theoretical and experimental techniques is important in gaining deeper insights.

## Author contributions

TR designed the study and methodology, and led the data analysis and interpretation. SK led the X‐ray computed tomography measurements and image analysis. AvV led the X‐ray fluorescence measurement and image analysis. DMF, NK, CP, CS, KW, SR and LC worked on and assisted with XCT, XRF, data analysis and modelling. RM, SD and DMF did model fitting to the data. MD led the sequencing analysis. IJ and DJ assisted in data analysis and interpretation. GO assisted in molecular biology and data interpretation. AT and PP conducted the polymerase chain reaction. FM, CB and TH assisted with XRF measurements. All authors co‐wrote the manuscript and Supporting Information. SK and AvV contributed equally to this work.

## Supporting information


**Methods S1** Full description of experimental methods.

**Methods S2** Full description of mathematical modelling.
Please note: Wiley Blackwell are not responsible for the content or functionality of any Supporting Information supplied by the authors. Any queries (other than missing material) should be directed to the *New Phytologist* Central Office.

## Data Availability

Data related to this study are available at 10.5258/SOTON/D2088.
