# Supplementary material for: Multimodal correlative imaging and modelling of phosphorus uptake from soil by hyphae of mycorrhizal fungi
Source: New Phytol. 2022 Feb 15;234(2):688–703. doi: 10.1111/nph.17980 (PMC9307049; doi:10.1111/nph.17980)
Supplement: Supplementary file 1 — Methods S1 Full description of experimental methods. [file NPH-234-688-s002.pdf]

***Supporting Information Methods 1 (SI-1): Full Description of Experimental Methods***

Article Title: Multimodal correlative imaging and modelling of phosphorus uptake from soil by hyphae of mycorrhizal fungi

Authors: Sam Keyes, Arjen van Veelen, Dan McKay Fletcher, Callum Scotson, Nico Koebernick, Chiara Petroselli, Katherine Williams, Siul Ruiz, Laura Cooper, Robbie Mayon, Simon Duncan, Marc Dumont, Iver Jakobsen, Giles Oldroyd, Andrzej Tkacz, Philip Poole, Fred Mosselmans, Camelia Borca, Thomas Huthwelker, David L. Jones, Tiina Roose

Article Acceptance Date: Jan 4th, 2022

# 1. *Plant and fungal growth*

## 1.1. *Sample preparation*

A chamber system was designed to produce millimetre-scale soil samples which were conducive to colonisation by plant-associated mycorrhizal hyphae (Schweiger and Jakobsen 2000, Drew, Murray et al. 2003). The design constraints of this system were as follows:

- Maintain mycorrhizal wheat plants under controlled growth conditions for up to 4 weeks.
- Hyphal compartments must have small enough cross section (<5 mm) to permit adequate transmission of X-rays at ~ 18-20 kV accelerating potential. This is the energy window of highest flux at Diamond I13 and SLS TOMCAT synchrotron beamlines, and allows the most rapid acquisition of high quality synchrotron X-ray computed tomography (SRXCT) data, without photon starvation issues leading to deleterious artifacts. Higher energies are attainable at these beamlines, but flux drops off rapidly with increased energy, leading to longer acquisition times (i.e. in tens of minutes) which leads to unacceptable motion artifacts during acquisition.
- Maintain separation of roots from hyphal compartments to prevent root ingress whilst permitting transfer of hyphae between root and hyphal compartments.
- Maintain hydration at controlled water potentials across root and hyphal compartments during the experiment.
- Permit transport by car and aeroplane for SXRCT imaging at national synchrotron facilities without damage or undue disturbance.
- Permit detaching of un-disturbed hyphal compartments for SXRCT imaging.

A custom growth box was designed to maintain the hyphal and root compartments in dark conditions under stable and externally applied water potential (Figure S1.1). The box was vented to allow gas exchange, with apertures for aerial plant growth external to the box. Water racks established an independently controllable soil water potential for hyphal and root compartments. Stable water potentials were thus established at the base of each hyphal compartment, and the base of each plant chamber (actual values given below).

The growth box was designed in SolidWorks and manufactured from a combination of 3 mm acrylic sheet (Plastock, High Wycombe, UK) and 3D printed ABS parts. Laser-cut parts were cut from acrylic sheet with an 80 kW $CO_2$ laser cutter (LS6090, HPC Laser Ltd,

Halifax, UK). 3D printed parts were manufactured in ABS plastic using an UP! BOX printer (Tiertime, Beijing, China).

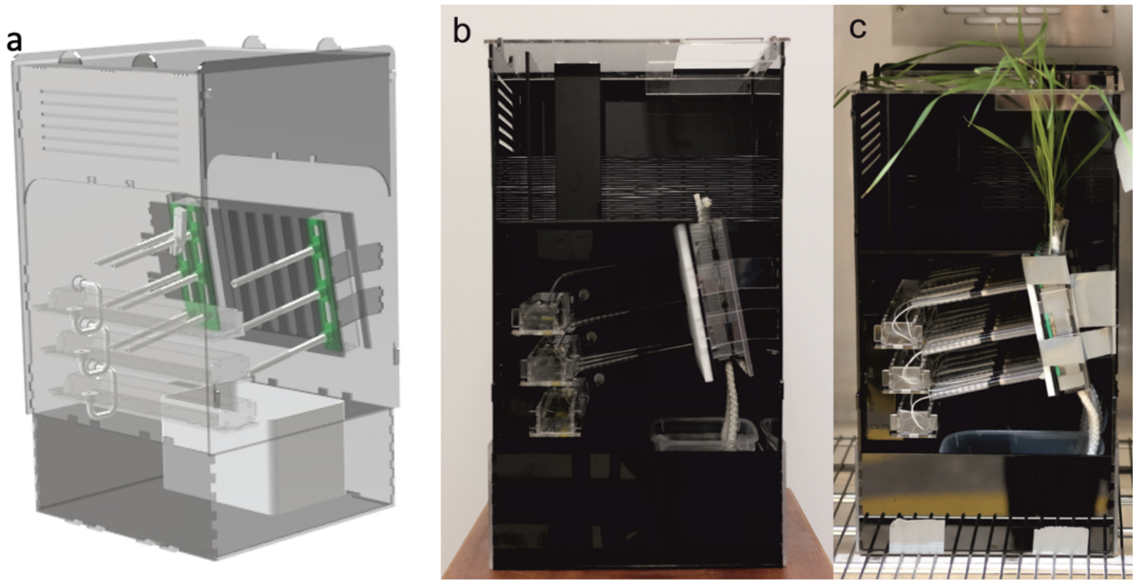


*Figure S1.1 – a CAD rendering of the designed growth chamber (a), the final fabricated version (b), a single chamber immediately before the SRXCT imaging experiment (c).*

The growth medium was a sand-textured Eutric Cambisol soil collected from a surface plot at Abergwyngregyn, North Wales, UK (53°14′N, 4°01′W), for which the soil organic matter content was 7%. This corresponds to soil B in (Lucas and Jones 2006). The soil contained 52.7% sand, 32.8% silt and 14.5% clay. The soil was first sieved using British Standard soil sieves to pass 5 mm, and air dried at 23±1 °C for 3 days. The dried soil was sieved to three fractions: *very fine,* passing through <600 µm; *fine,* between bounds of 1180 and 600 µm; and *coarse*, passing between 1180 µm and 2 mm.

Root compartments were constructed from box-section acrylic tubing of inner diameter 15.9 mm and outer diameter 19.1 mm (Figure S1.2). These were sealed at the base using laser-cut acrylic tabs of 3 mm thickness, each comprising a hole to allow entry of a glass-fibre wick (described below) used to maintain soil water status. Holes of 5 mm diameter were drilled in the front face of each plant chamber at positions at 25 mm, 65 mm and 105 mm respectively from the upper opening of the plant root compartment (referred to herein as d1, d2 and d3). A rectangle of nylon mesh (Normesh, Lancaster, UK) with a 38 µm aperture size was stretched gently over the front face of each root compartment and affixed using a strip of adhesive tape down each side. A braided glass-fibre wick (Stovax Ltd., Exeter, UK) of 5 mm diameter was introduced through the base of each root compartment, running up the entire height. ~20 mm of wick extended above the soil surface, and the wick was held in place by a thin strip of adhesive tape which looped round the wick and held it firmly to the back face of the plant chamber. A ~ 200 mm of free length of glass fibre wick extending vertically below the root compartment was facilitated hydration of each root compartment via capillary rise (Figure S1.2). A Perspex container of water in the base of each compartment acted as the water reservoir. The height between the free water surface and the root compartment was set to provide a water potential ranging between -2 to -3.21 kPa between the base and the top of the root compartment.

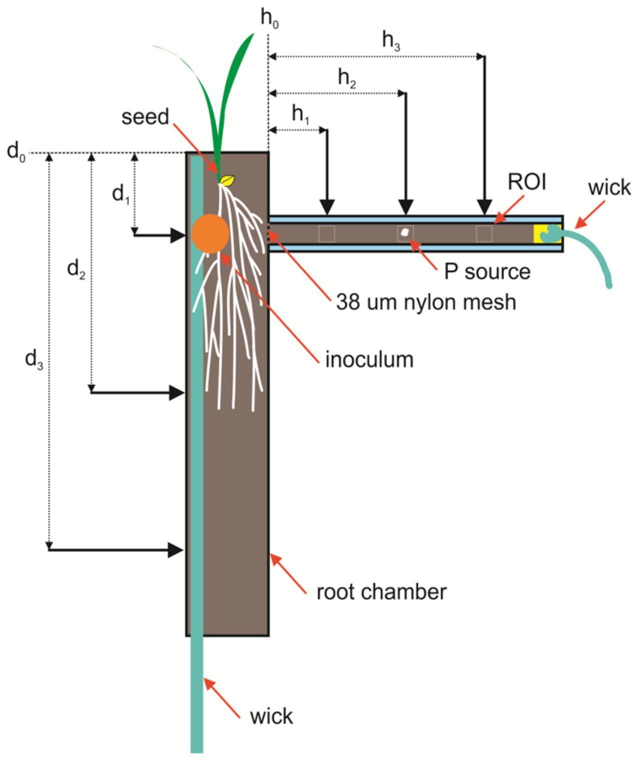


*Figure S1.2 – Schematic of the root and hyphal compartment assembly (showing side-arm hyphal compartment in $d_1$ position only). d is the height of the side-arm centroid from the top of the root compartment, h is the distance in the hyphal compartment, away from the barrier mesh.*

Hyphal compartments were sterile, 1 ml polypropylene syringes (BD Plastipak), cut down to the 1 ml gradation using a scalpel, giving a working volume of 1 ml in each chamber. A braided glass-fibre wick of 1 mm diameter (The Mesh Company, Warrington, UK) was introduced to the base and knotted, with the excess trimmed flush (Figure S1.3). The knot was seated down in the base of the syringe by gently pulling the free end.

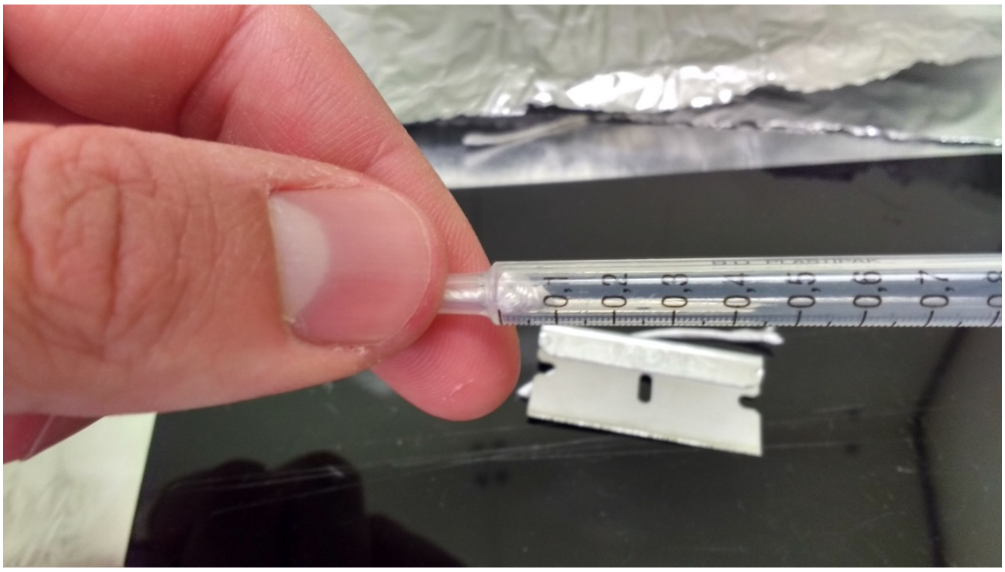


*Figure S1.3 – The glass-fibre wick installed in the base of a hyphal compartment*

The knotted wick end was surrounded by fine sand (British Standard Fraction D) to improve the hydraulic conductivity between the wick and the soil. Oven treated sand (sterilised by heating to 130°C for 24 h in a Lenton oven) was introduced up to the 0.15 ml gradation in each hyphal compartment, and compacted down to the 0.1 ml gradation using

a syringe plunger. Three hyphal compartments (at the $d_1$, $d_2$ and $d_3$ positions) were press-fitted into each 3D-printed hyphal compartment rack.

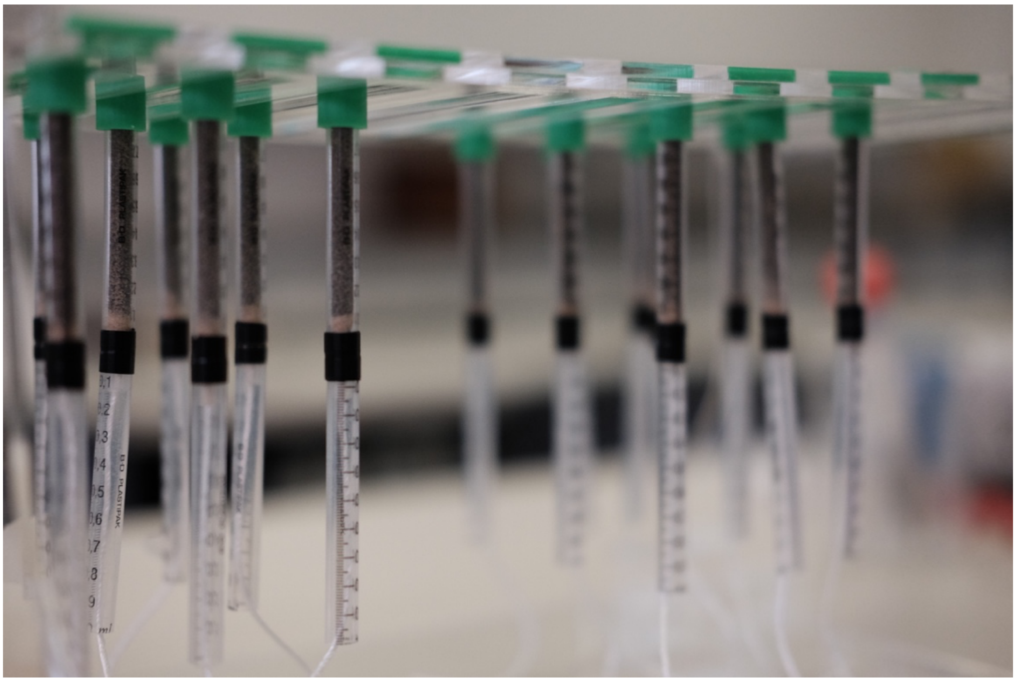

*Figure S1.4 – Completed and soil-filled hyphal compartments installed in holders, ready to be mounted to plant chambers. Sand layer surrounding the knotted end of the glass-fibre wick is visible in the bottom-most ~0.1 ml of the chamber volume.*

The carrier tubes holding the glass-fibre wicks were constructed of sterile 1 ml syringes as before, cut with a scalpel at the 1 ml and 0 ml gradations to form a tube open at both ends. A single tube was fitted over the free end of each wick and fastened to the base of the hyphal compartment with adhesive tape. Soil was introduced up to the ~0.6 ml gradation in each tube, and the tubes tapped until soil settled back to the ~ 0.55 ml gradation (Gregory, Hutchison et al. 2003). In treatments with added phosphate (P+), a small pellet of triple super-phosphate (TSP) fertiliser (mean mass 0.0757 g, SD = 0.007 g) was added at this point using nylon forceps. The tubes were then filled with soil up to the 1 ml mark, tapped to consolidate, and the vacant space re-filled up to 1 ml mark. Each hyphal compartment rack (Figure S1.4) was affixed to the plant root compartment using four strips of adhesive tape, wrapped round the entire circumference of the chamber.

Plant chambers were filled with the *coarse* soil stock (the < 2mm fraction), with differences in chamber filling method between the control and the two inoculated conditions being outlined below.

## 1.2 Non-mycorrhizal controls

Natural, un-sterilised, soil contains naturally occurring fungal spores (including those from mycorrhizal fungi). The decision was taken that due to the difficulty of attaining and maintaining full sterilisation, and the increased likelihood of opportunistic, non-mycorrhizal fungi rapidly dominating colonization of a sterilised soil (as often observed following exposure of sterilised soil) to use unsterilized soil throughout. Since the soil was non-sterile, and assumed to contain mycorrhizal spores, it was necessary to keep the non-mycorrhizal controls plant un-associated, avoiding symbiosis and development of mycorrhizal mycelium. For this reason, the controls were unplanted. The hyphal compartments for the control replicates were filled as outlined above, and were lightly sealed with rubber bungs and maintained at the same water potential as the plant-

associated samples. Controls were prepared for both phosphorus conditions outlined above (P+, P-) at two time points (2 and 4 weeks).

### 1.3 Inoculation with Rhizophagus Irregularis

For inoculation of samples with *Rhizophagus irregularis*, a single-strain inoculum was used (BEG72, PlantWorks Ltd., UK). The inoculum contains hyphae, spores and colonised root fragments at a mean propagule number (MPN) value of 1.6 x $10^6$ propagules.$L^{-1}$, in an inert carrier media of zeolite granules sieved between upper and lower bounds of 3 and 1 mm respectively. To produce a uniform inoculating mixture, the media was concentrated in the following manner, according to manufacturer recommendations. For each planting date (i.e. $T_2$ and $T_4$), a 300 ml volume of as-supplied inoculum was placed in a 750 µm sieve stacked over a 53 µm sieve, and vigorously washed under a water jet for 2 minutes. The material remaining in the 53 µm sieve was gently decanted into a 10 cm culture dish, and *very fine* fraction soil stock (<600 µm) added along with MilliQ water to produce 30 g of media with a stiff paste consistency. During filling of each root compartment, 1.5 g of inoculating paste and 0.5 g of raw inoculum was added via spatula at each of the depths where the hyphal compartments were mounted ($d_1$, $d_2$ and $d_3$). The chambers were tapped gently during filling to consolidate the soil.

### 1.4 Inoculation with Gigaspora rosea

For inoculation of samples with *Gigaspora rosea,* 50 ml of single strain inoculum was provided from the Paszkowski group, Department of Plant Sciences, Cambridge, UK. This was provided in the form of spores suspended in DI water, and was introduced to the root compartments via pipetting. A 5 ml pipette tip was used, with the tip cut down to increase the aperture size (preventing clogging of the tip by suspended plant material). After filling each root compartment with soil, and prior to attaching the nylon mesh, 1.65 ml of inoculum (containing ~330 spores) was gently pipetted into each of the apertures at $d_1$, $d_2$ and $d_3$. Following this, the nylon mesh was immediately attached to the root compartment, and the hyphal compartment carrier was affixed. NB - this variety did not grow much in our assay and we discounted the results. However, we wanted to record here that we tried inoculation with a slower growing mycorrhiza to compare to *Rhizophagus* which is considered a fast growing mycorrhiza.

### 1.5 Plant material

Plant material was a wheat cultivar of high mycorrhizal affinity (*Triticum aestivum* cv. Apache, WBCDB0003-PG-1), provided by the Germplasm Resources Unit of the John Innes Centre, Norwich, UK. Seeds were sterilised in Milli-Q water containing 2.5% sodium hypochlorite (50% b.v. Domestos) and a single drop of Triton X100 surfactant solution. Batches of 24 seeds were gently shaken in 50 ml of sterilising solution for 8 minutes, followed by five rinses in 50 ml of DI water. Germination was carried out in dark conditions on moist Millipore filter paper in foil-wrapped 10 cm culture dishes, with a 96 h germination time at 23±1 °C.

Following germination, the six most uniform seedlings were selected for planting to the root compartments. Using forceps, the seeds were gently planted to the top of each root compartment, 5 mm above $d_1$, and covered with *fine* soil stock to a depth of 10 mm. 10 ml of MilliQ water was pipetted onto the top of each chamber, and a single layer of polythene film used to cover the top of the root compartments, preventing evaporation during the early stages of growth. The film was removed once >15 mm of cotyledon was protruding above the soil surface.

## 1.6 Growth conditions

Each plant assembly (consisting of a single root compartment, with three hyphal sidearm chambers held in the 3D printed carrier) was introduced to the growth box. Each growth box housed 9 of these plant assemblies (triple-replicated samples $R_1$, $R_2$ and $R_3$ at $T_2$, $T_3$ and $T_4$), with three replicates for each single combination of phosphorus condition, time and inoculating strain. Water was added to the hydrating racks in the growth boxes, maintaining a 35±5 mm head (at a total wick length of ~90 mm) at the end of each hyphal compartment. Hyphal compartments were angled downwards at 10° to horizontal. A 80±10 mm head was maintained at the base of each plant chamber, such that the water potential ($\psi_p$) in the root compartment varied from -0.78±0.1 kPa to -1.96±0.1 kPa between the base and the soil surface.

The growth boxes were kept in a controlled growth chamber (Conviron A1000) on an inverse diurnal cycle, with a light period of 14 h at 23°C and 75% humidity under full light (700 µmol.m$^{-2}$.s$^{-1}$), and a dark period of 10 h at 18°C and 75% humidity.
For each phosphorus condition, sets of three biological replicates ($R_1$, $R_2$ and $R_3$) were grown to 2, 3 and 4 weeks after transplanting ($T_2$, $T_3$ and $T_4$), a total of 9 plants per inoculating strain per phosphorus condition.

Imaging was carried out at two synchrotron micro-tomography (SRXCT) beamlines: I13 at the Diamond Light Source (Harwell, UK) and TOMCAT at the Swiss Light Source (Villigen, Switzerland). The differences in these imaging facilities are outlined below, along with which samples were imaged at each beamline. To prepare hyphal compartments (*i.e.* the syringes of soil containing hyphae) for imaging, compartments were carefully removed from the 3D-printed holders, and the wicks cut flush with the base of each compartment. A small piece of paraffin wax was used to seal the compartment base, and a layer of parafilm was used to prevent evaporative losses from the large opening at the top (i.e. the end which had been against the nylon mesh).

At sampling, the hyphal compartments were all weighed to allow estimation of their water content (Table S1.1). The mean water content of the soil in the hyphal compartments was 16% b.v. (SD = 1.7 %).

| **Planting condition** | **Phosphorus** | **Chamber mass (g)** | **SD (g)** |
|---|---|---|---|
| Planted | + | 2.20 | 0.03 |
| Planted | - | 2.17 | 0.06 |
| Control | + | 2.18 | 0.018 |
| Control | - | 2.21 | 0.045 |

*Table S1.1 - The total hyphal compartment mass at the conclusion of the growth experiment*

The dried mass and maximal length of the above-ground plant biomass were measured:

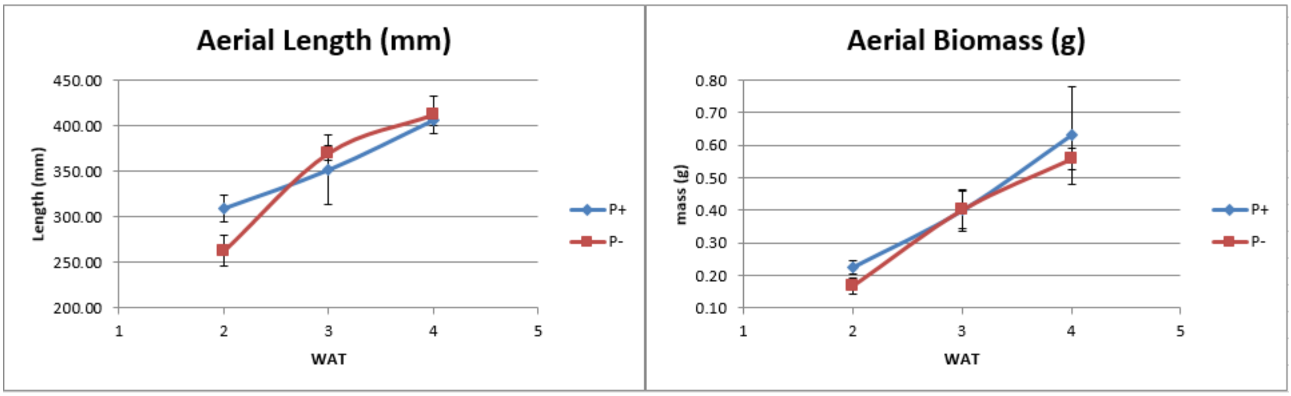


*Figure S1.5 - Plots of maximum aerial length and dry biomass for plant samples. WAT= weeks after transplanting. Error bars show STD around the mean.*

## *1.7 SXRCT Imaging*

The samples were imaged using SXRCT due to the very rapid acquisition times compared to benchtop sources (thus minimising motion artifacts), the high throughput (thus permitting imaging of a large number of samples), and the access to propagation phase contrast due to the coherence of the X-ray source. Two separate SXRCT systems were used: I13 at the Diamond Light Source, Harwell, Oxford; and TOMCAT at the Swiss Light Source, Villigen, Switzerland. The imaging conditions and hardware differed slightly between these two facilities, and are described separately. The beamtime allocation only permitted sufficient replicates of two time points to be measured, so $T_2$ and $T_4$ samples were imaged using SXRCT and $T_3$ samples were neglected due to time constraints.

*Gigaspora* replicates were all imaged at DLS I13, and due to the unavailability of a Paganin reconstruction pipeline, only absorption reconstructions were collected.

All *Rhizophagus* replicates and controls (P+ and P-) were imaged at SLS TOMCAT, and Paganin and Absorption reconstructions were carried out.

Full further sets of controls (uninoculated P+ and P-) were imaged at both DLS and SLS beamlines at a later point.

### 1.7.1 I13, Diamond Light Source

Samples were mounted for imaging in a specially-designed sample stage (Keyes, Boardman et al. 2013, Daly, Mooney et al. 2015, Keyes, Cooper et al. 2017). Samples were aligned down the beam axis using custom knife-edges to allow physical registration of samples with the SXRCT coordinate system during later resin embedding and sectioning protocols. Knife edges were oriented along the beam axis, and all samples aligned with the knife edges before imaging, using the line of volumetric gradations on the side of each syringe as a datum (Figure S1.6).

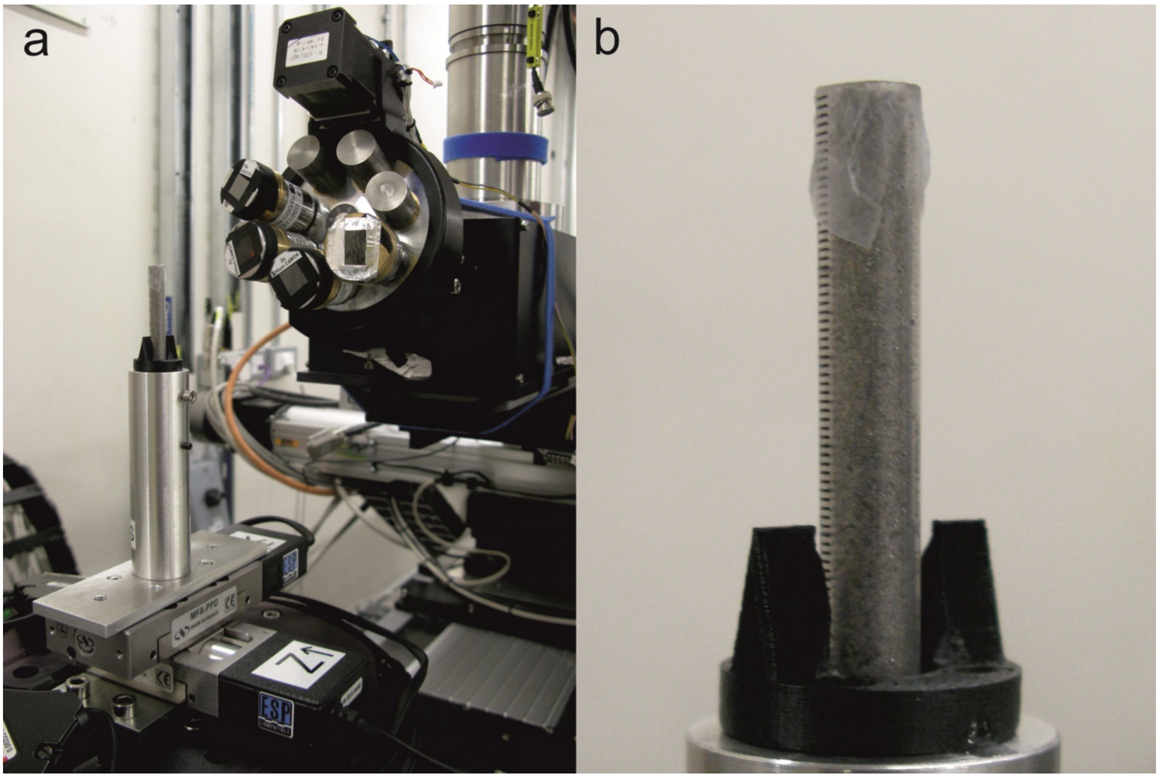


*Figure S1.6 – Sample mounted for imaging at I13 (a). A close-up view shows the alignment of the gradation line of the sample with 3D-printed knife edges that have been aligned down the beam axis using a laser.*

For each hyphal compartment, three vertical positions were chosen for imaging. The *intermediate* position ($h_2$) was set as the 0.5 ml gradation, and the *near-root* ($h_1$) and *far* ($h_3$) positions were set 2000 µm below and above this central line.
Samples were imaged under monochromatic beam conditions, using an accelerating potential (i.e. energy) of 18 kV, and a filter comprising 250 µm of Al placed between the monochromator and the sample to reduce thermal load on the sample. The transmitted X-ray signal was scintillated by a CdWO4 film of 100 µm thickness, and optically magnified by a factor of 4x. The resulting signal was collected using a PCO Edge 5.5 camera. A total of 1600 projections was acquired for each scan using a 150 ms exposure. A 50 mm sample-to-detector distance was used to generate an intermediate amount of propagation phase contrast. A total of 30 closed-beam and 50 open-beam reference images were taken for flat- and dark-field correction. These conditions gave a total imaging time of exactly 240 s for each sample.

Each radiograph captured 4160 µm of lateral distance, and 3120 µm of longitudinal distance in the hyphal compartment / tube.

### 1.7.2 TOMCAT, Swiss Light Source

Samples were mounted for imaging in the same sample stage used at I13. Samples were aligned with a knife-edge to allow physical registration of samples for later resin embedding and sectioning protocols. As it was not possible to reset the datum rotation to a user-defined value, the offset of the knife edge to the incident beam vector was 62.0$^{o}$, as measured at the metrological laboratory of the University of Southampton (Figure S1.7).

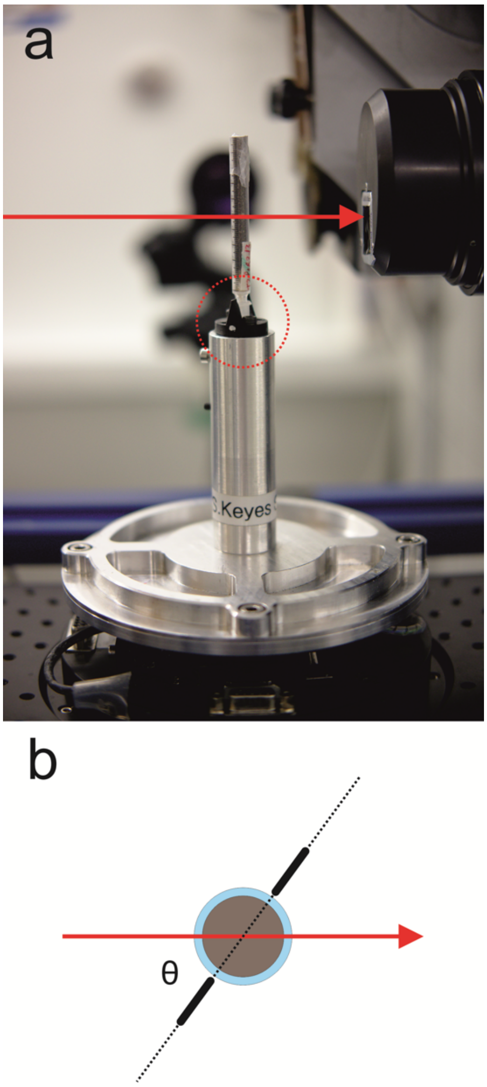


*Figure S1.7 – The samples were mounted in a special rig (a), with knife-edges for sample alignment (red circle). The incident beam axis is shown in red. A top-view schematic shows the knife-edge setup, and the angle (θ) to the incident beam, which was 62º (b).*

For each hyphal compartment, three vertical positions were chosen for imaging. The *intermediate* position ($h_2$) was set as the 0.5 ml gradation, and the *near-root* ($h_1$) and *far* ($h_3$) positions were set 1500 μm below and above this central line, limited by the travel of the stage (due to actuation equipment needing servicing).

Samples were imaged under monochromatic beam conditions, using an energy of 19 kV and 250 μm of Al between the monochromator and the sample. The transmitted X-ray signal was scintillated by a LuAG:Ce film of 20 μm thickness, and optically magnified (using a UPLAPO4x objective) by a factor of 4x. The resulting signal was collected using a PCO Edge 5.5 camera. A total of 1500 projections were acquired for each scan using a 120 ms exposure. A 50 mm sample-to-detector distance was used to generate an intermediate amount of propagation phase contrast. A total of 30 closed-beam and 50 open-beam reference images were taken for flat- and dark-field correction. These conditions gave a total imaging time of exactly 180 s for each sample.

Each radiograph captured 4160 μm of lateral distance, and 3120 μm of longitudinal distance in the hyphal compartment.

## 2 Volume reconstruction

Two reconstruction methods were used. For data collected at DLS I13 (*i.e.* the *Gigaspora* scans, and the 2nd set of P+/P- control scans), only Absorption reconstruction was available. For data collected at SLS (*i.e.* all *Rhizophagus* P+/P- scans and the 1st and 3rd sets of control scans), both Paganin and Absorption reconstruction were available. *Phase* volumes were reconstructed from modified radiographs which had been passed through a *Paganin* phase retrieval filter. The *absorption* volume was reconstructed from unfiltered radiographs in which the phase shift information is contained in the reconstructed volume up as simple edge enhancement.

For application of the Paganin filter, δ and β parameters were required (http://henke.lbl.gov/optical_constants/getdb2.html). Prior to beginning the imaging experiment, different values of δ and β were tested, being the corresponding constant pairs for $SiO_2$, and the so-called ‘biological constants’ ($\delta=3.7 \times 10^{-8}$ and $\beta=1.7 \times 10^{-10}$). Analysis of image quality parameters was carried out for reconstructed slices of each setting. Contrast-to-noise ratio (CNR) was measured using sampled voxels from primary, mixed and pore phases. Edge overshoot checks were carried out by plotting line profiles bisecting the interface between primary and pore phases. Carrying out these quantifications on a characteristic hyphal compartment, for reconstructions using the different Paganin parameter ratios, the biological constants produced slightly lower CNR values (~25% lower), but introduced considerably less image smoothing (Figure S1.8). The CNR characteristics using the biological parameters were more than adequate to allow segmentation of the different phases, so the biological parameters were used on the basis that more fine detail was preserved in the images. These parameters were used for all subsequent *phase* reconstructions.

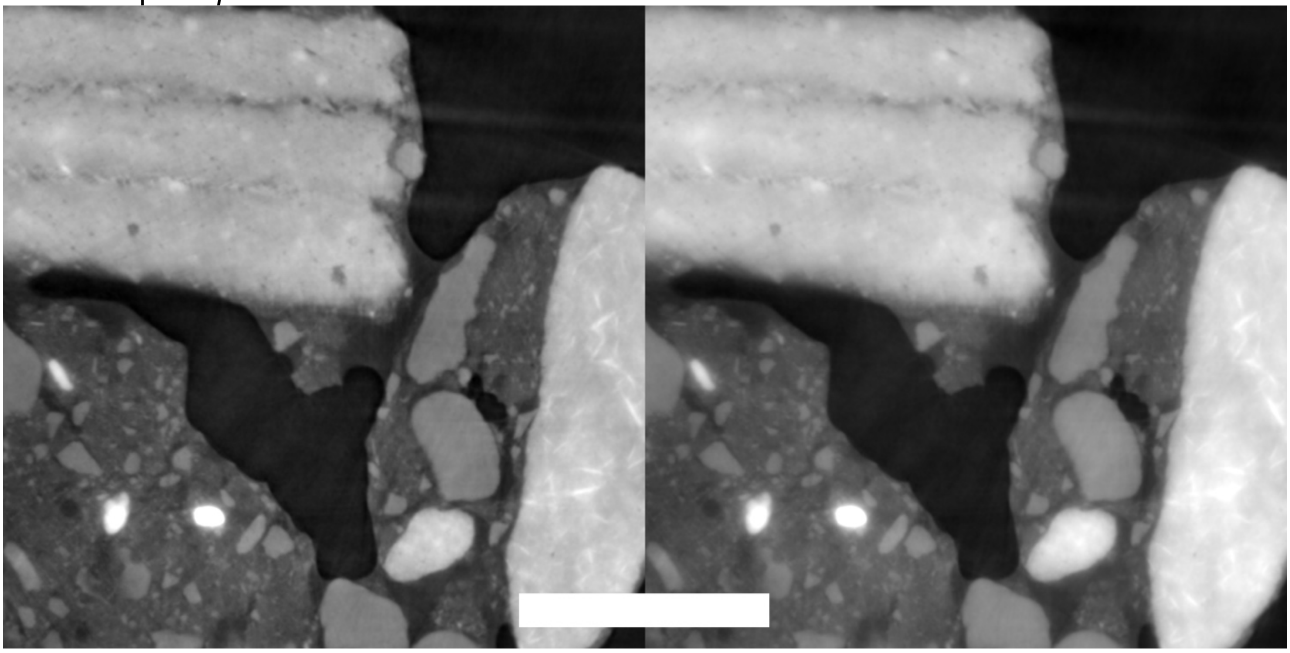

*Figure S1.8 - Data reconstructed using Paganin filter with biological parameters (left) and parameters computed for SiO2 (right). SiO2 parameters improved CNR by ~25% but introduced visible smoothing artifacts (right). (Scale bar is 500 μm).*

To reconstruct *absorption* and *phase* volumes from the respective radiograph sets, a *gridrec* algorithm (Marone and Stampanoni 2012) was used, with a ramp filter and frame padding, generating 16-bit volumes of 2580 x 2580 x 1920 voxels, with an isotropic edge

length of 1.625 μm (SLS) and 1.6 μm (DLS). The sampled region for each scan corresponded to a cylinder of height of ~3120 μm and diameter ~4150 μm, aligned with the centre axis of the hyphal compartment.

Paganin reconstruction was not available at DLS I13, so only *absorption* volumes were reconstructed for these scans (*i.e.* *Gigaspora rosea* P-, and 2$^{nd}$ P+/P- control set).

## 3 Pre-filtering

Files were down-sampled to an 8-bit range and cropped to a diameter of 2000 x 2000 voxels, removing the most poorly sampled region at the fringe of the image furthest from the rotation centre. This was carried out to reduce file-size and make the storage and processing overheads manageable for the large volume of scans.

Data was down-sampled from 16-bit to 8-bit to reduce computational overheads during the image processing stage. The scaling values were set to maximise the usable dynamic range. This was achieved by sampling the central slice from each file, generating a stack histogram of the set of central slices, then cropping to the values on the histogram tails at which the grey level frequency had dropped to 0.005 of the peak value.

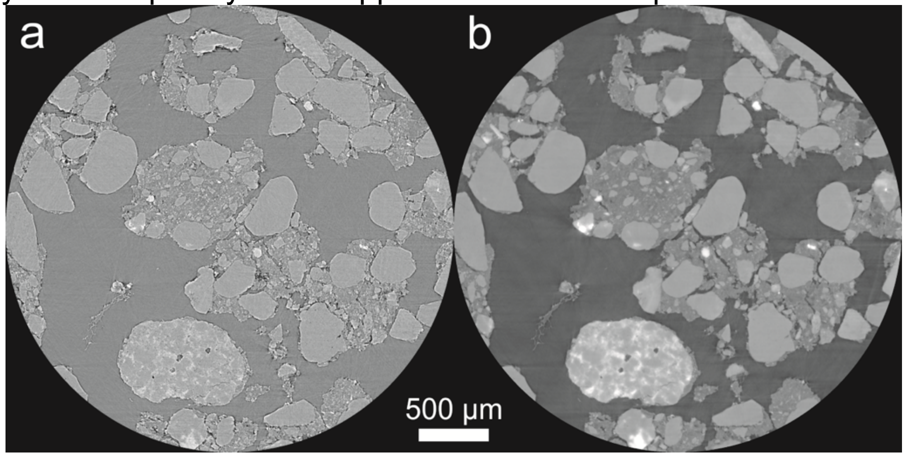


*Figure S1.9 – Representative 2D slices through reconstructed absorption (a) and phase (b) volumes of hyphal compartment SXRCT scan.*

Figure S1.9 shows how the different contrast properties of the phase and absorption reconstructions led to different applications in the image analysis workflow. In all cases, absorption reconstructions were used for hyphal extraction, due to the higher contrast of hyphae vs background produced by the edge enhancement (as a result of propagation phase contrast). Where available (*i.e.* the SLS data of the main inoculated vs control samples in P+/P-), the *phase* reconstruction was used to produce a mask of the soil phases, in order to produce an alternative quantification of the mixed vs primary regions (due to the better inter-material contrast produced by the Paganin filter). Briefly, masking is a process comprising a logical AND operator applied between a binarised region segmented from an image (set to a greylevel value of 1) and greylevel image data, to bound the greylevel data to regions corresponding with the spatial extent of the masked pixels. Where only *absorption* reconstructions were available, these were used for both hyphal and soil classification.

# 4 Digital image analysis

## 4.1 Soil classification

The accurate classification of the soil mineral phases is important for two reasons. Firstly, the overall segmented geometry of all soil phases (*i.e*. delineating the solid/pore interface) provided a mask which could be subtracted prior to the extraction of hyphal structures. This reduced the complexity of the hyphal extraction process by prior deletion of the complex soil structure, which might otherwise have been co-classified along with hyphae. Secondly, the distribution of phases in the soil domain is important for image-based mathematical modelling, which can be applied to mesh representations of volumes and/or surfaces in the soil.

The soil can broadly be said to comprise air-filled pore spaces (*pore*), *primary* mineral grains (*i.e*. quartzes), and a *mixed* phase which comprises particulates of clay, silt and organic matter (and very small pores) at around the imaging resolution and below (Figure S1.10). In the case of the SXRCT data presented here, the pixel size coincides with the standard quantitative classification of the clay fraction (<2 μm). Granular media with particle sizes at and below the imaging resolution appear as textured bulk materials due to the smoothing influence of the partial volume effect on the particles which make up the matrix (*i.e*. Figure S1.10, panel b, region 2).

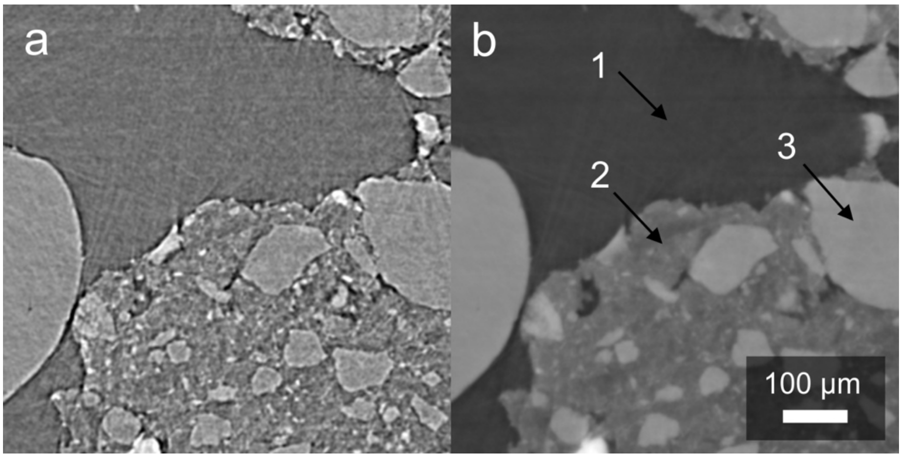


*Figure S1.10 - An identical sub-region shown in absorption (a) and phase (b) reconstructions, showing the characteristic appearance of pore (1), mixed (2) and primary mineral (3) phases at the SXRCT imaging resolution.*

For pore-scale image-based modelling, the distinction between the primary and mixed phases is known to be important, since the reactive and hydrological properties of the phases differ, so does the ability of plant root structures and other soil biota (potentially including mycorrhizae) to infiltrate the two domains. It is therefore important to consider them as separate regions for the purposes of any future model parameterisation.
This required an image analysis protocol for soil classification which could discriminate between primary, mixed and macro-pore phases. Analysis of the contrast between different regions in *absorption* and *phase* volumes is intuitively achieved by comparing the grey-level variation along line profiles which traverse the phases of interest (Figure S1.11).

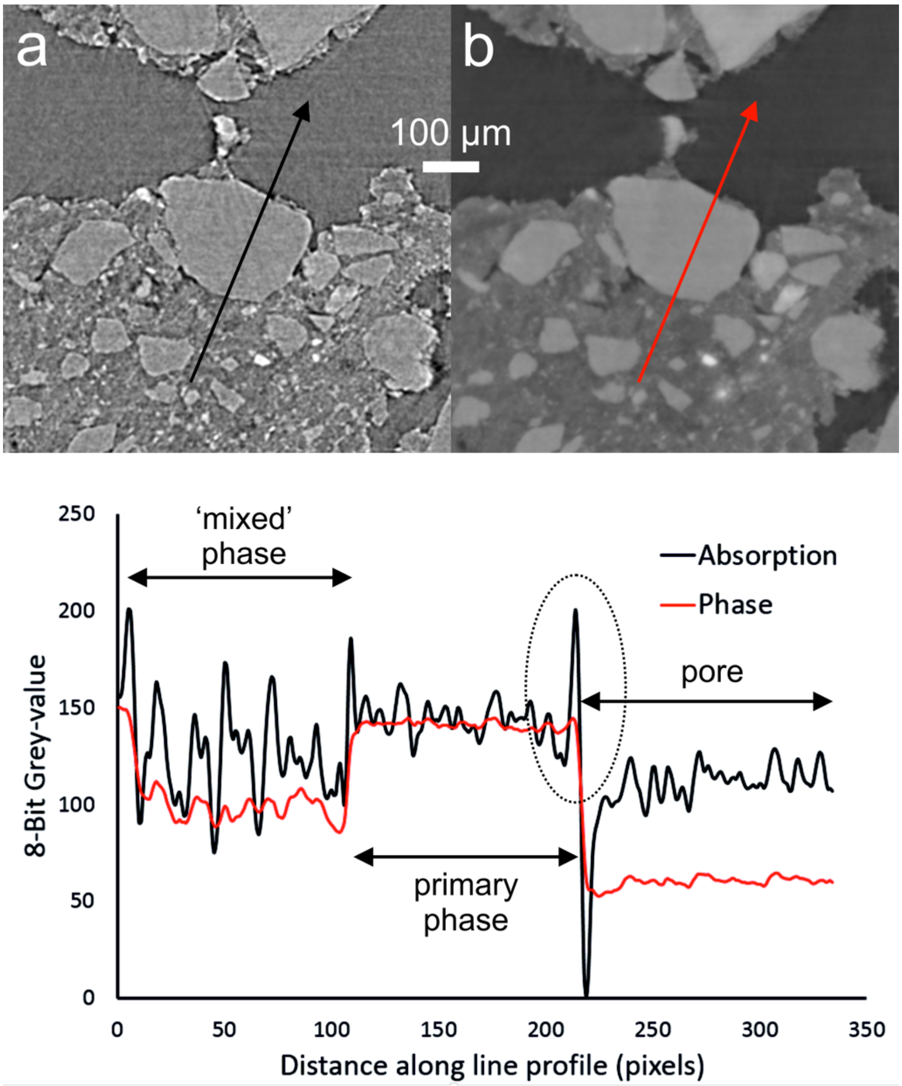


*Figure S1.11 - Plots of grey-value along line profiles in the mixed, primary and air-filled pore phases show the contrast differences in absorption (a) and phase (b) reconstructions. In the plot for the phase image, the profile is less noisy with higher contrast. An overshoot edge-artefact is seen in the absorption line (black) as the profile transfers between primary and pore phases (circled on plot).*

Only the *phase* data allowed clear delineation of the three soil materials on the basis of bulk grey-level value, which is why this reconstruction modality is useful in this case. The edge enhancement artifacts in the absorption data (seen clearly in the overshoots at the edges of the primary phase) are due to the propagation of phase shifts produced at material interfaces, which are considered as part of the absorption signal in the reconstructed data. In the *phase* image, the contribution of these phase shifts to beam intensity are extracted explicitly from the attenuation component using a Paganin filter, and thus the edge artifacts are suppressed.

The process for extracting these soil phases using image processing approaches is detailed in section 5.1 below.

## 4.2 Hyphal classification

For hyphal extraction, for which the characteristic diameters can be down to near the imaging resolution, the edge-enhancement artifacts in the absorption volume (which are problematic for soil classification) are an aid to feature extraction. The edge enhancement

of the gas/hyphal interfaces produces much higher pore-to-hypha contrast than is observed in the phase data (Figure S1.12).

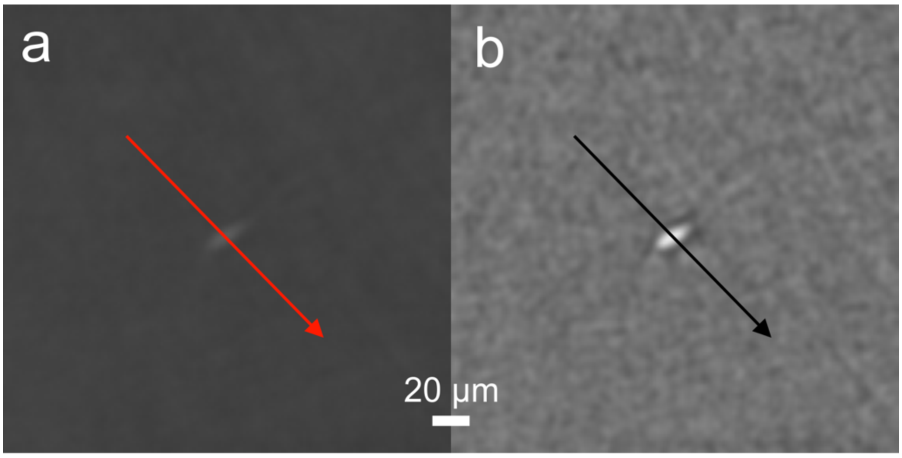


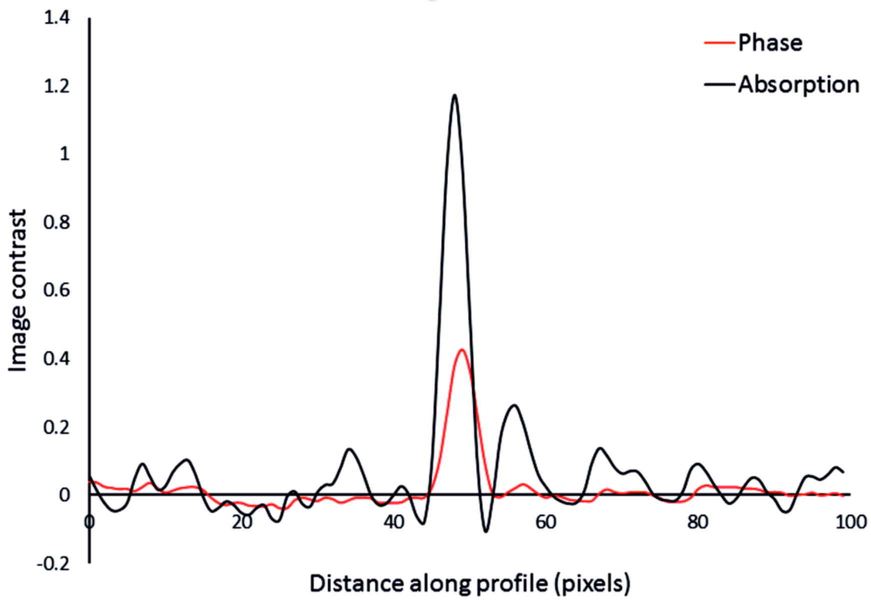


*Figure S1.12 - Plots of image contrast along line profiles of the same hypha in both phase (a) and absorption (b) reconstructions. In the plot for the absorption image, there is ~3 times more contrast between the hypha and the pore background. (Sample T4PmR1D1h1, Slice 970, SLS TOMCAT).*

Due to their differing contrast characteristics, the absorption data represents a better basis for hyphal classification, and the phase data a better basis for soil classification. The entire workflow for data extraction therefore proceeds with both reconstructed volumes being used from each SXRCT scan (where available - only *absorption* volumes were available for I13 data).

The process for extracting the hyphal phases using image processing approaches is detailed in section 5.2 below.

## 5 SXRCT data processing workflow

This section provides detailed technical description of how image segmentation was achieved.

## 5.1 Soil classification

The first stage in data processing was to split each scan into mineral and non-mineral components. Since the hyphae were not distinguishable within the soil mineral phases, these components of the image were masked out to simplify the processing of the pore-based hyphal fraction. This required a soil classification approach, as shown in the schematic in Figure S1.13.

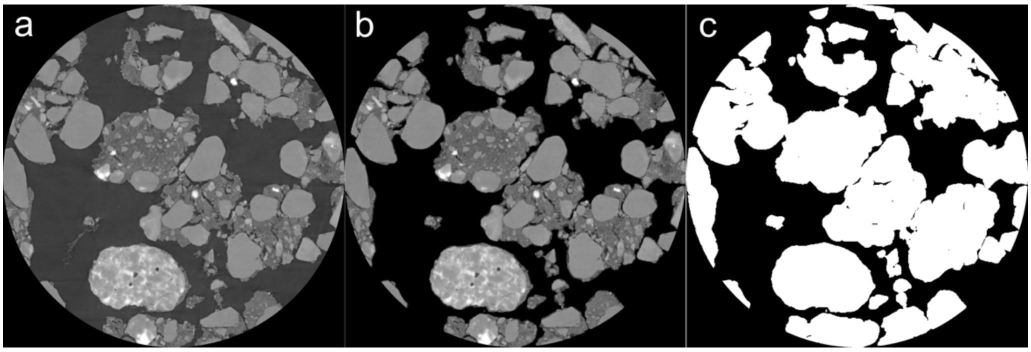


*Figure S1.13 - The raw phase volume (a) is segmented using WEKA to robustly reject macro-pore and hyphal voxels (b). The resulting soil phases are then binarised to form a mask (c).*

To classify the mineral phases without including hyphae and spores, a WEKA machine-learning approach was used (Daly, Keyes et al. 2015, Keyes, Cooper et al. 2017, Koebernick, Daly et al. 2017). This method classifies voxels using image measures that are sensitive to local grey-level statistics rather than simple grey level intensity. This allows, for example, the hyphae to be excluded from the soil segmentation in a manner that is not possible on the basis of grey level differences alone (i.e. by using approaches such as grey-level thresholding or region growth). This is important, as any hyphae wrongly classified as soil would be masked out of the data at a later stage, when the soil classification is used to mask voxels from the absorption reconstruction prior to hyphal segmentation (Section 5.2.1).

For the *phase* volume of each scan (in the case of the SLS data), or the *absorption* volume of each scan (in the case of the DLS data) a set of 20 slices was sub-selected manually, being specifically identified as including representative examples of all artifact features (*i.e.* rings and streaks), and fungal structures (*i.e.* hyphae and spores). It was important to train the WEKA classifier on examples of these features so that the final soil segmentation did not erroneously co-classify hyphae or artifacts. The 20 slices from each volume were user-defined to include such features. For volumes in which clear hyphal colonisation was evident, two sub-volumes representing the most prominent fungal structures (*i.e.* largest spores, densest branching, thickest hyphae) were cropped out and saved for additional classifier testing.

A WEKA classifier was then trained using a set of image measures (in this instance the Entropy, Hessian, Neighbours and Variance), and sets of voxels determined by the user as belonging to various classes. In this instance, the classes were simply *soil* (the primary and mixed phases) and *non-soil* (air phases and fungal structures). In an iterative manner, sets of training voxels were added to each of the two classes, from the 20-slice training stack defined in the previous step. Voxel regions from of all artifacts visible, and all hyphal structures, were added to the non-soil class. The training process is iterative, with more voxels being added to the classes at each step, until the visual feedback (Figure S1.14) indicates that the segmentation is not misclassifying the different phases. This training was carried out across the 20 training slices for each sample, producing a set of training voxels for all 87 samples.

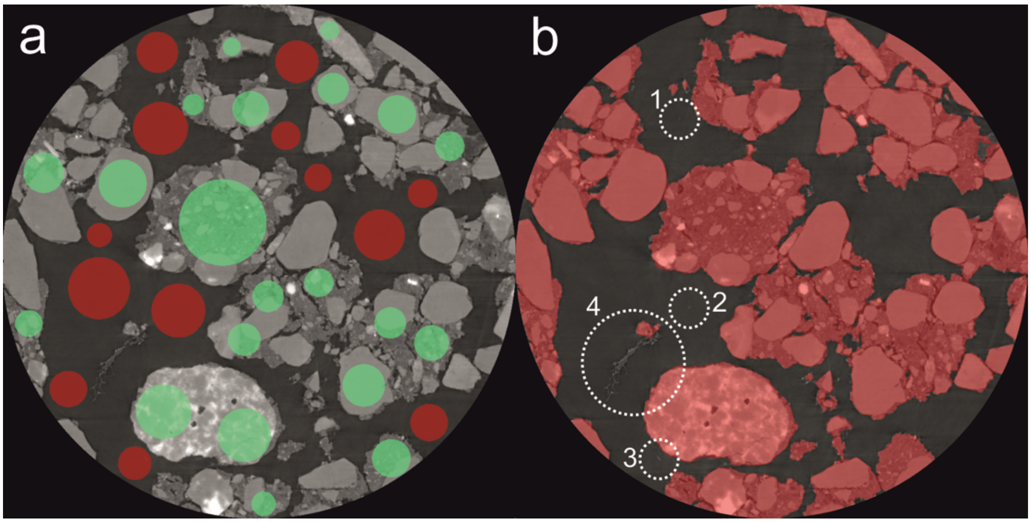


*Figure S1.14 – To parameterise WEKA, user-defined voxel regions are created, representative of soil mineral regions (green), and pore/hyphal regions (red) (a). Overlaying the result demonstrates the performance of the classification (b). Hyphae remain unclassified (1-3), but some large pieces of organic matter also remain unclassified (4).*

After initial iterative training, the classifier for each sample was saved and an initial WEKA segmentation carried out on the 7 unseen image slices that were not part of the training stack, and on the two hyphal sub-volumes. The output for each classification run is a 32-bit probability map, with voxel values varying between 0 and 1, in which the floating-point value represents the probability that a given voxel belongs to the *soil* class rather than the *non-soil* class. Iteratively thresholding this map between 0 and 1 in steps of 0.1, then overlaying the result with the original grey level data, allowed the accuracy of the segmentation to be determined (Figure S1.15).

Adequate segmentation of the soil was determined by visual inspection of these overlay outputs across all scans, with the criteria being: a) conservation of soil/pore interface topology, relative to the interfaces visible in the grey level data, and b) a complete absence of hyphal morphologies and/or artifacts such as streaks or rings being erroneously assigned to the soil class.

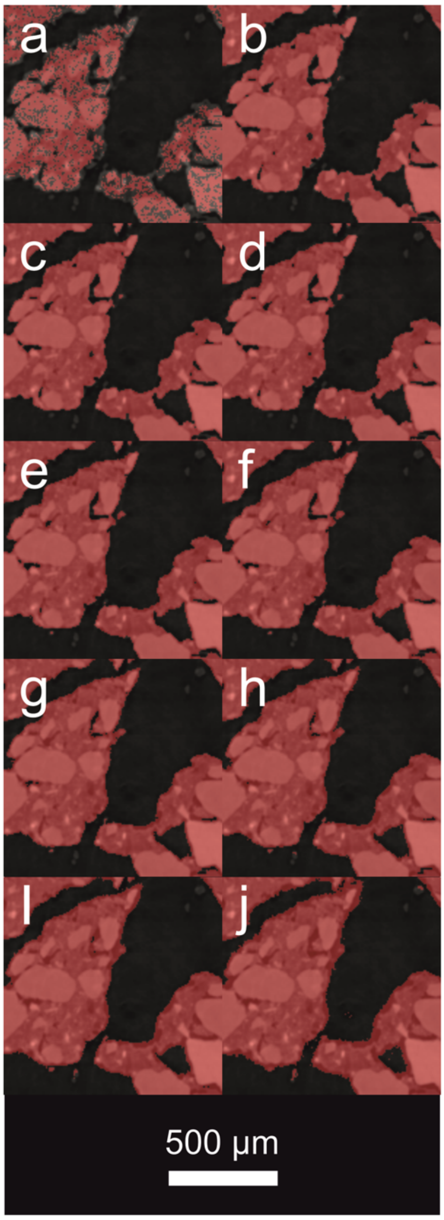


*Figure S1.15 –WEKA segmentation results between 1 and 0.1 in steps of 0.1 (a-j), overlaid on the original grey-level image. The value at (a) is clearly under segmented, whilst values at (g-j) clearly over-segment the pore space.*

On the basis of this user-involved assessment, to find the minimum threshold value at which misclassification did not occur, a thresholding range of 0.6-1 was found to suppress erroneous classification of hyphae across all samples.

The 4x spatially downscaled, 8-bit grey level volume of every scan (“wekaTiff.tif”) was analysed in this way to produce a custom classifier which was checked for hyphal classification errors. Classification was carried out via a batch script which automated the process of applying the correct classifier to the correct scan, and saving the 32-bit probability map.

The run-time for each WEKA segmentation was ~ 6 h per sample. Following application of the 0.6-1 global threshold to binaries the soil and non-soil regions, the mask was saved for use in the hyphal segmentation step (5.2), and also applied via an AND operator to the *phase* reconstruction, producing a 3D volume of attenuation coefficients in the soil mineral

regions alone (for visualisation – see Figure S1.13 b). Otsu thresholding[1] of this masked soil grey level data also produced a 3-phase simplified representation of the pore, primary and textural phase regions (Figure S1.16) for use both in image-based models and to determine respective volume fractions of primary and textural phases.

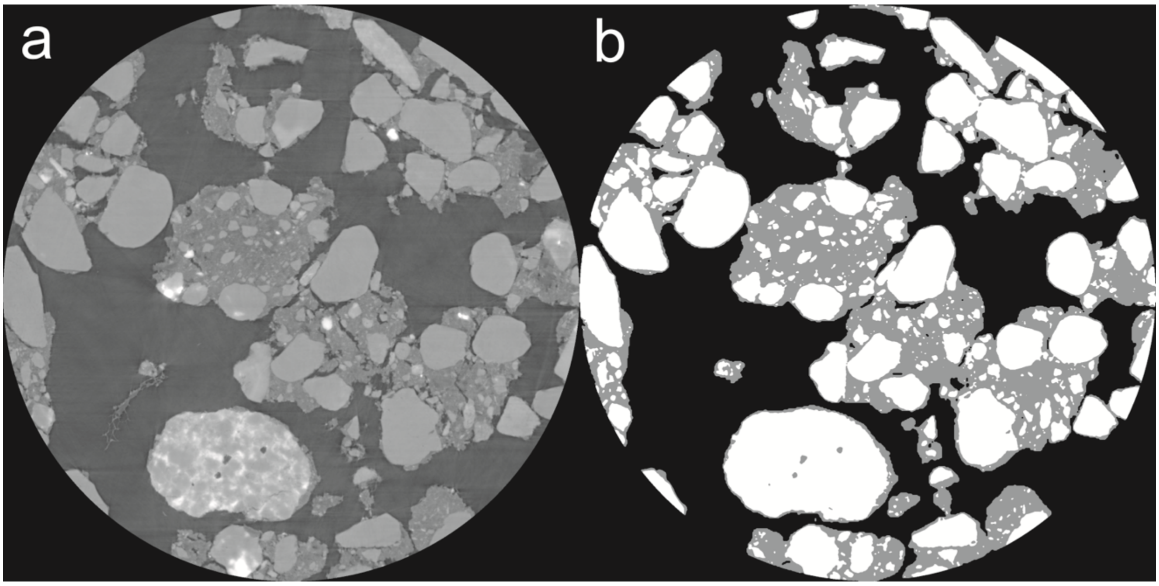


*Figure S1.16 – A slice of a phase volume (a) and the simplified representation of primary (white), mixed (grey) and pore (black) regions to be used for mesh-generation in image-based modelling (b).*

## 5.2 Hyphal classification

Hyphal classification was carried out using the *absorption* volume, due to the greater hypha-to-pore contrast (Figure S1.12). Classification was carried out using an edge-enhancement approach which allowed hyphal paths to be extracted using global grey-level thresholding (full technical details below). The 3D results of this threshold were then filtered using a morphometric approach, removing erroneous objects that were non-hyphal in their morphology (full technical details below).

### 5.2.1 Masking of soil mineral regions

Before extracting hyphal paths from the pore domain, the soil mineral phases were masked from the *absorption* volume using the binarised soil mask generated as the final output of the WEKA classification step (*i.e.* Figure S1.13c and Section 5.1). The mask required dilation in order to overcome a region of high grey-level gradient at the mineral/pore interfaces, resulting from the edge-enhancement overshoot artefact (Figure S1.11). This was necessary because the hyphal segmentation step (described below) uses an edge-enhancement filter (Sobel) which is very sensitive to local gradients, and the overshoot artefact at the soil-to-gas boundary would otherwise be classified in this step. To find the optimal dilation parameter for this correction (*i.e.* the minimal number of dilations of the soil mask required to satisfactorily overcome it, preventing the Sobel operator from detecting the gradient), samples were analysed using an Euclidean distance transform (Figure S1.17). By quantifying the mean grey-level in the pore phase with increasing distance from the soil/pore interface (*i.e.* in 3D annuli at increasing distance), a distance

[1] Otsu thresholding method involves iterating through all the possible threshold values and calculating a measure of spread for the pixel levels each side of the threshold, i.e. the pixels that either fall in foreground or background. The aim is to find the threshold value where the sum of foreground and background spreads is at its minimum

could be determined at which the artefact was no longer prominent. This distance was set to be to the integer value of dilations that coincided with the soil-ward size of the maxima (Figure S1.17).

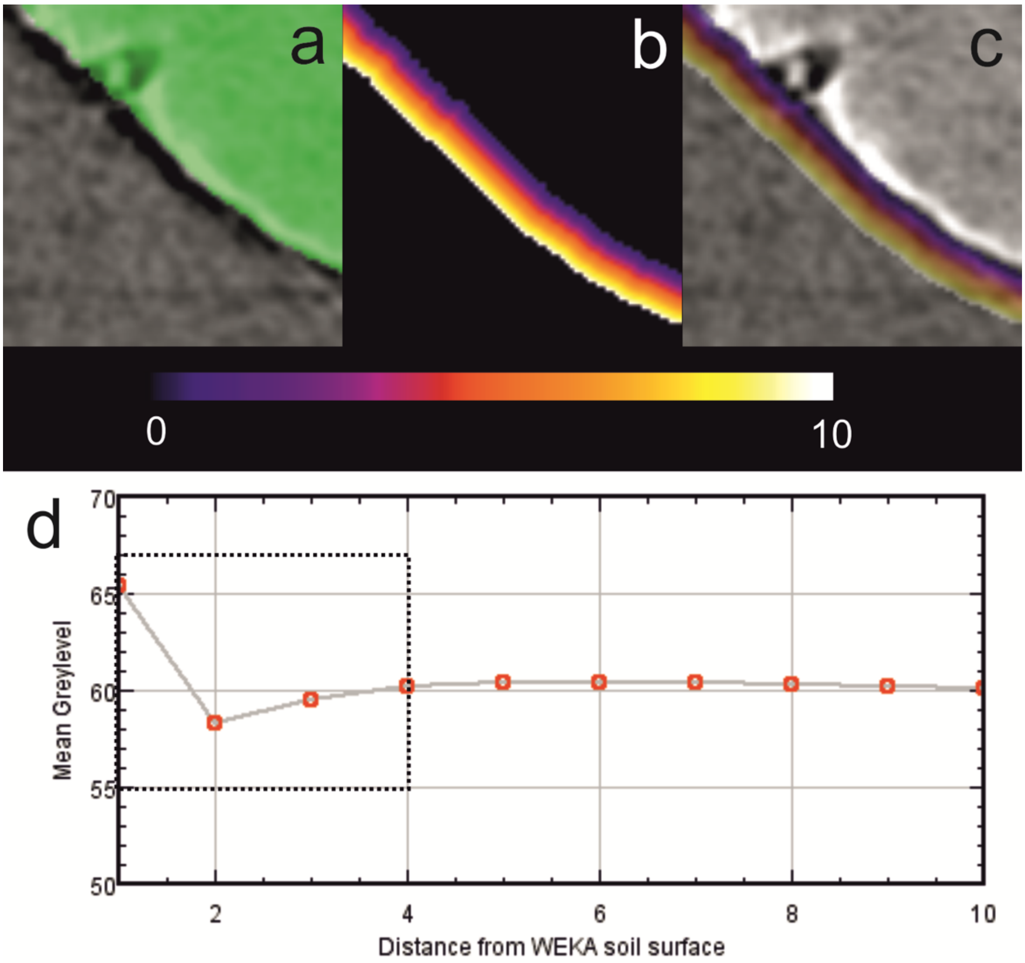


*Figure S1.17 - Because of the edge enhancement from phase-contrast in the absorption volumes, the pore voxels immediately outside the soil mask (in green) have much lower grey-values than the bulk pore-space (a). A distance map is generated in the pore-space, where the pixel value (shown as the colour-map) takes the closest straight-path distance to the mask surface (b). Taking the average grey-value of voxels in the absorption volume which correspond to each discrete distance step (c), a plot of mean pore grey-level with distance from the soil mask can be produced (d). It is evident that within the distance bounded by the dotted line, there is a strong gradient. The region was masked out by dilating to 4, the integer value to the left of the maxima. Distance units are pixels.*

Following this 3D dilation step, the soil mineral mask was used to remove corresponding voxels in the raw *absorption* volume (Figure S1.18).

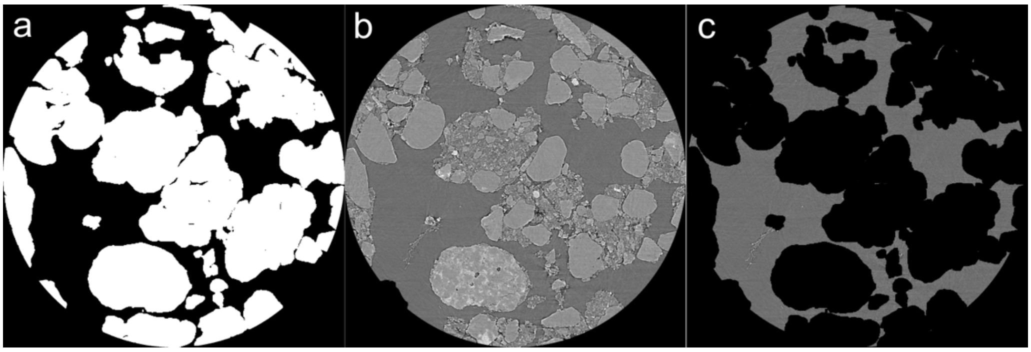


*Figure S1.18 - The mask generated from the phase volume is dilated slightly (a) and subtracted from the absorption volume (b) to remove the voxels representing the solid soil domain (c). All the SRXCT workflow schematics are shown on slice 970 of sample T4PmR1D1H1.*

The mean grey level of the pore phase was then measured by taking 20 sample measurements across the central slice of each of the *absorption* volumes. The resulting grey-level value was used to fill in the masked soil domain of each *absorption* volume (Figure S1.19). This step ensured that the only significant local gradients in the image occurred between the pore-space and non-soil objects (*i.e*. hyphae and organic matter).

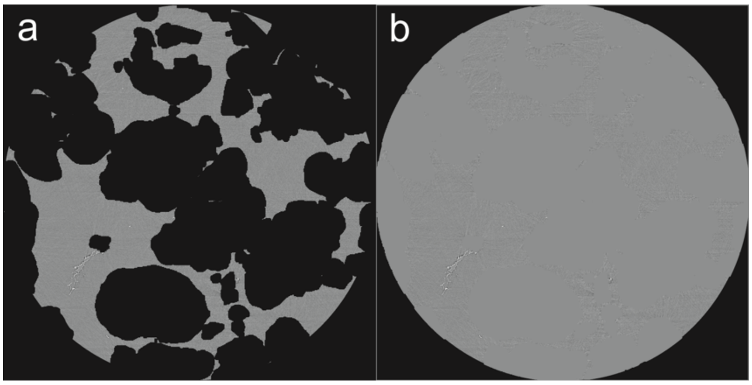


*Figure S1.19 - The masked absorption image (a) has the masked voxels replaced with the mean grey-level value of the macro-pore space (b).*

A maximum/minimum filtering step (with a kernel size of 3 voxels) was then applied to reduce salt-and-pepper noise in the pore space, and boost the contrast of the hyphae against the pore background. A 3D Sobel operator applied to this result produced a map of local grey level gradient, which was maximised at hyphal/pore interfaces. An open/close operation (with a kernel size of 3 voxels) was applied to smooth this map, ensuring that none of the hyphal geometries ended up with a ‘hollow’ tube morphology (Figure S1.20 and Figure S1.21).

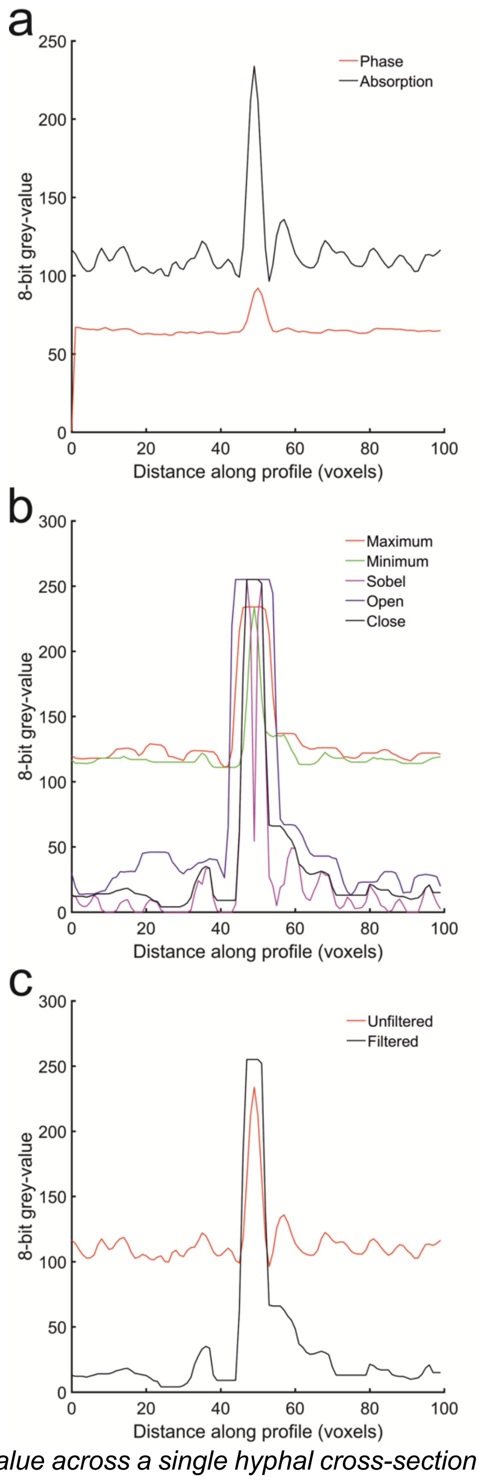


*Figure S1.20 – (a) Plots of grey-value across a single hyphal cross-section shows the improved contrast of the absorption reconstruction. (b) The iterative filters applied to the absorption reconstruction serve to amplify this contrast further (the filters are additive, and are applied in the order top>bottom). (c) The final contrast-boosted output is shown relative to the input.*

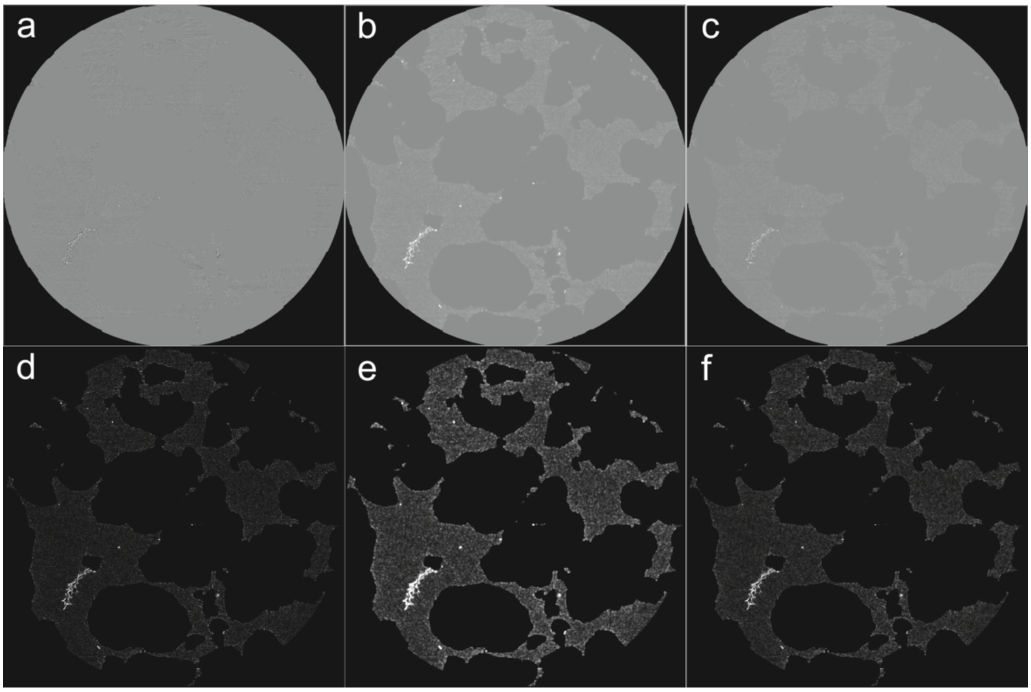


*Figure S1.21 - The masked absorption volume (a) is passed through maximum (b) and minimum (c) filters, a Sobel edge filter is applied (d), and an open (e) and close (f) filter are then used to smooth the edge intensity map.*

The output of these steps was a volume for each scan with substantially boosted hyphal/pore contrast (Figure S1.21). The output of the edge-enhancement filters was 3D-rendered over a range of threshold values (20 – 40 with a step size of 1), allowing initial visualisation of hyphal distribution and density, and the selection of an optimal cut-off threshold for binarisation (*Figure S1.2222*). After assessment of these iterative maps for all 87 samples, an intermediate value was selected minimising the degree of both noise and misclassified features (*i.e.* organic soil components and streak/ring artefacts in the pore phase) whilst maintaining hyphal connectivity. This was assessed by scaling back from an over-segmented condition and noting the point at which continuous hyphal filaments began to become disconnected. The threshold was set to the most conservative value across all 87 samples, and corresponded to a threshold of the 8-bit range 20 - 255.

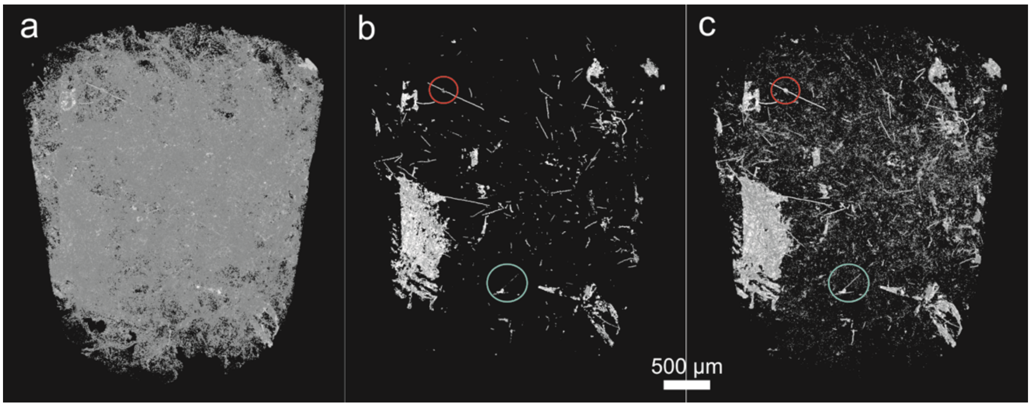


*Figure S1.22 - Examples of the output of the hyphal extraction filter, rendered after binarisation at different threshold values. Too conservative a value results in over-segmentation, where continuous regions of noise are evident (a). Too aggressive a value strongly suppresses these erroneous features, but also removes hyphal structures (examples in circles). An intermediate threshold (20-255) conserves hyphal morphology whilst minimising noise (c).*

## 5.3 Filtering of hyphal geometry

After the hyphal extraction step, the binarised data still contained artifacts in the form of noise and organic material (*Figure S1.2222*c). Since the grey-level ranges of these features overlapped those of the hyphae, a grey-level based approach to their removal was not feasible. Manual segmentation approaches were also unfeasible due to the number of samples involved. An automated filtering method was thus developed based on object morphometry. These approaches are predicated on quantifiable differences in the shape descriptors of discrete 3D objects in the sample.

The morphology of pore noise features is characterised by discrete 'particles' of very small volume; see the more detailed discussion in the later section and Fig S1.24. The morphology of non-hyphal organic matter features is characterised by complex surface topology and a very large range of object volumes. The hyphal morphology is characterised by long and filamentous structures of largely consistent diameter. On the basis of these different morphometric descriptions, a 2-pass morphological segmentation protocol was devised, incorporating a particle and skeleton filtering stage.

### 5.3.1 Particle filtering

The particle-filtering step was parameterised by output data from FIJI's *Particle Analyser* plugin (Doube, Kłosowski et al. 2010), which performs morphometric quantification of discrete binarised 3D objects in volume datasets. The result is a list of object IDs, with a large number of parameters (i.e. volume, surface area, Euler number, thickness, centre of mass) quantified for each discrete object. To understand which measures had discriminatory power for hyphal filtering, a *ground truth* set of objects was manually defined, by classifying every object in a representative training volume into *hyphal* and *non-hyphal* classes. This allowed histograms of various parameters to be plotted for these two classes independently (Figure S1.23).

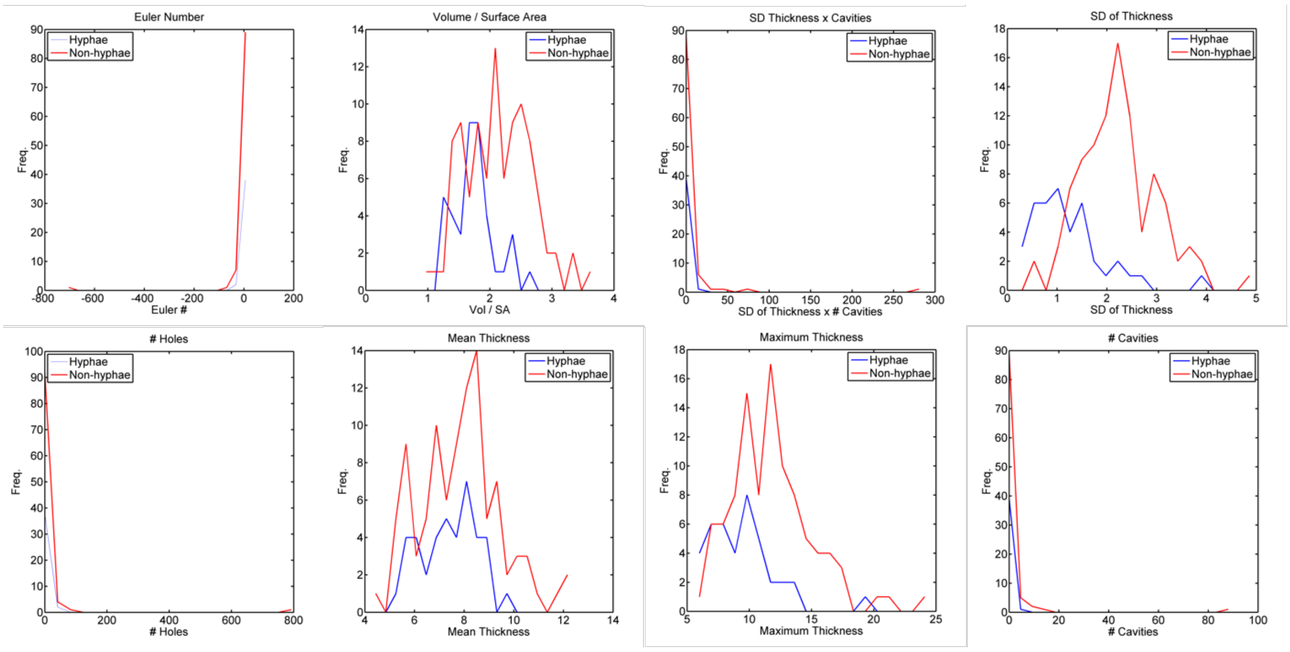


*Figure S1.23 – Histograms for selected morphometric parameters. The degree of separation between the distributions is suggestive of the suitability of the measure for discriminating between the two classes. All quantities are shown in pixels.*

Though some of the measures provided a degree of separation, the better suitability of the skeleton filtering approach (below) and the requirement to minimise hyphal loss indicated that a simple volumetric filter was the only necessary constraint.

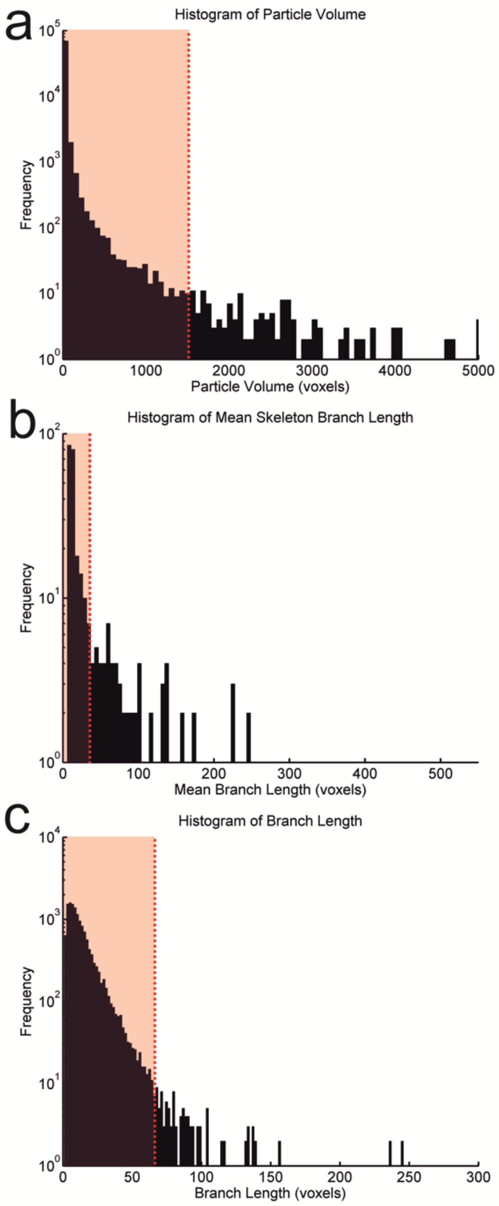


*Figure S1.24 – (a) A histogram of particle volume at the 20 – 255 threshold (note log scale) indicates that the majority of the noise-related objects have a very small characteristic volume. Particles were removed with volume <1500 voxels, which corresponds to the red region. (b) In the volume-filtered image, all visible non-hyphal organic artifacts had a mean skeleton branch length of < 30 voxels (red region). (c) Splitting rejected objects into component branches, lengths of >60 were observed to correlate with hyphae which happened to touch organic artifacts.*

A volume of 1500 voxels was selected as the lower cut-off value for the particle filter (Figure S1.24a), which suppressed virtually all SXRCT-related noise artifacts (Figure S1.25b). This does limit the quantification to considering discrete hyphal structures of length >~120 um.

### 5.3.2 Skeleton filtering

Having removed noise artifacts of small volume, the remaining objects included both filamentous, hyphal structures (which are often branched and sporulated), and other non-

hyphal organic objects. To conserve the hyphal structures whilst removing the remaining unwanted features, a morphometric skeleton filter was applied.

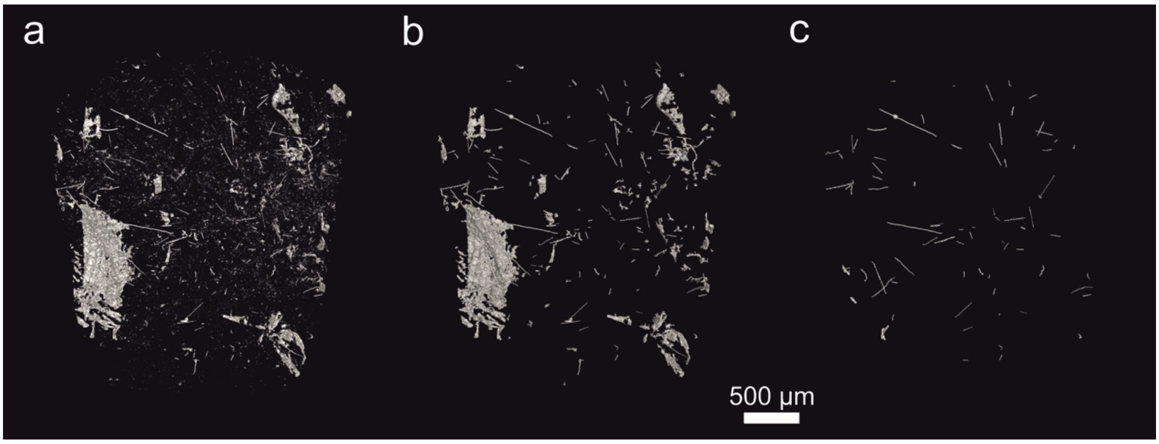


*Figure S1.25 – The iterative results of the morphometric filters. A volume render of the raw, unfiltered result of hyphal segmentation, thresholded from 20 – 255 to conserve hyphal paths (a). After volume filtering of discrete objects, the effect of image noise is suppressed (b). After the skeleton filtering step, only filamentous structures remain (c).*

The input for this step is a skeletonised representation of the binarised 3D geometries in the volume, generated using the *3D Skeletonise* plugin in FIJI. Skeletonisation applies iterative binary-thinning which reduces all foreground/background interfaces of a 3D object to a centreline representation (*i.e*. the 'skeleton'). For thin, filamentous objects such as hyphae, the skeleton representation conserves virtually all the morphological information of the parent object (except a scalar diameter parameter). For topologically complex objects such as organic matter, with convoluted surfaces and internal cavities, the skeletonised representation is usually a complex network of loops and convoluted branches (Figure S1.26). Morphometric filtering of skeleton data is predicated on resulting differences in quantitative measures for these two classes (*i.e*. branching statistics, tortuosity, branch length). Measures such as 'total length' are to be avoided, since the length of hyphae in a given sample is not known *a-priori*, and this would risk removing long hyphal structures. Instead, the measure of mean branch length was employed, which takes a characteristically very small value for complex 3D objects, and can be very large for true hyphae. In a skeletonised representation, perfect spheres reduce to single points (*i.e*. length 1).

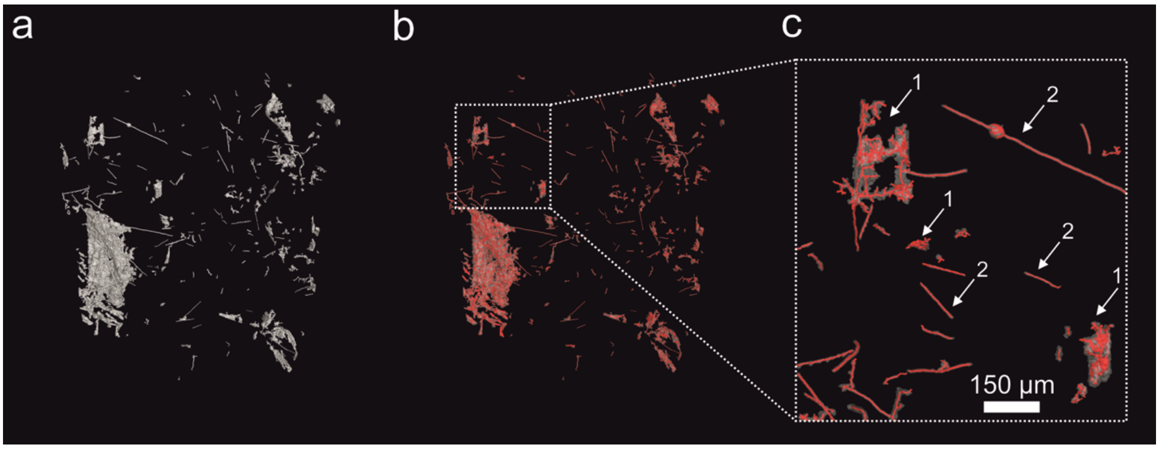


*Figure S1.26 – The particle-filtered data (a) is skeletonised, and shown overlaid (in red, slightly dilated for visualisation) on the original data (b). (c) Detail views show how particles of organic matter skeletonise to complex networks of small branches (examples at 1), but filamentous structures (i.e. hyphae) only lose diameter information (examples at 2).*

The skeletonised output of the particle filter was passed to the *Analyse Skeleton* plugin of BoneJ for quantification. This generated morphometric outputs at both the skeleton and branch level. A collection of joined branches is called a *skeleton*; a continuous segment in which there are no bifurcations or endpoints is called a *branch* (Figure S1.27). For each discrete (i.e. non-touching) skeleton object, *Analyse Skeleton* (Doube, Kłosowski et al. 2010) quantifies the total length, number of branches, number of branch-points, and mean branch length. It also returns the length of every dependent branch in the skeleton and the coordinates of the two endpoints. This allows computation of a vector approximation to the branch path, a straight-line distance, and thus a tortuosity factor (Figure S1.27). A code written in MATLAB took these data and filtered every skeleton on the basis of its mean branch length. The output of this step was a list of output coordinates, each representing the endpoint of a single arbitrary branch in each accepted skeleton, which was imported to FIJI and used to flood-fill the particle-filtered hyphae (i.e. Figure S1.25b) using a *Geodesic Reconstruction* plugin (Legland, Arganda-Carreras et al. 2016) in FIJI.

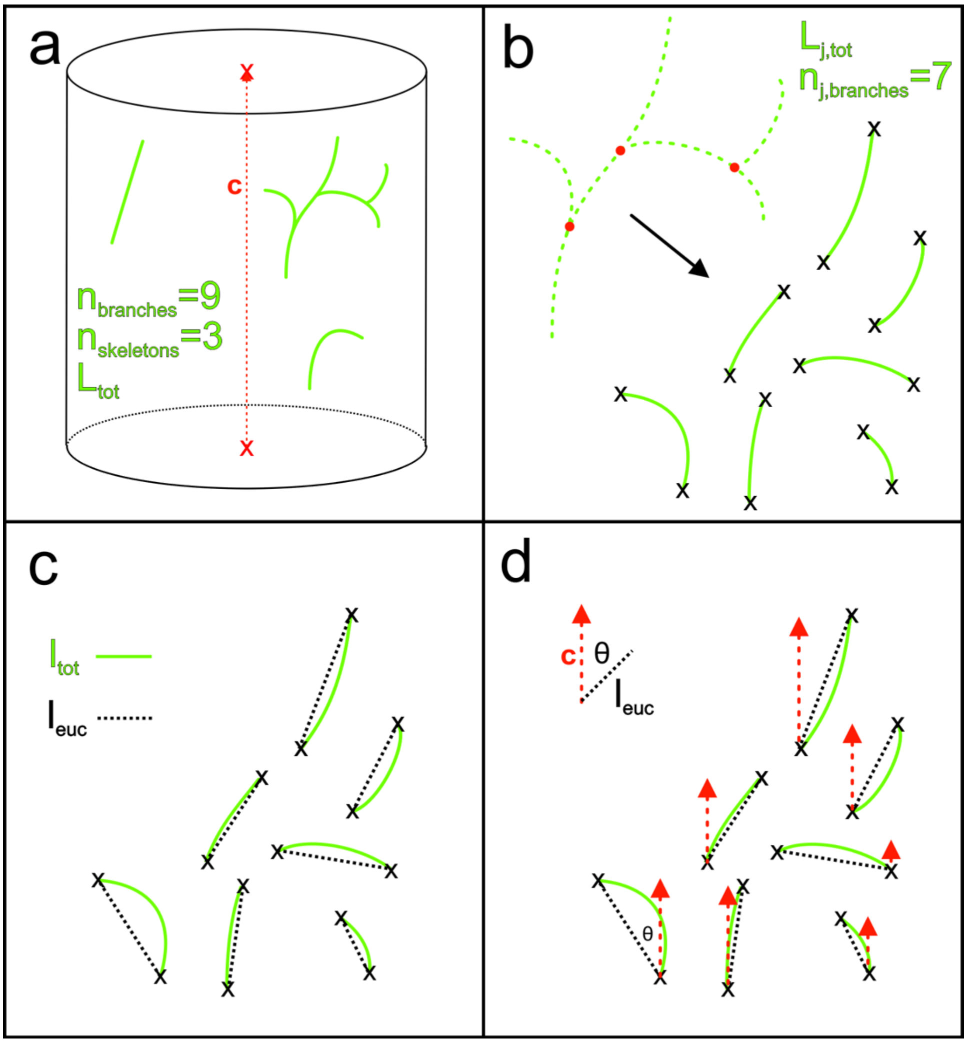


*Figure S1.27 – A schematic of the metrics used to quantify hyphal structures. (a) The entire hyphal content is classified to give the entire length ($L_{tot}$), the number of discrete skeletons ($n_{skeletons}$) and the total number of branches ($n_{branches}$). (b) Each of the j skeletons is then classified by the number of constituent branches ($n_{j,branches}$). (c) The tortuosity of each branch is defined by the ratio of total length ($l_{tot}$) to the length of a vector ($l_{euc}$) linking the end points. (d) The angle of each branch to the centreline is given as the angle from* $0-\frac{\pi}{2}$ *, since the direction of the branch vector is not rigorously defined.*

In the present case, histograms of mean branch length indicated a cut-off for non-hyphal objects (*i.e.* organic matter artifacts), in the region of 20 – 50 voxels (Figure S1.24b).

Setting a conservative mean branch length threshold of 30 voxels gave excellent separation between hyphal and non-hyphal objects, as determined by qualitatively assessing the filtered hyphal structures (Figure S1.25c) relative to the unfiltered 3D images (Figure S1.25a) and assessing both the degree to which the clearly non-hyphal structures were suppressed, and clearly hyphal structures were maintained between the two images. To catch hyphae which were in contact with organic artifacts, and thus rejected in the first pass, a second pass was conducted, in which all individual branches in a disallowed skeleton (*i.e.* mean branch length < 30 voxels) which had a branch length over a certain threshold were accepted. The distribution of individual branch lengths (Figure S1.24c) indicated a suitable cutoff at >60 voxels. These processes were automated using scripts in FIJI and MATLAB, and applied to all 87 samples using batch operations.

## 5.4 Hyphal morphology quantification

Following extraction of filtered hyphal morphology, the hyphal population of each sample was quantified using the measures previously outlined in the skeleton filtering protocol (Figure S1.27). Following skeletonisation of the final hyphal paths, the following metrics were quantified: total hyphal length, number of skeletons, branch count per skeleton, mean branch length per skeleton, tortuosity of branches, and angle of orientation to chamber midline.

Total length of hyphae per sample in mm ($L_{tot}$) is given by:

$$L_{tot} = \sum_{k=1}^{n_{branches}} \left(l_{k,tot}\right), \qquad \text{Eq. S1.1}$$

Where $n_{branches}$ is the number of branches in the sample, and $l_{k,tot}$ is the total length of branch *k.*

The hyphal length density ($L_v$) in mm.mm$^{-3}$ is given by:

$$L_v = \frac{L_{tot}}{V}, \qquad \text{Eq. S1.2}$$

where $V$ is the volume of the domain in mm$^{-3}$.

The mean non-dimensional hyphal tortuosity ($T_{norm}$ is given by:

$$T_{norm} = \frac{\sum_{k=1}^{n_{branches}} \left(\frac{l_{k,tot}}{l_{k,euc}}\right)}{n_{branches}}, \qquad \text{Eq. S1.3}$$

where $l_{k,euc}$ is the length of the vector $e_k = (x_{k,2} - x_{k,1}, y_{k,2} - y_{k,1}, z_{k,2} - z_{k,1})$ which links the endpoints of branch $k$, these points being given by $(x_{k,1}, y_{k,1}, z_{k,1})$ and $(x_{k,2}, y_{k,2}, z_{k,2})$ respectively.

The hyphal angle in radians ($\theta_{norm}$), defined with respect to the hyphal compartment centre-line, is given by:

$$\theta_{norm} = \frac{\sum_{k=1}^{n_{branches}} \left(\theta_k l_{k,tot}\right)}{n_{branches}}, \qquad \text{Eq. S1.4}$$

where $\theta_k$ is the angle between $e_k$ and the chamber centre-line $c$ (over the range $0$ $\frac{\pi}{2}$ ), given by:

$$\theta_k = \left\| \left( \left\| \left( \operatorname{atan}\left( \|e_k \times c\|, e_k c \right) - \frac{\pi}{2} \right) \right\| - \frac{\pi}{2} \right) \right\|, \qquad \text{Eq. S1.5}$$

where $\|a\|$ denotes the absolute value of $a$. This form is used to non-directionalise the angular measurement, such that the maximum value of $\theta_k$ becomes $\frac{\pi}{2}$.
These metrics were computed in MATLAB, along with the standard deviation and standard error in the mean across replicates for each measure.

Box plot and bar charts of these measures were produced, along with correlation plots produced by plotting two measures against one another for each variable to look for patterns. Plots of length density with distance from the root system were produced to aid selection of samples for chemical mapping approaches.

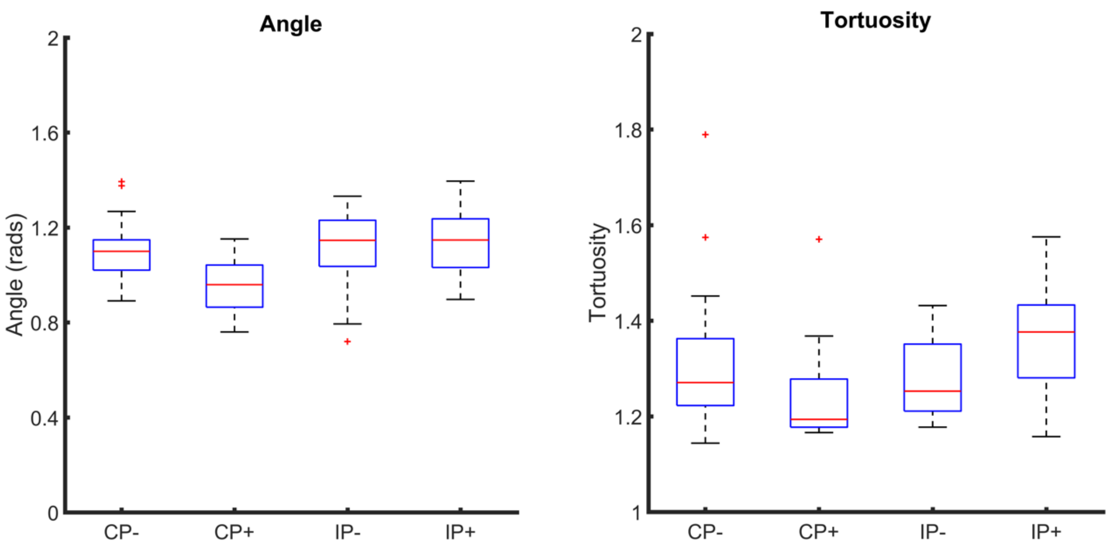


*Figure S1.28: XSRCT based hyphal tortuosity (right) and alignment angle (left) results; blue box describes the inter quartile range i.e. 25%-75% of data falls within this interval, red dots are the outliers and black whiskers show ±2.7$\sigma$ where $\sigma$ is the standard deviation.*

SXRCT imaging is non-invasive and provides richer information than destructive bulk length density measures, enabling estimation of the number of branches per hyphal cluster, branch length, angle and tortuosity. However, as hyphae were only detectable in the pore space due to the contrast limitations of SRXCT, these measures might not be fully representative of the entire soil hyphal population. Control P+ measurements showed significantly higher (t-test p<0.05) and differently distributed (KS p<0.05) branch numbers per cluster (we defined a single cluster to be all hyphae that are continuously connected on the images) than control P- and both P+ and P- inoculated treatments (see Fig 3), suggesting that in this soil, non-AMF hyphae are more branched in the soil pore space than AMF hyphae. The differences in branch number and distribution between inoculated P+ and P- treatments was not found to be statistically significant (t-test p<0.05 and KS p<0.05) potentially indicating, consistent with previous work (Drew, Murray et al. 2003), that AMF hyphal morphology is not significantly dependent on P availability.

SXRCT data enabled the alignment of hyphae with respect to the major axis of each hyphal compartment to be quantified. On average, this hyphal alignment angle varied between 45o and 85o. The only significant difference was that the control P+ treatment had a lower mean angle than control P- and inoculated P+ and P- treatments (Fig S28, t-test $p<0.05$). Further analysis indicated that at the fertilizer pellet location (h2), statistically significant differences were recorded in alignment of hyphae between control P+ (49o) and both P- treatments (CP- is 65o and IP- is 67o), and when pooling the data at the same location, hyphae in P+ treatments were found to be statistically more aligned to the compartment axis (51o) than in P- treatments (66o). One way of explaining these differences is via the context of cost-benefit of the symbiosis, i.e., when the supply of P is comparatively more scarce, the hyphae 'search' for P by deviating more from the primary growth direction as defined by the geometry of the growth assay.

The SXRCT data also allowed analysis of the (normalised) tortuosity of hyphal branches. Means of tortuosity in the control P+ and P- treatments were not significantly different (t-test, $p<0.05$), but the distributions were significantly different (KS, $p<0.05$). This could be explained by the larger variation seen in CP+ (see Fig 3c). The opposite was found for inoculated treatments, where P+ displayed a significantly different/wider (normalised) hyphal tortuosity distribution (KS, $p<0.05$) than P-. However, when comparing the inoculated and control P+ treatments, the inoculated P+ treatment had a significantly different/wider tortuosity distribution than control P+ treatment, supporting the hypothesis that AMF fungi might be searching out P sources more aggressively than the non-mycorrhizal strains present in the control samples.

# 6 Destructive hyphal microscopy

A drawback of the SXRCT method for measuring hyphal density is that hyphae are only visible in the air-filled pore spaces. It is not possible from these data to infer what proportion of total hyphal density might be occluded by other soil phases (particularly the mixed phase). As a secondary measure of hyphal density, the method of (Jakobsen, Abbott et al. 1992) was used to measure total hyphal content. Replicate hyphal compartments were prepared for analysis in this manner, using inoculated and control conditions as before.

## 6.1 Preparation of slides

Using a scalpel, tubes were cut into three sections: near-root, intermediate and distant, taken as the first, second and third 0.3 ml volumes respectively. Using forceps, the soil was gently pushed out into a weighing boat and the mass immediately determined.
Each 0.3 ml soil volume was mixed with 100 ml of DI water, and macerated at high power for 30 s in a blender (38BL40 Waring). The blended suspension was immediately transferred to a 200 ml Schott bottle.

Each bottle was vigorously shaken by hand for 10 s, and allowed to settle for 60 s. Two 5 ml aliquots of fluid were pipetted from a depth of 30 mm below the fluid surface, and each transferred to a separate column of a filtration manifold (DHI). At the base of each filtration column, a 1.2 µm nitrocellulose filter paper (Millipore) of 21 mm diameter (filterable area) had been placed. A vacuum was applied to the filtration manifold, drawing the supernatant through the filters. 3 ml of staining solution (acidified glycerol with 0.1% trypan blue) was added to each column and allowed to stand for 8 minutes. The stain was then drawn off with the vacuum, and two rinses of DI water (5 ml) drawn through the filter. The filters were then gently removed from their holders and transferred to labelled slides. After 4 h of air drying, the filters were mounted on standard slides using 4 drops of immersion oil and

glass cover slips. This protocol produced two filters for each 0.3 ml sample of soil, each containing the length of hyphae extracted from a 5 ml aliquot of the blended solution.

### *6.2 Hyphal counting*

Counting of hyphae on filters was carried out using digitised images collected via light microscopy. An Olympus BX41 microscope was used, with a 20x objective magnification and bright-field illumination (transmission). A total of 20 images was acquired in randomised locations across each filter. This produced a total of 40 images for each soil sample, once replicate filters are accounted for. A total of 4400 images were acquired in this manner for all experimental conditions.

Prior to counting, these images were examined to assess the phenotypic diversity within the entire sample set (Figure S1.29). Based on these classifications, a set of candidate phenotypes was defined, into which all hyphal structures were subsequently placed during the counting stage. These were devised in discussion with Professor Iver Jakobsen at the University of Copenhagen.

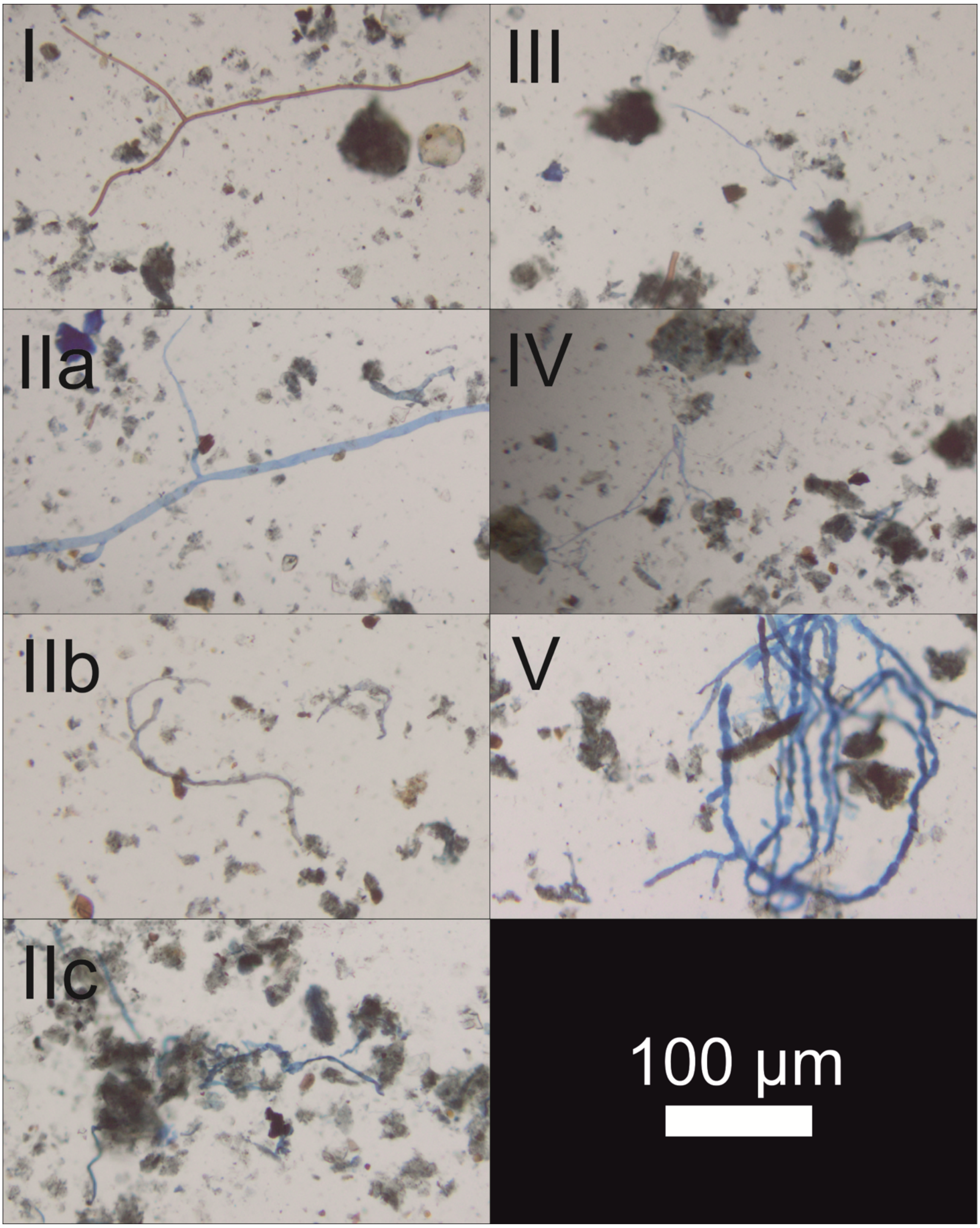

*Figure S1.29 – Examples of the phenotypes established for hyphal counting.*

Type I was defined as melanised, with no evidence of staining and smooth walls. Type IIa was defined as stained with smooth walls and no evidence of internal structure. Type IIb was defined as stained with knobby walls. Type IIc was defined as stained, very knobby, and with internal structure visible. Type III was defined as stained and very fine. Type IV was similar to III, but less fine diameter. Type V was highly stained, smooth walled but with a 'string of pearls' morphology, almost always occurring in large and tangled clumps. Type VI (not shown) included any definitively non-hyphal filamentous structures (including nematodes).

Counting of hyphae was conducted according to the line intersect method (Tennant 1975, Giovanetti & Mosse, 1980). This allows the estimation of hyphal length per unit mass of

soil via a linear relationship with the number of grid intersections for filtered hyphae on a microscope slide. A script written in FIJI aided this process, producing 10 x 10 grids of 50 um spacing, which were defined in randomised locations within each image to minimise user bias. Counts were made by a user, according to the phenotype descriptions, and using a counting script in FIJI, which tabulated and automatically saved the counts of hyphal intersections for each phenotype.

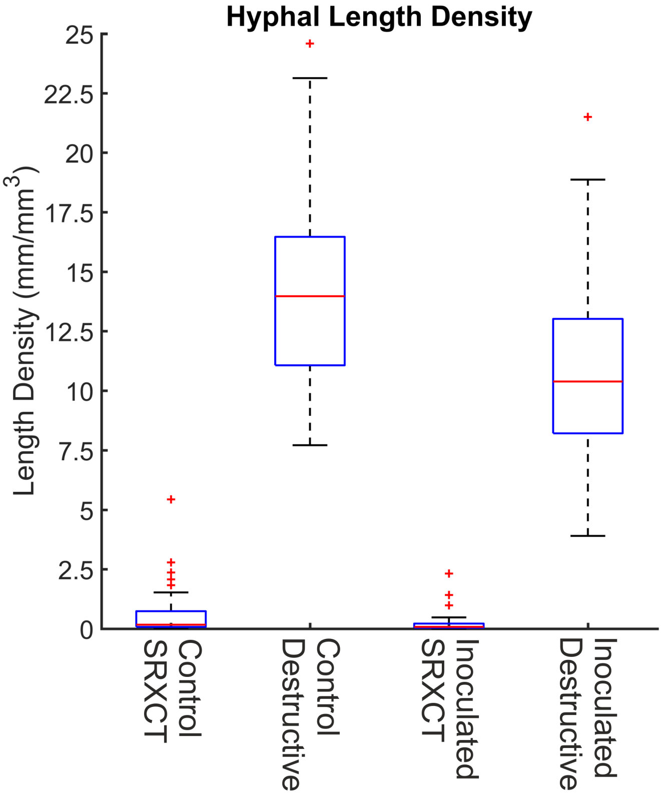


Figure S1.30: Comparison of length density quantification between the SRXCT (pore pace only) hyphal fraction, and the Jakobsen microscopy (all) hyphal fraction ; red line shows the mean length density, blue box describes the inter quartile range i.e. 25%-75% of data falls within this interval, red dots are the outliers and black whiskers show plus/minus ±2.7$\boldsymbol{\sigma}$ where $\boldsymbol{\sigma}$ is the standard deviation;

# *7 Genetic analysis of fungal colonisation*

PCR of soil dsDNA was carried out to validate the colonisation of hyphal compartments by mycorrhizae under the experimental conditions used. The entire growth experiment was re-run to produce a set of inoculated and control samples prepared under identical conditions to the SXRCT experiment.

At harvest, three replicates of $D_1$ chambers at 2 and 4 week growth periods in P+ and P- (inoculated and control) were snap-frozen in liquid nitrogen and stored at -80°C until further analysis. Samples were transported to the Department of Plant Sciences, Oxford, UK, to conduct genetic extractions and PCR.

## *7.1 Extraction of soil dsDNA*

Samples were thawed to room temperature and 0.5 g of soil (wet weight) extracted from the uppermost part of each hyphal compartment. DNA was extracted using a Zymo D6003

soil dsDNA extraction kit (Zymo Research, Orange, CA, USA) following the protocol provided. Samples were kept cooled on crushed ice during the process. Four samples of raw, dry soil were also prepared from the *fine* fraction stock used to prepare hyphal compartments. A sample of raw *Rhizophagus irrecularis* inoculum paste was also prepared for analysis.

## *7.2 Quantification of total dsDNA*

The concentration (ng/µL) of dsDNA in the extractant solutions was quantified using a Qubit 2.0 fluorometer and QUBIT BR assay (Life Technologies, Invitrogen, Darmstadt, Germany). This allowed the relative amounts of dsDNA (i.e. all) to be compared among the samples, and the samples homogenised to have an identical starting mass of dsDNA for PCR.

## *7.3 PCR*

Polymerase chain reaction (PCR) analysis of soil double stranded DNA (dsDNA) was carried out to validate the colonisation of hyphal compartments by AMF under the experimental conditions used. At 2 and 4 weeks, samples from the top-most hyphal compartments were taken from three replicates of the four treatments (inoculated/control x +P/-P), snap-frozen in liquid nitrogen and stored at -80$^{o}$C until further analysis. Samples were thawed to room temperature, and DNA extracted from 0.5 g of soil (wet weight) from each hyphal compartment, proximal to the root compartment, using a Zymo D6003 dsDNA extraction kit (Zymo Research, Orange, CA, USA), following the manufacturer protocol. Four control samples *fine* fraction soil stock, and samples of *Rhizophagus irregularis* inoculum paste were also prepared for analysis using the approach set out further herein. The concentration (ng/µL) of dsDNA in extractant solutions was quantified using a Qubit 2.0 fluorometer and QUBIT BR assay (Life Technologies, Invitrogen, Darmstadt, Germany). This allowed the relative amounts of dsDNA (*i.e.* all) to be compared among the samples, and the samples were homogenised to have an identical starting mass of dsDNA for PCR. Before PCR, the initial quantity of dsDNA in each sample was standardised to 5 ng per reaction in all cases. Four sets of primers were used: (a) AMF primers (Kruger, Stockinger et al. 2009) designed to amplify Glomeromycota fungi via a specific internal transcribed spacer (ITS) fragment binding to the end of 18S (SSUmAf 5-TGGTGAATCTTRTGAAACTT-3) and the beginning of 28S (LSUmBr 5-DAACACTCGCAYAYATGYTAGA-3). Kruger's primers were tested to be specific to all AMF fungi, but they have a high similarity (1 and 2 nucleotides dissimilarity for the forward and reverse primer, respectively) to "lower" plants as mosses, liverworts and hornworts. Moreover, there are non-AMF fungal species with essentially identical sequence in their ribosomal genes (Kruger, Stockinger et al. 2009). Hence, Kruger's primers amplify AMF fungi but also potentially other fungi and "lower" plant species DNA. (b) a bespoke set of AMF ITS primers (called F_28Smyc 5-TYGTYRAAAGGGAAAYGRTT-3 and R_18Smyc 5-GATGYRAGARCCAAGAGATC-3 in this study and referred as *Tkacz* primers). These primers were designed using an alignment of all Glomeromycota ITS genomic fragments available at the NCBI Genbank nr/nt database (as of April 2017) that contain the binding sites for the Kruger set of primers. The new primers were used in a combination with the (Kruger, Stockinger et al. 2009) primers as F_28myc-LSUmBr and SSUmAf2-R_18Smyc to produce two ~400 bp fragments (depending on the fungal species). The new set of primers were tested *in silico* to validate they did not match any other non-Glomeromycota fungi and tested *in vitro* to confirm PCR amplification of the *R. irregularis* inoculum sample. These primers were designed specifically for *R. irregularis* and *in-silico* tested to be at least 1 nucleotide different (for each primer) against any other fungal or plant DNA. Moreover, these primers were tested against bulk soil samples producing negative results.

Bulk soil is unlikely to contain AMF DNA but certain to have other fungal, plant and bacterial DNA. The last two primer sets were: (c) a broad range primer (Buee, De Boer et al. 2009, Buee, Reich et al. 2009), designed to amplify Asco- and Basidio- rather than Glomoro-mycota, and (d) bacterial primers (515F and 806R targeting prokaryotic 16S rRNA V4 region (Caporaso, Lauber et al. 2011)). All these primers were first tested against bulk soil (negative control) and *R. irregularis* DNA (inoculum paste as positive control). During test PCR run, primers a) Kruger and b) Tkacz produced expected size products for positive control, but not for the negative control, while primers c) Buee ITS and d) bacterial 16S produces bands in negative control, but not for the positive control. Hence, primers a) and b) target AMF fungi as *R. irregularis*, while primers c) and d) target other fungal and non-fungal species, but not AMF fungi.

For all primer sets, standard PCR conditions were used: 98°C for 3 min, 35 cycles of 98°C for 15 sec, 55°C for 30 sec and 72°C for 100 sec, followed by a final elongation step at 72°C for 7 min using Phusion high-fidelity polymerase (NEB M0530L) and a PCR master mix with GC buffer (NEB M0532L). Following PCR, electrophoresis gels were run to determine whether amplification had been successful.

## 7.4 Microbial Diverstiy Analysis

Microbial diversity analysis methods are presented in full in the main text of the paper. However, below we show the 18S sequencing results for the abundance of difference species.

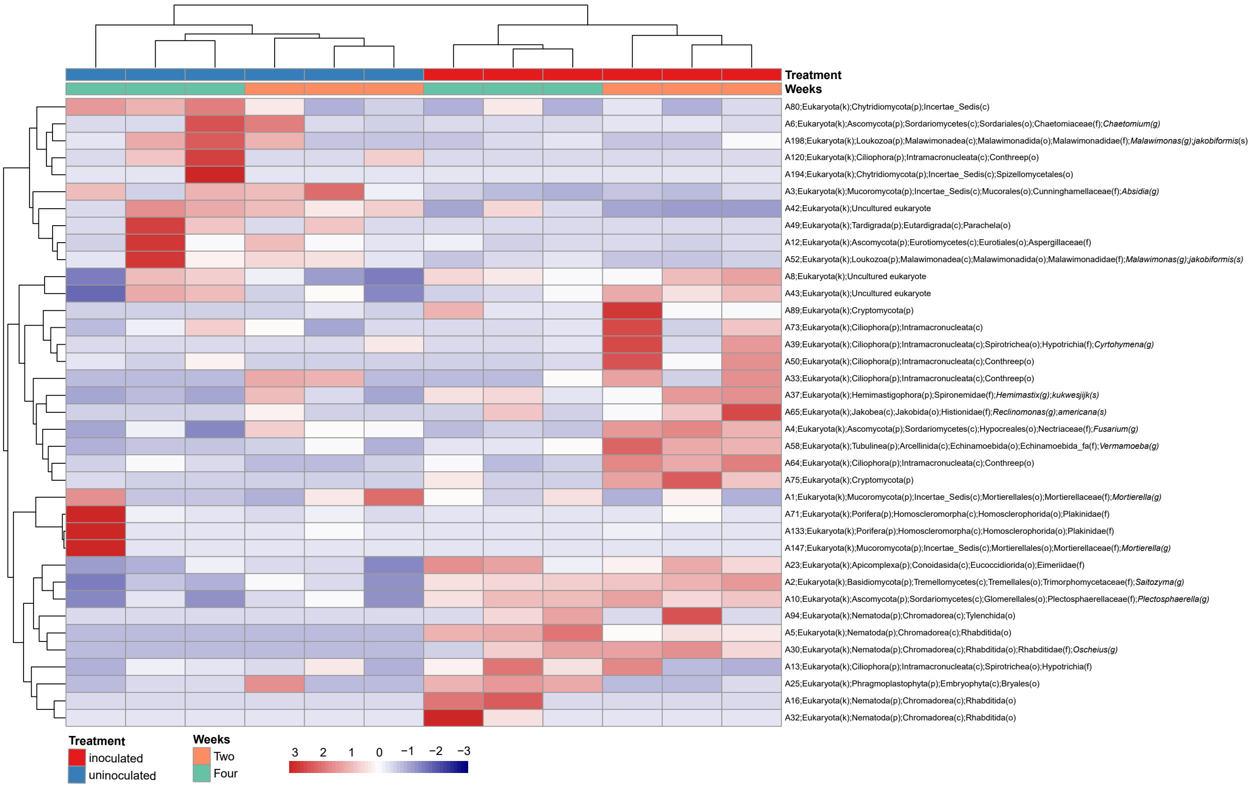


*Figure S1.31: 18S sequencing results showing abundance of different species*

# 8 Chemical analysis of samples

Following SXRCT, intact samples were prepared for chemical analysis using a range of techniques. To aid resin embedding of samples, chambers were designed to allow hyphal compartments to be aligned at known 3D orientation for sectioning (Figure S1.30). These were produced from laser-cut 3 mm acrylic.

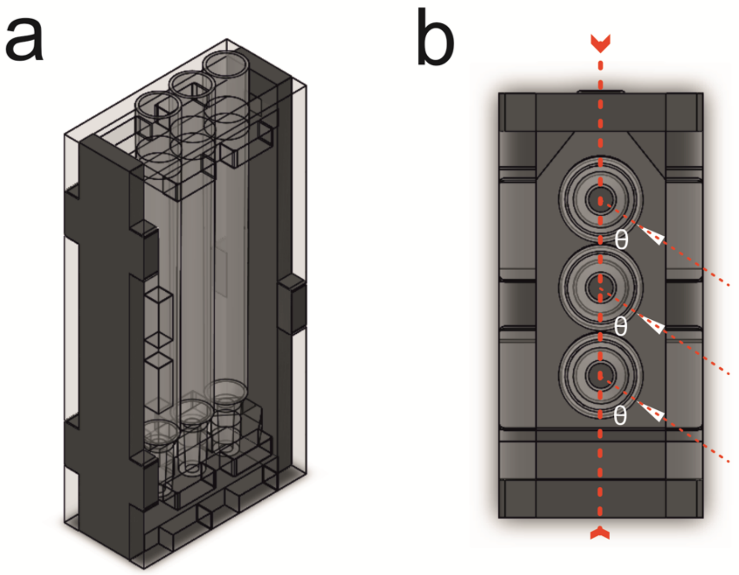


*Figure S1.32 – (a) The boxes used to embed samples in epoxy resin in sets of 3 (one box per slide). The chambers are aligned with notches engraved with the same angular offset used for imaging (θ), such that the cut plane (shown - orthogonal to the short axis of the box) transected the beam axis, aligning the cut plane with one of the three principal planes of the SXRCT reference system. The system height x width x thickness = 68.75 x 33.73 x 16.5 mm.*

## 8.1 Resin embedding

The samples were first dipped in liquid nitrogen (-80°C) and then freeze dried for 24 hours. Freeze drying was chosen as to conserve as far as possible the organic components of the soil. The samples were then placed in custom resin perfusion boxes / embedding moulds (Figure S1.32) and oriented to the starting orientation used for SRXCT imaging of the respective sample, to aid later registration of thin sectioned surfaces to planes through the SRXCT image volumes.

The embedding medium for this study was epoxy resin (EpoTek 301). This epoxy resin has a low viscosity, high purity and resilience against X-rays. The epoxy resin was diluted with 10% (w/w) ethanol as to slow curing and decrease viscosity. Slowing down the curing improved total impregnation / infusion and decreased the possibility of a rapid increase in temperature. After infusion of resin, the samples were cured for 48 hours after which they were cut and polished.

Cutting was applied length wise to the syringe tubes using a Metacome T210 Presi saw (https://www.presi.com/en/product/mecatome-t210/) with a diamond blade from MetPrep (https://metprep.co.uk/wp-content/uploads/2016/03/TDS0019-Metprep-Diamond-CR-Wafering-blades.pdf). Finally, the samples were polished with 9, 6 and 1 μm diamond paste. After the samples were polished and no visible scratches were visible, the samples were cleaned with ethanol. In order to minimise artefacts for XRF imaging, such as distance differences, each corner of the sample block was measured and adjusted in

height using spacers. After adjustment, the final height difference of each corner was within 1 µm of planar.

## 8.2 SR-XRF imaging

Synchrotron X-ray Fluorescense imaging (XRF) was applied on the samples, since this technique has a higher sensitivity compared to other soil chemistry methods (SEM-EDX) and allows to detect changes in speciation. XRF was conducted at beamline I18 at Diamond Light Source (DLS; Didcot, UK) and at beamline PHOENIX of the Swiss Lightsource (SLS; Villigen, Switzerland). Elemental maps for light elements were collected at an incident beam energy of 2.7 keV, i.e., lower energy than Cl edge as to get rid of the Cl peak present in the Epoxy resin.

Soil thin sections were mounted on an x-y-z stage and rastered relative to the fixed incident beam (see Figure S1.31). At DLS the maps were recorded under a He atmosphere, while at SLS the maps were recorded under vacuum (~$10^{-6}$ mbar). Both beamlines utilized Kirkpatrick-Baez mirrors to produce a spot size of 10 and 5 (DLS), and 2.5 µm (SLS) respectively. In addition, a Si(111) monochromator was used to select the incident beam energy. X-rays were detected using a four element (DLS) and a single element (SLS) Ketek GmbH detector. X-ray flux was estimated to be $10^{10}$-$10^{11}$ photons $sec^{-1}$. Finally, at both beamlines a full Energy Dispersive Spectrum (EDS) was recorded for each pixel of the XRF map. Each map was quantified from fundamental parameters, obtained from fitting the XRF spectra obtained from respectively a spessartine garnet and Durango apatite with known concentrations (See table S1.2 and S1.3).

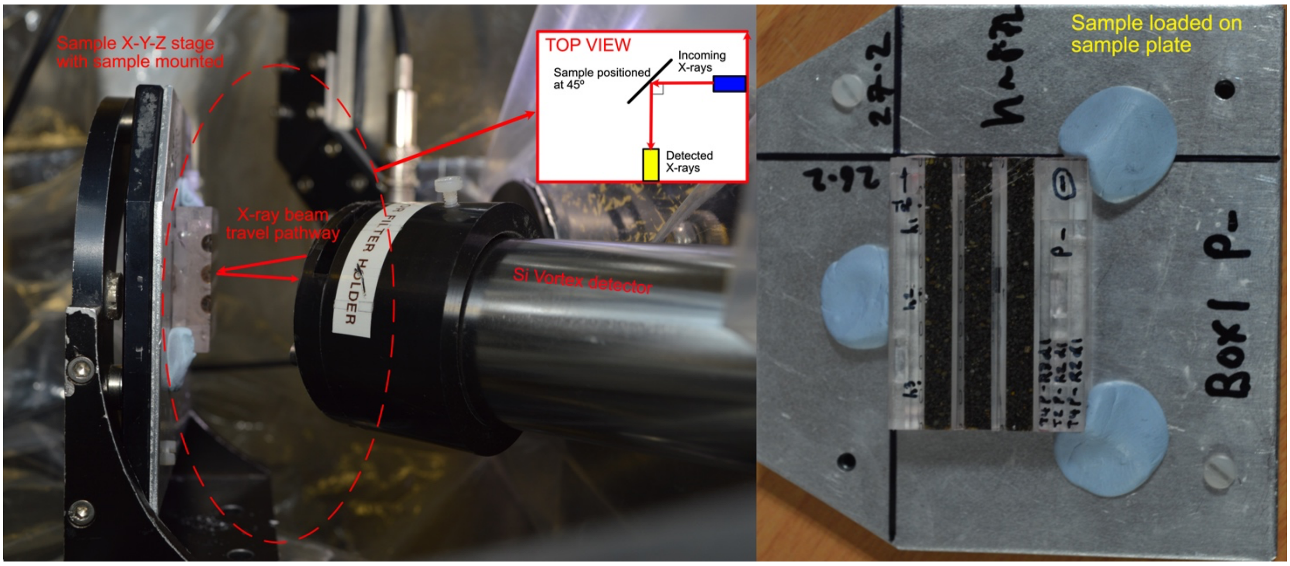


*Figure S1.33 – Sample mounted for imaging at I18. Sample is mounted on a x-y-z sample stage and positioned at 45° relative to the incoming X-rays. A Si Vortex detector is placed at a hard angle to the X-rays to minimize scatter. The samples surface was mounted precisely parallel to the sample plate holder surface to prevent geometry artefacts for P and S.*

X-ray Absorption Near-Edge Structure (XANES) spectroscopy was performed on the phosphorus and sulfur K-edges. The monochromator edge position was calibrated using an apatite standard and a Zn-sulfate standard. In order to constrain the speciation of P and S in the soil samples, a series of standard spectra (see Figure 9 in the main text) were collected. Background subtraction, data normalization and fitting were performed using *Athena* and *Artemis* routines (Ravel and Newville 2005) from the Demeter software package.

*Table S1.2. SR-XRF quantification of Spessartine garnet.*

| | Benchtop XRF | | Bulk XRF | | LA-ICPMS | | ICP-MS digestion | | SR-XRF | |
|---|---|---|---|---|---|---|---|---|---|---|
| Element | w/w%* | stdev | w/w% | stdev | w/w%/ppm | stdev | w/w%/ppm | stdev | w/w%/ppm | stdev |
| Na | | | | | 0.058 | 0.008 | | | | |
| Mg | | | | | 0.002 | 0.000 | | | | |
| Al | 8.720 | 0.124 | 10.552 | 0.053 | 11.460 | 0.516 | 10.460* | 0.045 | 10.010 | 0.053 |
| Si | 14.059 | 0.105 | 17.189 | 0.086 | 12.792* | 0.116 | | | 15.140 | 0.035 |
| P | 0.216 | 0.026 | 0.103 | 0.001 | 980.370 | 0.005 | 978.400 | 9.676 | 710.000 | 3.800 |
| K | 0.029 | 0.007 | | | | | | | | |
| Ca | 0.190 | 0.010 | 0.152 | 0.001 | 0.140* | 0.006 | | | | |
| Ti | 0.100 | 0.018 | 0.051 | 0.000 | 640.819 | 57.599 | 724.000 | 5.915 | | |
| V | | | 0.002 | 0.001 | 0.019 | 0.028 | 0.949 | 0.046 | | |
| Mn | 34.922 | 0.020 | 27.320 | 0.137 | 26.631* | 0.171 | 26.800* | 0.115 | | |
| Fe | 5.451 | 0.153 | 3.744 | 0.019 | | | | | | |
| Zn | 0.085 | 0.006 | 0.015 | 0.000 | 250.922 | 10.540 | 209.000 | 0.968 | | |
| Ga | 0.060 | 0.008 | | | 63.765 | 1.988 | 52.110 | 0.356 | | |
| As | 0.026 | 0.016 | | | 25.016 | 3.986 | 1.748 | 0.097 | | |
| Sr | 0.048 | 0.007 | 0.002 | 0.000 | 0.010 | 0.012 | 5.772 | 0.096 | | |
| Zr | 0.003 | | 0.003 | 0.000 | 18.224 | 1.078 | 44.130 | 11.522 | | |
| Pb | 0.045 | 0.040 | | | 0.014 | 0.022 | 0.739 | 0.007 | | |
| Ba | | | | | 0.006 | 0.022 | 8.926 | 0.108 | | |
| Y | 0.058 | 0.008 | | | 99.199 | 5.610 | 158.200 | 0.375 | | |

*w/w%, 100% = 1,000,000 ppm. Blue shading used for quantification

*Table S1.3. SR-XRF quantification of Durango apatite.*

| | Benchtop XRF | | Bulk XRF | | LA-ICPMS | | ICP-MS digestion | | SR-XRF | |
|---|---|---|---|---|---|---|---|---|---|---|
| Element | w/w%* | stdev | w/w% | stdev | w/w%/ppm | stdev | w/w%/ppm | stdev | w/w%/ppm | stdev |
| Na | | | 0.131 | | 0.116 | 0.006 | | | | |
| Mg | | | | | 0.078 | 0.004 | | | | |
| Al | 0.147 | 0.027 | | | 0.476 | 0.000 | 277.000 | 1.795 | 0.260* | 0.050 |
| Si | 0.334 | 0.023 | 0.350 | 0.002 | 0.268* | 0.013 | | | 0.440* | 0.081 |
| P | 20.897 | 0.078 | 17.454 | 0.087 | 22.638* | 1.145 | 19.500* | 0.063 | 20.810* | 2.240 |
| K | 0.075 | 0.007 | | | | | | | | |
| Ca | 35.734 | 0.121 | 38.831 | 0.194 | 36.355* | 0.507 | | | | |
| Ti | 0.036 | 0.004 | | | 24.451 | 4.571 | 218.800 | 1.982 | | |
| V | | | 0.002 | 0.000 | 32.815 | 2.867 | 30.160 | 0.072 | | |
| Mn | 0.028 | 0.004 | 0.017 | 0.000 | 95.240 | 6.020 | 99.150 | 0.310 | | |
| Fe | 0.207 | 0.008 | 0.020 | 0.000 | | | | | | |
| Zn | 0.013 | 0.004 | 0.008 | 0.000 | 1.379 | 4.751 | 0.629 | 0.066 | | |
| Ga | 0.077 | 0.005 | | | 0.235 | 0.052 | 0.531 | 0.020 | | |
| As | 0.114 | 0.021 | | | 1052.400 | 110.599 | 964.400 | 8.053 | | |
| Sr | 0.093 | 0.006 | 0.052 | 0.000 | 482.538 | 10.306 | 485.800 | 3.435 | | |
| Zr | | | | | 1.198 | 0.196 | 2.217 | 0.318 | | |
| Pb | 0.018 | 0.061 | | | 0.964 | 0.108 | 0.977 | 0.021 | | |
| Ba | | | 0.360 | 0.002 | 1.960 | 0.197 | 5.934 | 0.037 | | |
| Y | 0.160 | 0.006 | | | 908.595 | 63.705 | 1202.000 | 7.296 | | |

*w/w%, 100% = 1,000,000 ppm. Blue shading used for quantification

## 8.3 Image analysis

All image processing and analysis steps were performed in ImageJ and Matlab 2017b (The MathWorks Inc., Cambridge, UK). Since the biogeochemical processes of specific interest take place in the organic/loamy/clay(i.e. *mixed*) phases, the primary minerals (quartz) were masked out. For this, a global threshold was applied manually. Fertilizer pellets were masked out manually, by carefully drawing a polyline over the pellet boundary. Finally, pore space was masked out by manual thresholding using the fitted Si-maps. A 2D Euclidean distance transform was applied to the segmented image to determine the distance of any soil pixel to the plant chamber. Pixel intensity (concentration) changes with distance from the plant chamber were recorded for each elemental map. These values were plotted to determine gradients.

Finally, P and S in the segmented mixed phase were plotted. After finding the XRF slice position in the corresponding SXRCT volume associated with the same physical sample, a 3D distance transform from segmented XRF image was applied to the segmented hyphal image. For each pixel the distance to the closest hyphal surface was then calculated.

## *8.4 XANES LCF results*

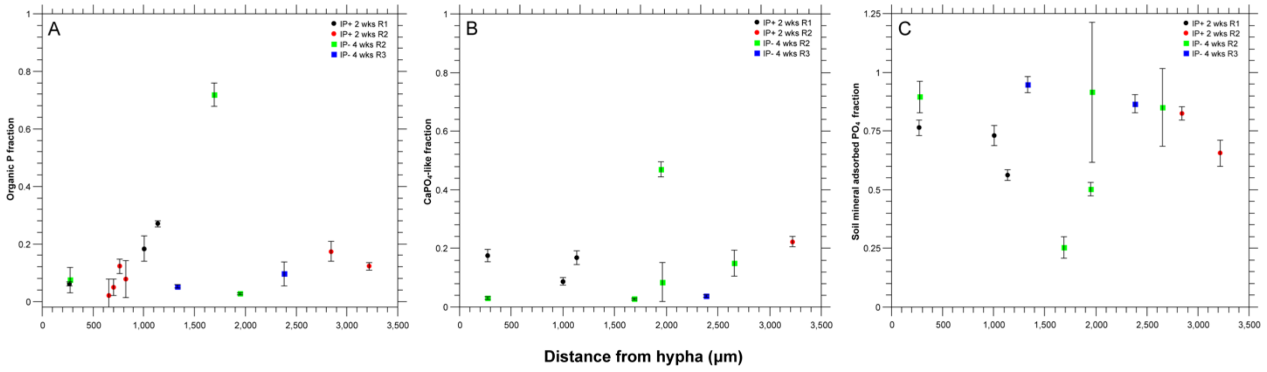


*Figure 1.34 Linear combination fit results of the different treatments: A – organic associated P; B – $CaPO_4$-like; C - soil mineral associated $PO_4$. The linear combination fits reveal no significant relationship between P-speciation and distance from a hypha. This is expected as phosphorus is extremely fixating. Errror bars represent standard deviation. N = 3.*

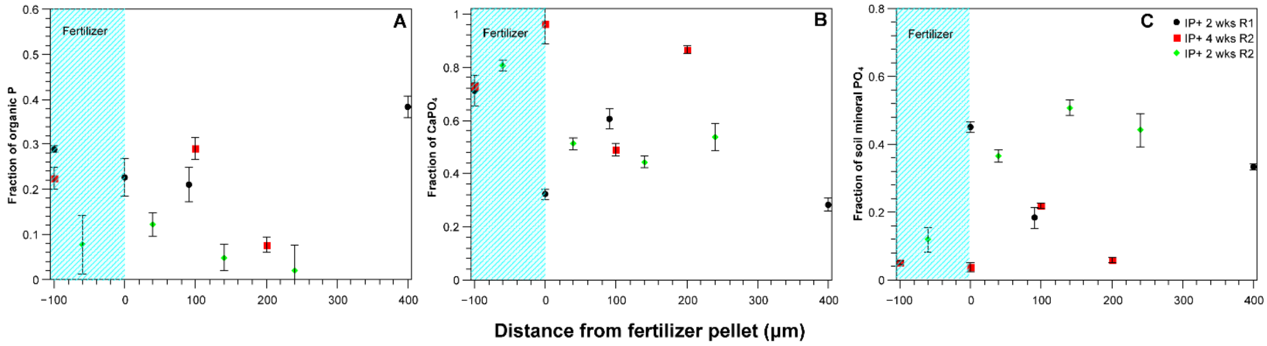


*Figure S1.35 Linear combination fit results for different phosphorus species: A – organic associated P; B – $CaPO_4$-like; C - soil mineral associated $PO_4$. The linear combination fits reveal the extreme fixating nature of P. This is expected as phosphorus is extremely fixating. The dissolved P gets fixed to organic matter and soil mineral surfaces immediately when it gets released from the fertilizer. This can be seen by looking at B, where there is a small decreasing trend in $CaPO_4$-like P with increasing distance from the fertilizer pellet. Error bars represent standard deviation. N = 3. Blue vertical bar depicts the areal within the fertilizer pellet*

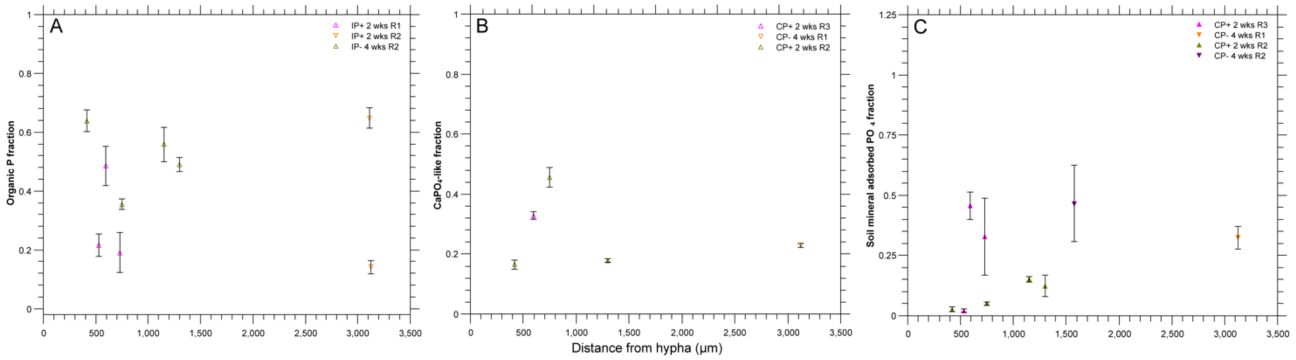


*Figure S1.36 Linear combination fit results for the controls: A – organic associated P; B – $CaPO_4$-like P; C - soil mineral associated $PO_4$. The linear combination fits reveal no significant relationship between P-speciation and distance from a hypha. This is not surprising as phosphorus is very strongly bound to soil minerals and with the absence of plant roots, no trends in speciation are expected to be observed. Moreover, the absence of plants in these samples meant that no mutualism between mycorrhiza and plant roots could be established. Error bars represent standard deviation. N = 3.*

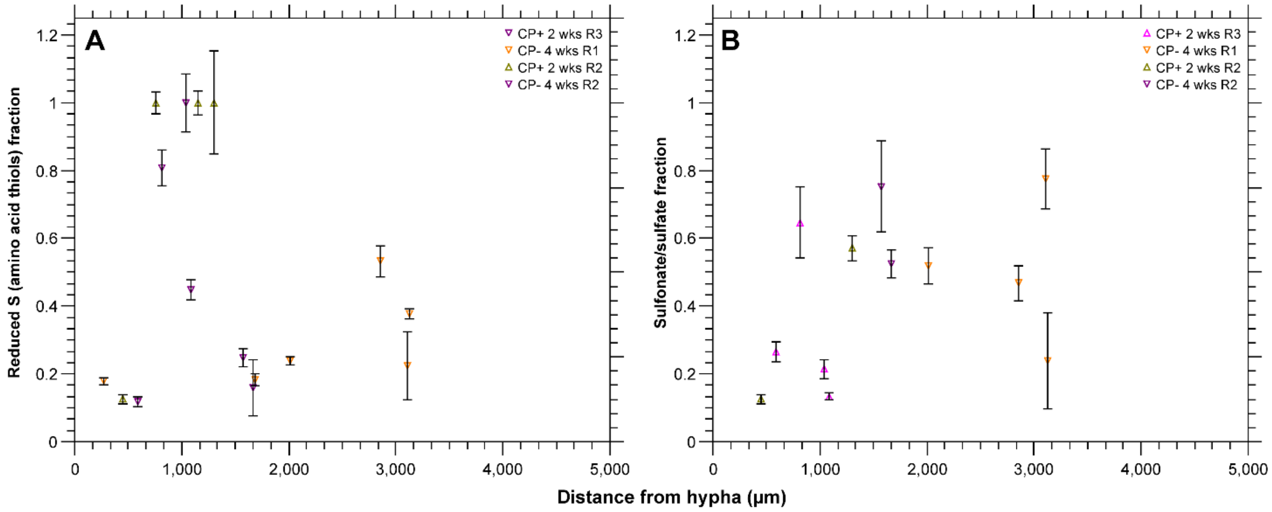


*Figure S1.37 Linear combination fit results for the controls: A – reduced S (amino acid thiols) fraction; B – sulfonate/sulfate fraction. The linear combination fits reveal no significant relationship between S-speciation and distance from a hypha. This is expected as there is no influence from a plant and mycorrhiza. The small changes (see cluster in A) are due to microbial activity and some other strains of fungi naturally occurring in these soils. Error bars represent standard deviation. N = 3. CP+ -- control with fertilizer, CP- -- control no fertilizer; R1, R2, R3 refers to the original sample replicate number.*

### *Root colonization microscopy*

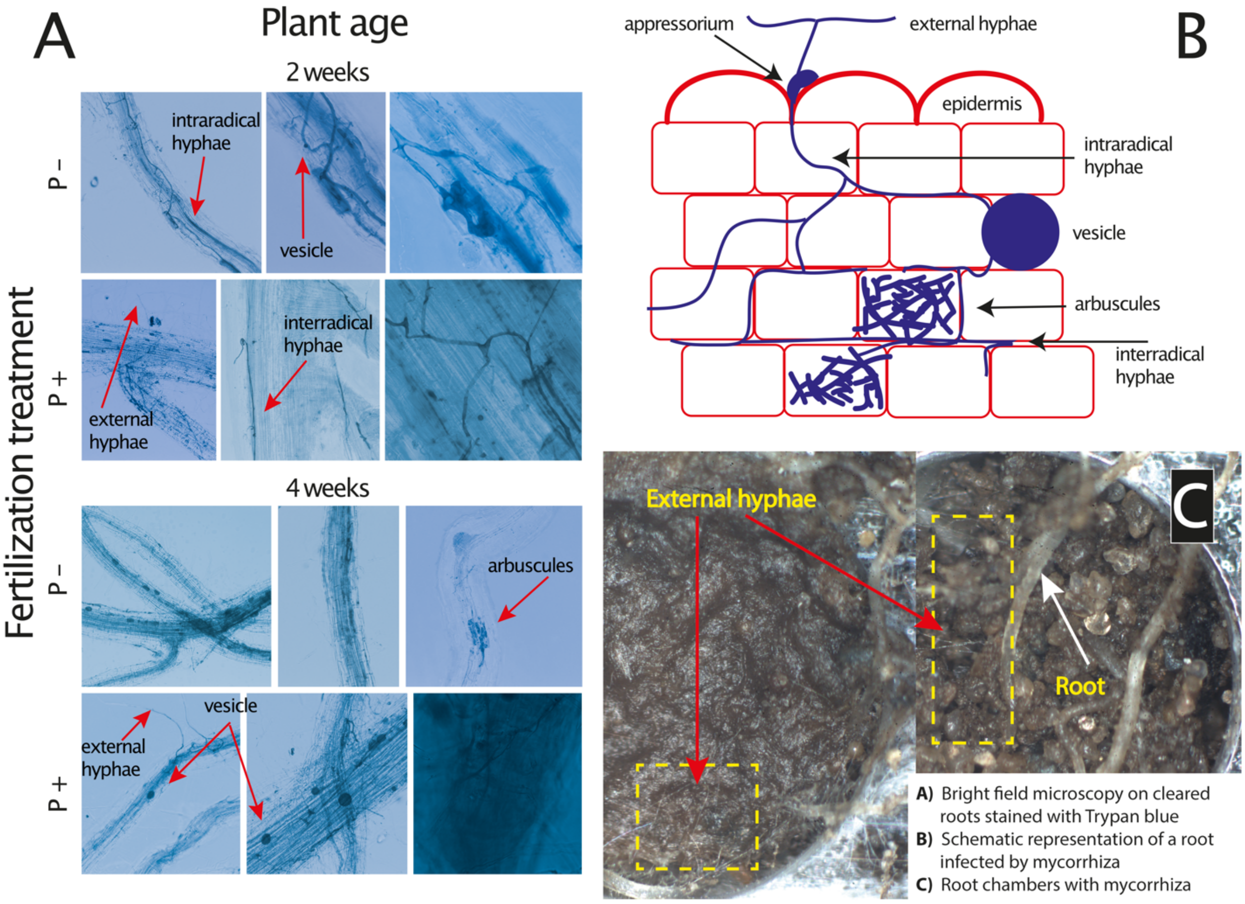


*Figure S1.38 Microscopy images of (A) roots stained with Trypan blue. In both the two- and four-weeks infection of the roots was observed. (B) Schematic overview of the different components of the mycorrhizal hyphal structures within the root. (C) External hyphae images. Images of were taken at the area where the hyphal compartments were connected to the root compartment. In nearly all root compartments connections, roots were visible.*

## 1.4. References


Buee, M., W. De Boer, F. Martin, L. van Overbeek and E. Jurkevitch (2009). "The rhizosphere zoo: An overview of plant-associated communities of microorganisms, including phages, bacteria, archaea, and fungi, and of some of their structuring factors." Plant and Soil **321**(1-2): 189-212.

Buee, M., M. Reich, C. Murat, E. Morin, R. H. Nilsson, S. Uroz and F. Martin (2009). "454 Pyrosequencing analyses of forest soils reveal an unexpectedly high fungal diversity." New Phytologist **184**(2): 449-456.

Caporaso, J. G., C. L. Lauber, W. A. Walters, D. Berg-Lyons, C. A. Lozupone, P. J. Turnbaugh, N. Fierer and R. Knight (2011). "Global patterns of 16S rRNA diversity at a depth of millions of sequences per sample." Proceedings of the National Academy of Sciences of the United States of America **108**: 4516-4522.

Daly, K. R., S. D. Keyes, S. Masum and T. Roose (2015). "Image based modeling of nutrient movement in and around the rhizosphere. Journal of Experimental Botany (In Press)." Journal of Experimental Botany (In Press).

Daly, K. R., S. J. Mooney, M. J. Bennett, N. M. J. Crout, T. Roose and S. R. Tracy (2015). "Assessing the influence of the rhizosphere on soil hydraulic properties using X-ray

computed tomography and numerical modelling." Journal of Experimental Botany **66**(8): 2305-2314.
Doube, M., M. M. Kłosowski, I. Arganda-Carreras, F. Cordeliéres, R. P. Dougherty, J. Jackson, B. Schmid, J. R. Hutchinson and S. J. Shefelbine (2010). "BoneJ: free and extensible bone image analysis in ImageJ." Bone **47**: 1076-1079.
Doube, M., M. M. Kłosowski, I. Arganda-Carreras, F. P. Cordelieres, R. P. Dougherty, J. S. Jackson, B. Schmid, J. R. Hutchinson and S. J. Shefelbine (2010). "BoneJ: free and extensible bone image analysis in ImageJ." Bone **47**: 1076–1079.
Drew, E. A., R. S. Murray, S. E. Smith and I. Jakobsen (2003). "Beyond the rhizosphere: growth and function of adbuscular mycorrhizal external hyphae in sands of varying pore sizes." Plant and Soil **251**: 105-114.
Drew, E. A., R. S. Murray, S. E. Smith and I. Jakobsen (2003). "Beyond the rhizosphere: growth and function of arbuscular mycorrhizal external hyphae in sands of varying pore sizes." Plant and Soil **251**(1): 105-114.
Gregory, P. J., D. J. Hutchison, D. B. Read, P. M. Jenneson, W. B. Gilboy and E. J. Morton (2003). "Non-invasive imaging of roots with high resolution X-ray micro-tomography." Plant and Soil **255**(1): 351-359.
Jakobsen, I., L. K. Abbott and A. D. Robson (1992). "External Hyphae of Vesicular Arbuscular Mycorrhizal Fungi Associated with Trifolium-Subterraneum L .2. Hyphal Transport of P-32 over Defined Distances." New Phytologist **120**(4): 509-516.
Keyes, S. D., R. P. Boardman, A. Marchant, T. Roose and I. Sinclair (2013). "A robust approach for determination of the macro-porous volume fraction of soils with X-ray computed tomography and an image processing protocol." European Journal of Soil Science **64**(3): 298-307.
Keyes, S. D., L. Cooper, S. Duncan, N. Koebernick, D. M. M. Fletcher, C. P. Scotson, A. van Veelen, I. Sinclair and T. Roose (2017). "Measurement of micro-scale soil deformation around roots using four-dimensional synchrotron tomography and image correlation." Journal of the Royal Society Interface **14**(136).
Koebernick, N., K. R. Daly, S. D. Keyes, T. S. George, L. K. Brown, A. Raffan, L. J. Cooper, M. Naveed, A. G. Bengough, I. Sinclair, P. D. Hallett and T. Roose (2017). "High-resolution synchrotron imaging shows that root hairs influence rhizosphere soil structure formation." New Phytologist **216**(1): 124-135.
Kruger, M., H. Stockinger, C. Kruger and A. Schussler (2009). "DNA-based species level detection of Glomeromycota: one PCR primer set for all arbuscular mycorrhizal fungi." New Phytologist **183**(1): 212-223.
Legland, D., I. Arganda-Carreras and P. Andrey (2016). "MorphoLibJ: integrated library and plugins for mathematical morphology with ImageJ." Bioinformatics **32**(22): 3532-3534.
Lucas, S. D. and D. L. Jones (2006). "Biodegradation of estrone and 17 beta-estradiol in grassland soils amended with animal wastes." Soil Biology & Biochemistry **38**(9): 2803-2815.
Marone, F. and M. Stampanoni (2012). "Regridding reconstruction algorithm for real-time tomographic imaging." J. Synchrotron Radiat **19**: 1029–1037.
Ravel, B. and M. Newville (2005). "ATHENA, ARTEMIS, HEPHAESTUS: data analysis for X-ray absorption

502 spectroscopy using IFEFFI." Journal of Synchrotron Radiation.
Schweiger, P. and I. Jakobsen (2000). "Laboratory and field methods for measurement of hyphal uptake of nutrients in soil." Plant and Soil **226**(2): 237-244.
