# Supplementary material for: Multimodal correlative imaging and modelling of phosphorus uptake from soil by hyphae of mycorrhizal fungi
Source: New Phytol. 2022 Feb 15;234(2):688–703. doi: 10.1111/nph.17980 (PMC9307049; doi:10.1111/nph.17980)
Supplement: Supplementary file 2 — Methods S2 Full description of mathematical modelling. Please note: Wiley Blackwell are not responsible for the content or functionality of any Supporting Information supplied by the authors. Any queries (other than missing material) should be directed to the New Phytologist Central Office. [file NPH-234-688-s001.pdf]

Supporting Information Methods2 (SI-2): Mathematical Modelling

Article Title: Multimodal correlative imaging and modelling of phosphorus uptake from soil by hyphae of mycorrhizal fungi

Authors: Sam Keyes, Arjen van Veelen, Dan McKay Fletcher, Callum Scotson, Nico Koebernick, Chiara Petroselli, Katherine Williams, Siul Ruiz, Laura Cooper, Robbie Mayon, Simon Duncan, Marc Dumont, Iver Jakobsen, Giles Oldroyd, Andrzej Tkacz, Philip Poole, Fred Mosselmans, Camelia Borca, Thomas Huthwelker, David L. Jones, Tiina Roose

Article Acceptance Date: Jan 4th, 2022

1. Determination of hyphal length density model parameters by data fitting

Here we describe the mathematical model and its fitting procedure used to obtain estimates of the parameters to characterise hyphal growth and development based on a model by Schnepf and Roose [1] (see also Schnepf, et al. [2]). A detailed description of the model is given in Section 1.1 and the fitting procedure along with the fits are presented in Section 1.2. The model is fit to data which has been obtained by:

- Jakobsen, et al. [3] method of destructive soil test results for hyphal length density.
- Hyphal length density results obtained from Synchrotron Radiation X-ray Computed Tomography (SXRCT) analysis.

The model has three parameters that we fit to the data: v [cm s^{-1}], the rate of hyphal tip migration, b [s^{-1}], the net hyphal tip birth rate, and k [cm^{-2} s^{-1}], the constant tip flux at the root surface, *i.e.*, net root colonisation rate.

A schematic of the experimental setup from which the hyphal length densities were obtained is shown in Figure S1.2 in SI-1 together with the plant growth assay and experimental description in Suppmenetary Information SI-1.

1.1. Hyphal length density model

The system of equations describing the hyphal length density development in time and space is based on Schnepf, et al. [2] and it is given by

$$\frac{\partial n}{\partial t} = -v\frac{\partial n}{\partial x} + f, \qquad t \geq 0, x \geq 0, \qquad \text{(S2.1)}$$

$$\frac{\partial \rho}{\partial t} = n|v| - d\rho, \qquad t \geq 0, x \geq 0, \qquad \text{(S2.2)}$$

where n [cm^{-3}] is the hyphal tip density, ρ [cm cm^{-3}] is the hyphal length density, x [cm] is the distance from the root compartment, t [s] is time, v [cm s^{-1}] is the rate of tip growth/movement, f [cm^{-3} s^{-1}] is the net rate of creation/branching of hyphal tips and d [s^{-1}] is the hyphal death rate.

These equations are solved subject to the initial conditions, i.e., no hyphae present to begin with, given by

$$n = 0, \quad t = 0, \quad x \geq 0, \qquad \text{(S2.3)}$$
$$\rho = 0, \quad t = 0, \quad x \geq 0,$$

and the boundary condition given by

$$\boldsymbol{v}.\boldsymbol{e_n} n = k, \quad x = 0, \ t > 0, \qquad \text{(S2.4)}$$

where k [cm^{-2} s^{-1}] is the constant tip flux/creation at root surface and $\boldsymbol{e_n}$ is the unit normal vector to the boundary.

We assume that f can be described by a simple net birth rate $f = bn$ hence equation (S2.1) can be written

$$\frac{\partial n}{\partial t} + v\frac{\partial n}{\partial x} = bn, \qquad \text{(S2.5)}$$

where all variables are as defined above.

Model Solution

As it is a first order linear hyperbolic partial differential equation model, we solve it using the method of characteristics[4] in two regions, $x \geq vt$, which is the region of the solution influenced by the initial condition, i.e., region away from root surface, and $x \leq vt$, the region which depends on the boundary condition, i.e., region which is near to the root surface.

For the region $x \geq vt$ which depends on the initial condition the first step in finding a solution is the development of three parameteric ordinary differential equations (ODE)

$$\frac{dt}{d\tau} = 1, \quad \frac{dx}{d\tau} = v, \quad \frac{dn}{d\tau} = bn. \qquad \text{(S2.6)}$$

Using the initial condition (S2.3) we also parameterise to develop the initial Cauchy data for the region $x \geq vt$,

$$x_0(s) = s, \quad t_0(s) = 0, \quad n_0(s) = 0. \qquad \text{(S2.7)}$$

This allows for the solution to the system of ODE's given by (S2.6),

$$t = \tau,$$
$$x = v\tau + s, \qquad \text{(S2.8)}$$
$$n = n_0(s) = 0, \quad x \geq vt.$$

For the region $x \leq vt$ which depends on the boundary condition (S2.4) we use the same approach to develop the Cauchy data, i.e.,

$$x_0(s) = 0, \quad t_0(s) = s, \quad n_0(s) = \frac{k}{v}. \qquad \text{(S2.9)}$$

This gives the solution to the system of ODE's given by (S2.6) as

$$t = \tau + s,$$
$$x = \tau v, \qquad \text{(S2.10)}$$
$$n = \frac{k}{v}e^{\frac{b}{v}x}, \quad x \leq vt.$$

Combining equations (S2.8) and (S2.10), we have a piecewise solution for n,

$$n = \begin{cases} 0, & x \geq vt \\ \frac{k}{v} e^{\frac{b}{v}x}, & x \leq vt \end{cases}. \quad \text{(S2.11)}$$

We now solve the first order linear ODE given by equation (S2.2) with solution (S2.11) using the integration factor $I = e^{dt}$. Equation (S2.2) with initial condition (S2.3) then yields a solution for the hyphal length density as

$$\rho = \begin{cases} 0, & x \geq vt, \\ \frac{k}{d} e^{\frac{b}{v}x} \left(1 - e^{\frac{d}{v}(x - tv)}\right), & x \leq vt. \end{cases} \quad \text{(S2.12)}$$

When the hyphal death rate is zero, i.e., d=0, the solution is

$$\rho = \begin{cases} 0, & x \geq vt, \\ k e^{\frac{b}{v}x} \left(-\frac{1}{v}(x - tv)\right), & x \leq vt. \end{cases} \quad \text{(S2.13)}$$

1.2. Model fitting to the data

1.2.1. Fitting the model to Jakobsen destructive soil analysis data

The hyphal length densities were obtained at three sample positions along the hyphal sidearm chamber (see Figure S1.2 in SI-1); h_1, h_2 and h_3, which were measured at the average distances of 13.5 mm, at 28.5 mm and at 43.5 mm respectively from the root barrier mesh (see Figure S1.2 in SI-1). Table S2.1 below shows the hyphal length density values acquired in this process. Also presented within this table are the standard deviation values for each of the test results computed using the 40 subset samples.

It should be noted that the Jakobsen method hyphal length density values obtained for sample sets P+ T2 (replicate one and two) appear to be identical. Similarly, the values obtained for sample sets P- T4 (replicate two and three) are also indistinguishable; this is because the accuracy of the Jackobsen method is very low, i.e., the method is corase and unable to detect small changes. The data sets which comprise these repeated values are highlighted in yellow and green respectively in Table S2.1.

	h1 = 13.5 mm	h2 = 28.5 mm	h3 = 43.5 mm
**Condition**	**ρ ($mm.mm^{-3}$)**	**ρ ($mm.mm^{-3}$)**	**ρ ($mm.mm^{-3}$)**
P-, T2	9.59 ± 5.3	9.59 ± 3.6	8.95 ± 1.4
P+, T2	8.19 ± 4.0	8.16 ± 4.0	19.29 ± 1.7
P-, T4	10.46 ± 2.5	16.36 ± 10.1	11.46 ± 2.7
P+, T4	10.49 ± 2.8	9.80 ± 0.8	11.94 ± 3.3

Table S2.1 Mean hyphal length densities obtained using Jakobsen, et al. [3] destructive method with standard deviation values (n=40)

The inbuilt MATLAB '*fmincon*' algorithm was used to fit the hyphal length density model to determine the parameters obtained from the Jakobsen, et al. [3] method destructive

measurements. The algorithm '*fmincon*' finds the minimum of a constrained nonlinear multivariable function. The sum of least squares difference between the data and model normalised with respect to standard deviation was used as an objective function to find the model parameter values.

		mean	±	STD	Values from Schnepf et al. (2008)
k	[cm^{-2} s^{-1}]	4.66*10^{-4}	±	1.758*10^{-4}	2.89*10^{-3}
b	[s^{-1}]	2.07*10^{-6}	±	1.386*10^{-6}	5.79*10^{-7}
v	[cm s^{-1}]	1.64*10^{-5}	±	9.047*10^{-6}	2.89*10^{-6}
**SoS**	-	1.11*10^{-2}	±	7.680*10^{-3}	-
**R^2**	-	9.89*10^{-1}	±	7.680*10^{-3}	-

Table S2.2. Mean and STD values for model parameters overall destructive sampling hyphal length density measurements

To summarise, the mean value in Table S2.2 of the constant tip flux at the root surface, k, is approximately one order of magnitude less than that found/estimated by Schnepf, et al. [2], the mean hyphal tip net birth rate, b, is approximately one order of magnitude greater than the value estimated by Schnepf, et al. [2]. The mean rate of tip movement, v, is also approximately an order of magnitude greater when compared to the value from Schnepf, et al. [2].

1.2.2. Fitting the model to the hyphal length density values obtained through SXRCT

The hyphal length densities were also determined using SXRCT analysis. The samples were analysed at the same locations along the side arm chamber, *i.e.,* h_1, h_2 and h_3. 10 values were clustered at each of the positions h_1, h_2 and h_3 respectively.

In contrast to the hyphal length density results obtained using the Jakobsen destructive method (see Table S2.1), all the data sets obtained using SXRCT analysis were distinct; *i.e.* the hyphal length density results obtained for P+, T_2 (replicate one) using SXRCT analysis were different from the hyphal length density results obtained for P+, T_2 (replicate two) using SXRCT analysis. Similarly, when the results from P- T4 (replicate two) sample are compared to the results from P- T4 (replicate three) the hyphal length density values are different. The length densities obtained at h_1, h_2 and h_3 using the SXRCT analysis are presented in Table S2.3.

**Sample set**	**h_1 = 13.5mm ρ (mm·mm^{-3})**	**h_2 = 28.5mm ρ (mm·mm^{-3})**	**h_3 = 43.5 mm ρ (mm·mm^{-3})**
P- T_2 (replicate two)	0.07	0.08	0.0636
P+ T_2 (replicate one)	0.99	0.48	0.2185
P+ T_2 (replicate two)	0.01	0.44	0.2459
P- T_4 (replicate two)	2.32	0.02	0.0423

P- T_4 (replicate three)	0.01	0.05	0.1382
P+ T_4 (replicate two)	0.04	0.01	0.0298

Table S2.3 Hyphal length densities at positions h_1, h_2, and h_3 obtained by SRXCT

		**mean**	**±**	**STD**	**Values from Schnepf et al (2008)**
k	[cm^{-2} s^{-1}]	$1.02*10^{-4}$	±	$1.767*10^{-4}$	$2.89*10^{-3}$
b	[s^{-1}]	$5.45*10^{-6}$	±	$1.273*10^{-5}$	$5.79*10^{-7}$
v	[cm s^{-1}]	$2.06*10^{-5}$	±	$2.566*10^{-5}$	$2.89*10^{-6}$
SoS	-	$7.66*10^{-1}$	±	$2.53*10^{-1}$	-
R^2	-	$2.34*10^{-1}$	±	$2.53*10^{-1}$	-

Table S2.4 Mean and STD values for model parameters obtained using SXRCT hyphal length density measurements

The mean value of the constant tip flux (see Table S2.4) at the root surface, k, estimated based on SXRCT measurements is an order of magnitude lower compared to the results from Schnepf, et al. [2]. The mean values of v and b are approximately an order of magnitude greater when compared to the results from Schnepf, et al. [2].

2. Model for hyphal P uptake rate using hyphal length density results

P values obtained using XRF were used to fit the hyphal P uptake model of Schnepf and Roose [1]. The parameter value fitted to the data was the P uptake by the mycorrhizal hyphae per unit volume of soil (λ). The fits were achieved using parameters generated from the hyphal length density model[2] as presented in Section 1 above. The hyphal length densities were obtained from SXRCT analyses and from the Jakobsen, et al.[3] test results; both have been presented in the previous section.

2.1. Model

The total surface area *A* of hyphae per unit volume of soil $[\mathrm{cm}^2\ \mathrm{cm}_{\mathrm{soil}}^{-3}]$ is given by,

$$A = 2\pi r_m \rho \qquad \text{(S2.14)}$$

Here r_m [cm] is the radius of the hyphae and ρ $[\mathrm{cm}\ \mathrm{cm}_{\mathrm{soil}}^{-3}]$ is the hyphae length density in the soil calculated following the model of Schnepf, et al. [2], as described in the previous section.

Following Schnepf and Roose [1], the nutrient influx into a unit surface area of hyphae, F_{myc} $[\mu\mathrm{mol}\ \mathrm{cm}^{-2}\ \mathrm{s}^{-1}]$ is given by,

$$F_{myc} = \frac{F_{max,m} C_l}{K_m + C_l}. \quad \text{(S2.15)}$$

Here $F_{max,m}$ [µmol cm^{-2} s^{-1}] is the maximal influx rate into the unit surface area of mycorrhizal hyphae, C_l [$\mu\text{mol cm}_{\text{water}}^{-3}$] is the solution concentration of P in the soil, and K_m [$\mu\text{mol cm}_{\text{water}}^{-3}$] is the Michaelis-Menten constant for mycorrhizal hyphae P uptake.

Combining (S2.14) and (S2.15) the total uptake of P by mycorrhizal hyphae per unit volume of soil, R_{myc} [$\mu\text{mol cm}_{\text{soil}}^{-3}\,\text{s}^{-1}$] is given by

$$R_{myc}(x, t; C_l) = 2\pi r_m \rho(x, t)\, \frac{F_{max,m} C_l}{K_m + C_l}. \quad \text{(S2.16)}$$

Following Schnepf and Roose [1] we assume $C_l \ll K_m$, consistent with the assumption of low available P in the soil pore space we can simplify equation (S2.16) by linearizing,

$$\frac{F_{max,m} C_l}{K_m + C_l} \cong \left[\frac{F_{max,m}}{K_m}\right] C_l.$$

Total P in the soil C_{tot} is the sum of P in the solution $(C_l\theta)$ plus the amount of P bound to the soil particles $(C_l b_p)$ and hence

$$C_{tot} = C_l(\theta_l + b_p),$$

where b_P [$\text{cm}^3\ \text{cm}_{\text{soil}}^{-3}$] is the soil buffer power, θ_l [$\text{cm}_{\text{water}}^3\ \text{cm}_{\text{soil}}^{-3}$] is the water volume fraction in the soil.

Then the total uptake of P by fungi per unit volume of soil is given by

$$R_{myc}(x, t; C_{tot}) = 2\pi r_m \rho(x, t)\, \lambda C_{tot},$$

where λ [cm s^{-1}] is the linear influx rate of P into the mycorrhizal hyphae per unit volume of soil given by

$$\lambda = \left[\frac{F_{max,m}}{K_m(\theta_l + b_p)}\right]. \quad \text{(S2.17)}$$

After Schnepf and Roose [1], the equation for the total P concentration in soil C_{tot} is given by

$$\frac{\partial C_{tot}}{\partial t} = \nabla \cdot \left(D_{eff} \nabla C_{tot}\right) - 2\pi r_m \rho(x, t)\, \lambda C_{tot}, \quad \text{(S2.18)}$$

D_{eff} [$\text{cm}^2\ \text{s}^{-1}$] is the diffusion coefficient of P in soil pore water given by

$$D_{eff} = \frac{\theta_l D_l f_l}{\theta_l + b_p},$$

where D_l is the P diffusion in free water, f_l is the geometric impedance of the soil pore space to diffusion, and θ_l is the soil water content. D_{eff} is found to have a value of 1.05×10^{-12} m^{2}s^{-1} for this soil based on work by McKay Fletcher et al.[5].

Initial and boundary conditions to solve equation (S2.18) are

$$C_{tot} = C_{tot,0}, \quad t = 0,$$
$$\boldsymbol{r_n}.D_{eff}\nabla C_{tot} = F_{max}C_{tot}, \quad x \text{ on } \partial\Omega_1, \qquad \text{(S2.19)}$$
$$C_{tot} = C_{tot,0} \; as \; |x| \to \infty,$$

where F_{max} [cm s^{-1}] is the total P flux rate from soil into the root $\boldsymbol{r_n}$ is the unit normal of the root and $\partial\Omega_1$is the root surface.

2.2. Total P concentration values from XRF analysis

The C_{tot} values obtained using XRF were initially presented as counts. These values were converted to values with units of mol cm^{-3}. The procedure to convert the values is outlined below.

The fertilizer used in these experiments was Triplesuperphosphate (TSP). The chemical formula for TSP is $Ca\,(H_2\,PO_4)_2{\cdot}H_2O$. The atomic mass of TSP is, 40+4+62+128+2+16 = 252. P constitutes approximately 25% of the fertilizer pellet, $\frac{62}{252} \approx 0.25$. I gram of TSP with an atomic mass of 252 is equivalent to 0.003968 mol g^{-1}. The density of TSP is 2.09g cm^{-3}. Then,

0.25 X 0.003968 X 2.09 = 0.00207 mol cm^{-3} of P in the fertilizer pellet.

It is determined from the XRF scans that there are 168277 counts per unit volume of P in the fertilizer pellet. Then,

168277 is equivalent to 0.00207 mol cm^{-3}.

Therefore 1 count is equal to a concentration of $1.23\text{x}10^{-8}$ mol cm^{-3}. We use this conversion factor to transform P count values into values with units of mol cm^{-3}.

Figure S2.1 shows the results of the XRF measurements and the comparison between different treatments. A two sample t-test was first performed followed by the Kolmogorov-Smirnov test. These tests were carried out to a significance level of α=0.05. Following both these tests there is sufficient evidence to reject the null hypothesis that the distributions are the same at the 5% significance level for each comparison.

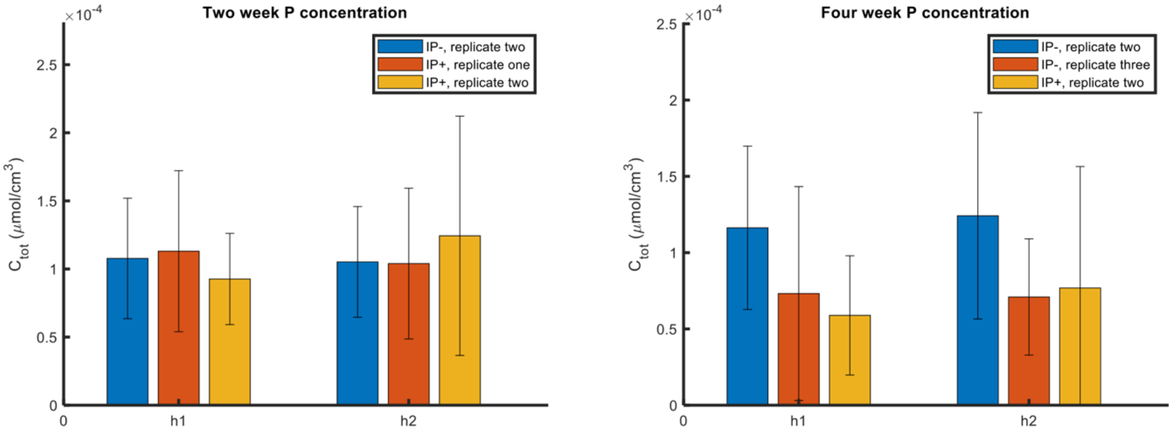

Figure S2.1. C_{tot} values obtained from XRF observations. Error bars show STD.

In addition to the C_{tot} values at position h_1 and h_2, C_{tot} values obtained from XRF scans were available at 16 locations distributed evenly along the hyphal sidearm chamber for each of the samples. These C_{tot} values were each averaged over an area of 0.1208 cm^2. These values have been use to the fit P-uptake model given by equation (S2.18)-(S2.19). The results of these fits are presented in Tables S2.5 and S2.6 using the hyphal length density fitting parameters obtained by the Jakobsen method destructive tests and the SRXCT analysis. In both cases MATLAB 'fmincon' algorithm was used to estimate the hyphal uptake value λ by minimizing the sum of square difference between the model and experimental measurements.

		mean	±	STD	Values from Schnepf et al. (2006)
λ	[cms^{-1}]	1.05*10^{-7}	±	1.120*10^{-7}	3.26*10^{-6}
SoS	-	8.50*10^{-1}	±	1.882*10^{-1}	-
R^2 fit	-	1.05*10^{-1}	±	1.882*10^{-1}	-

Table S2.5 Mean and STD values for P uptake parameter estimated using Jakobsen destructive hyphal density measurement. SOS is sum-of-squares value between the model and experimental data.

From Table S2.5 we can see that the hyphal P uptake value when Jakobsen destructive hyphal density measurement method was used is approximately one order of magnitude less than the value in Schnepf and Roose [1]. This is may be because the Jakobsen measurement registered non-mycorrhizal fungi within soil.

		mean	±	STD	**Values from Schnepf et al. (2006)**
λ	[cms^{-1}]	$3.78*10^{-6}$	±	$6.756*10^{-6}$	$3.26*10^{-6}$
SoS	-	0.955	±	$8.599*10^{-2}$	-
R^2 fit	-	0.045	±	$8.599*10^{-2}$	-

Table S2.6 Mean and STD values for P uptake parameters estimated using SRXCT hyphal length density measurements. SOS is sum-of-squares value between the model and experimental data.

From Table S2.6 we can see that the hyphal P uptake value obtained by Schnepf and Roose [1] agrees very well with the mean hyphal P uptake value obtained with this model.

We conclude based on these estimates for hyphal uptake values that the level of active mycorrhizal hyphae is the crucial parameter linking the uptake rate from soil. Thus, a new methodology for visualisation of mycorrhizal hyphae in the mixed phase should be developed as the estimates using different hyphal length density measurements influence the hyphal uptake rate by one order of magnitude. However, with this study we have narrowed the range of possible hyphal uptake values to within one order of magnitude. This will help to further improve any follow on experiments.

References

1 Schnepf, A. & Roose, T. Modelling the contribution of arbuscular mycorrhizal fungi to plant phosphate uptake. *New Phytologist* **171**, 669-682 (2006).

2 Schnepf, A., Roose, T. & Schweiger, P. Growth model for arbuscular mycorrhizal fungi. *Journal of The Royal Society Interface* **5**, 773-784, doi:10.1098/rsif.2007.1250 (2008).

3 Jakobsen, I., Abbott, L. & Robson, A. External hyphae of vesicular ‐ arbuscular mycorrhizal fungi associated with Trifolium subterraneum L. *New Phytologist* **120**, 371-380 (1992).

4 Gerald, C. F. & Wheatley, P. O. *Applied numerical analysis*. (Addison-Wesley Pub. Co., 1994).

5 McKay Fletcher, D. M., Keyes, S. D., Daly, K. R., van Veelen, A. & Roose, T. A multi-image based modelling of plant-fertilizer interaction. *Rhizosphere* (2019).
